# Archaeological science, globalisation, and local agency: gold in Great Zimbabwe

**DOI:** 10.1007/s12520-023-01811-7

**Published:** 2023-08-03

**Authors:** Jasmine Vieri, Shadreck Chirikure, Paul Lane, Marcos Martinón-Torres

**Affiliations:** 1 McDonald Institute for Archaeological Research, University of Cambridge, Downing Street, Cambridge, CB2 3ER UK; 2 Research Laboratory for Archaeology and the History of Art, School of Archaeology, University of Oxford, 1 South Parks Road, Oxford, OX1 3TG UK; 3 Department of Archaeology, University of Cape Town, Private Bag X3, Rondebosch, 7701 South Africa; 4 Department of Archaeology, University of Cambridge, Downing Street, Cambridge, CB2 3DZ UK; 5 School of Geography, Archaeology & Environmental Studies, University of the Witwatersrand, 1 Jan Smuts Avenue, Braamfontein 2000, Johannesburg, South Africa

**Keywords:** Great Zimbabwe, Globalisation, Local agency, Indian Ocean exchange, Gold metallurgy, Technology

## Abstract

Great Zimbabwe (CE1000–1600) is world famous for outstanding cultural innovations and localised and globalised entanglement with trans-Africa and trans-Indian Ocean exchange. New excavations yielded fragments of over a hundred gold processing vessels comprising reused pottery and purpose-made crucibles from stratified contexts in the Eastern Ridge Ruins and adjacent areas. Selected samples were studied using archaeological, microscopic, and compositional (SEM–EDS) techniques. All ceramics were made of alumina-rich clays and contain minerals common to granite-derived lithologies typical of the area, although it is possible that particularly refractory clays were selected to make crucibles locally. These technical ceramics were used for refining and collecting gold at high temperature, most likely producing not only relatively standardised ingots but also finished objects. The composition of the gold prills set in crucible slag is consistent with that of natural, unalloyed gold, while the variability in silver levels and minor impurities point to heterogeneous sources of the gold. Considering these finds in their multiple site and regional contexts, and together with complementary threads of information from early reports of antiquarians and looters, we argue that local agency and gold consumption were much more significant than generally assumed. The conclusion to the paper is that Great Zimbabwe’s famous participation in local and global exchanges was backed by internally driven but improvisation laden production and consumption occurring in homesteads located throughout its various settlements. We end by raising a word of caution about oversimplified narratives of globalisation and their archaeological expressions (see Supplementary Material S0 for the abstract in Shona).

**Supplementary Information:**

The online version contains supplementary material available at 10.1007/s12520-023-01811-7.

## Introduction

The multidirectional exchanges between southern Africa and the wider Indian Ocean region rank as a prominent example of early forms of terrestrial and maritime local and global interconnections (Mudenge 1988; Chaudhuri 1990; Pwiti 1991; Kusimba 1999; LaViolette 2008; Seetah 2018). Great Zimbabwe (20°16′S 30°56′E﻿), situated approximately 28 km to the southeast of Masvingo and about 300 km as the crow flies from the Indian Ocean Coast, participated with varying degrees of intensity in commodity circulations in southern Africa and the wider Indian Ocean for six centuries (1000 to 1600 CE) (Caton-Thompson 1931; Sinclair 1987; Swan 1994; Pikirayi 2002; Chirikure et al. 2018). Cumulative research has shown that the settlements of Great Zimbabwe were not built as a single unit; neither were they constructed following a predetermined spatial plan (Chipunza 1994; Beach 1998; Ndoro 2001). Rather, occupation started on the hill flowing onto the terraces and, after a few centuries, extended into the valley below (Summers et al. 1961). From there, expansion reached the eastern edges and the western end towards the current Great Zimbabwe Hotel (Sinclair et al. 1993). The expansion created a series of dispersed clusters of multi-building settlements on the hill and its contours, in the Valley and Great Enclosures, in the unwalled settlements in between the cluster of walls, and smaller walled settlements such as Chenga (Chirikure 2020a). This created a vast network of households and homesteads in various areas, creating an urban formation locally known as *guta* (city) (*maguta*: plural).

The discovery of staggeringly productive auriferous deposits on the Rand in South Africa in the 1880s prompted Cecil John Rhodes to colonise territory between the Zambezi and Limpopo Rivers in anticipation of rich and diverse gold deposits (Swan 1994; Mugumbate et al. 1999, 2000; Ndoro 2001; Fontein 2006). Previously, the post-sixteenth century Portuguese speculators wrongly believed that the Zimbabwe plateau was the principal source of gold for the Queen of Sheba and the biblical King Solomon (Garlake 1973; Hall 1987). Karl Mauch and other late nineteenth century Euro-American explorers bought into the speculation and concluded that Great Zimbabwe was not built by Africans. The Ancient Ruins Company was established (by Rhodes or his associates) to prospect for gold at Great Zimbabwe and related places, making huge discoveries of gold objects and ingots (see Hall and Neal 1902 for inventory; Miller 2002). Often, the finished gold objects and infrastructure for processing it were destroyed without record. As a result of looting of gold and the tardy development of research interest into gold working, not much is known about the beginning of gold working and the evolution of technologies used to process this metal (see Swan 1994 for a summary of early studies). While trade between southern Africa and the Indian Ocean started around 500 CE, the earliest written reports mention that by the early twelfth century CE, gold was an established export from the coastal Swahili settlements into the Indian Ocean networks (Mudenge 1988; Swan 1994; Miller et al. 2000). So far, the earliest indirect evidence of gold production at Great Zimbabwe (and in the interior) appears in the form of specialised crucibles recovered from eleventh and twelfth century contexts on the Hill Complex (Bandama et al. 2016). The excavator (Robinson 1961, p. 213) clearly misidentified the crucible as a “model bowl”. Before the advent of gold and bronze, both derivatives of the Indian Ocean connections, repurposed domestic pottery was used to smelt and melt copper in southern Africa (Bisson 2000). However, by 800 CE purpose-made crucibles were in use at Chibuene on the Indian Ocean coast. The 2.3 kg of gold grave goods recovered from Mapungubwe burials date to the mid-thirteenth century (Meyer 1998; Miller et al. 2001), nearly a century after initial reports of gold working in East Africa (Miller et al. 2000) and after purpose-made crucibles were in use at Great Zimbabwe (Miller et al. 2001; Bandama et al. 2016). So far, Mapungubwe has not yielded evidence of gold-processing crucibles or reused pottery (Calabrese 2000; Miller et al. 2001; Chirikure et al. 2015). However, repurposed domestic pottery sherds with prills of gold are known from multiple sites coeval with Mapungubwe such as Marcadon Claim (Swan 1994).

Overall, there is more information on gold mining in southern Africa (Mennell 1934; Summers 1969; Phimister 1974; Swan 1994; Miller et al. 2000) than there is on the pyrometallurgy of gold as reconstructed from production or processing remains. Swan (1994, pp. 64–68) combined information from archaeological, historical, and archival sources and performed XRF analyses of crucible residues, providing exploratory understandings of gold production at selected sites located between the Limpopo and Zambezi rivers. She argues for alluvial gold mining during the first millennium CE and reef gold mining after 1000 CE. Overall, Swan places more weight on external agency and stimuli for gold production (mostly extracted for trade), a position that has since been challenged (Bandama et al. 2016; Moffett and Chirikure 2016). Other than Swan, Stanley (1931) analysed metal objects from Caton-Thompson’s Great Zimbabwe excavations, while Oddy (1984), Miller (2001, 2002), Miller (2001), Grigorova et al. (1998), and Netshitungulwana (2011) explored the working history, manufacturing techniques, and provenance of samples of gold artefacts from Great Zimbabwe, Mapungubwe, and other sites.

This paper presents the results of integrated material analyses of the largest collection of gold-processing crucibles ever recovered from undisturbed contexts in southern Africa. For the first time, it characterises the manufacture, material properties, and performance characteristics of technical ceramics (crucibles and reused sherds), gold processing technology, and metalworking conditions. When placed within the wider historical frame, the scientific data allow new revelations and reconfiguration of narratives on gold, internally within Great Zimbabwe and externally within southern Africa and the Indian Ocean. The next section presents the geological and pedological conditions at Great Zimbabwe and the surrounding landscape as a step towards establishing genetic relationships between technical and domestic ceramics and the local environment, and chemically correlating gold to possible sources.

## Geological and pedological background of Great Zimbabwe and the surrounding area

**Fig. 1:**
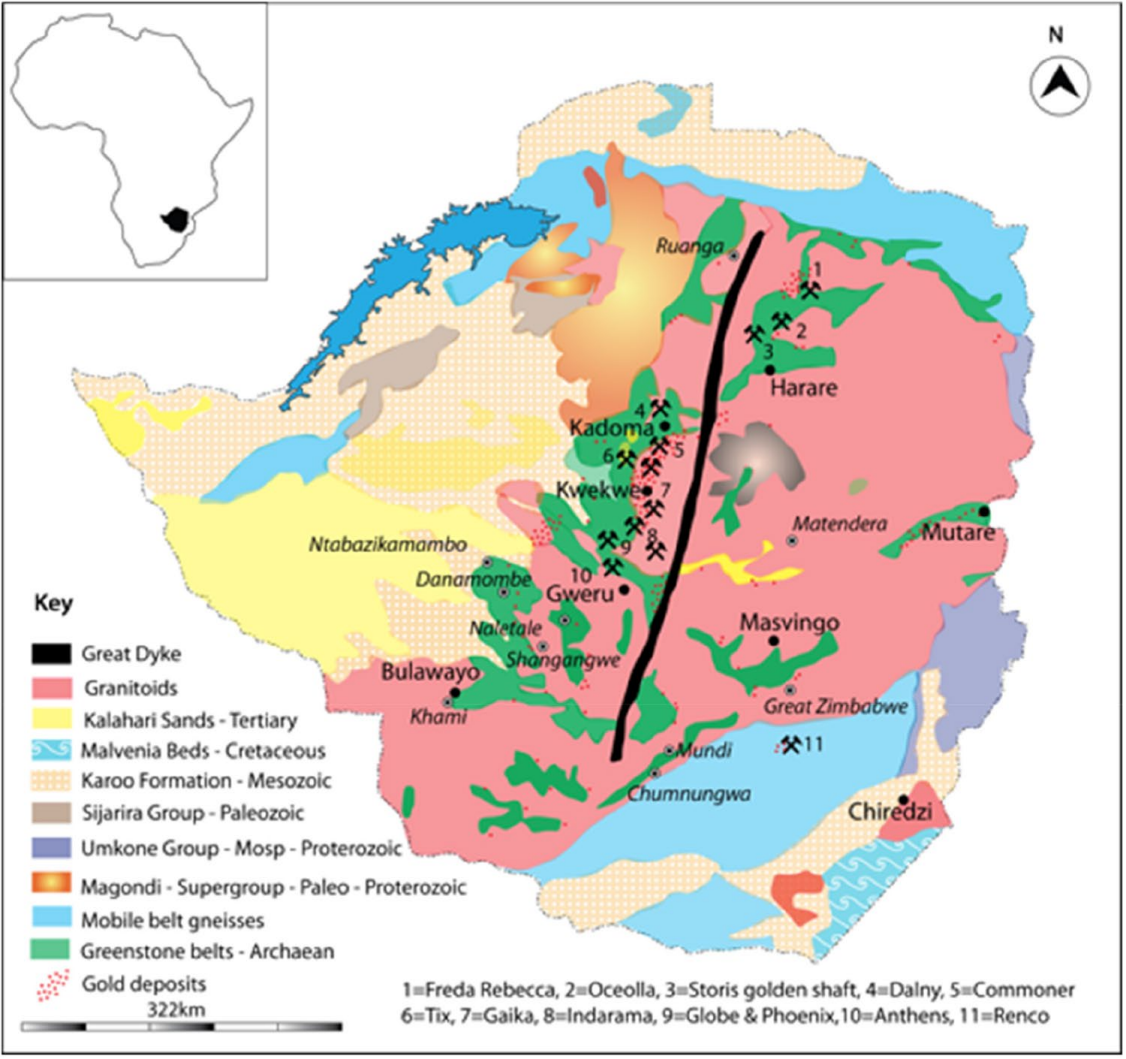
Geological map of Zimbabwe showing gold hosting Archaean greenstone belts (modified after Mugumbate et al. 1999/2000; redrawn by Robert Nyamushosho)

Studying the technical properties of crucibles and records of gold-processing set in them requires an understanding of the geology and pedological conditions in and around the study area. According to Mugumbate et al. (1999/2000), nearly 60% of Zimbabwe’s land surface is composed of granitic rocks deposited in an Archaean-age (pre-2550 Ma) basement known as the Zimbabwe Craton. The granitic rocks encircle gold-hosting sedimentary formations known as greenstone belts (Gwavava and Ranganai 2009). Rivers that drain greenstone belts are rich in alluvial gold (Mukandi 1999/2000). Great Zimbabwe is located on the northern edge of the late granites of the Charumbira Pluton adjacent to their contact point with the auriferous Masvingo Greenstone belt (Fig. 1). The Masvingo and other nearby greenstone belts, such as the Mashava and Mberengwa greenstone belts, contain mafic and ultramafic metavolcanics; banded iron formations; and pelites, psammites, and carbonates that stratigraphically belong to the following ages: Sebakwian (3500 Ma), Upper Bulawayan (2700 Ma), and Shamvaian (2650 Ma) (Wilson 1964, 1979; Prendergast and Wingate 2007; Gwavava and Ranganai 2009; Chaumba 2019). The main rock types are banded ironstone (quartz-magnetite schist), hornblende schist (and associated amphibolite), serpentine, and derived schist (Chaumba 2019). The gold of southern Zimbabwe, like that of elsewhere in the country, mineralised in greenstone belts and their immediate granitoid surroundings near Great Zimbabwe (Naden et al. 1994; Mugumbate et al. 1999/2000; Mukandi 1999/2000) (Fig. 1). According to Phimister (1976, pp. 13–14), indigenous people had an array of geobotanical criteria which they applied to identify mineral deposits. Unsurprisingly, more than two-thirds of the 6000 gold deposits known in Zimbabwe were previously worked by Africans before European colonisation (Mennell 1934; Summers 1969; Swan 1994; Mugumbate et al. 1999, 2000).

**Fig. 2:**
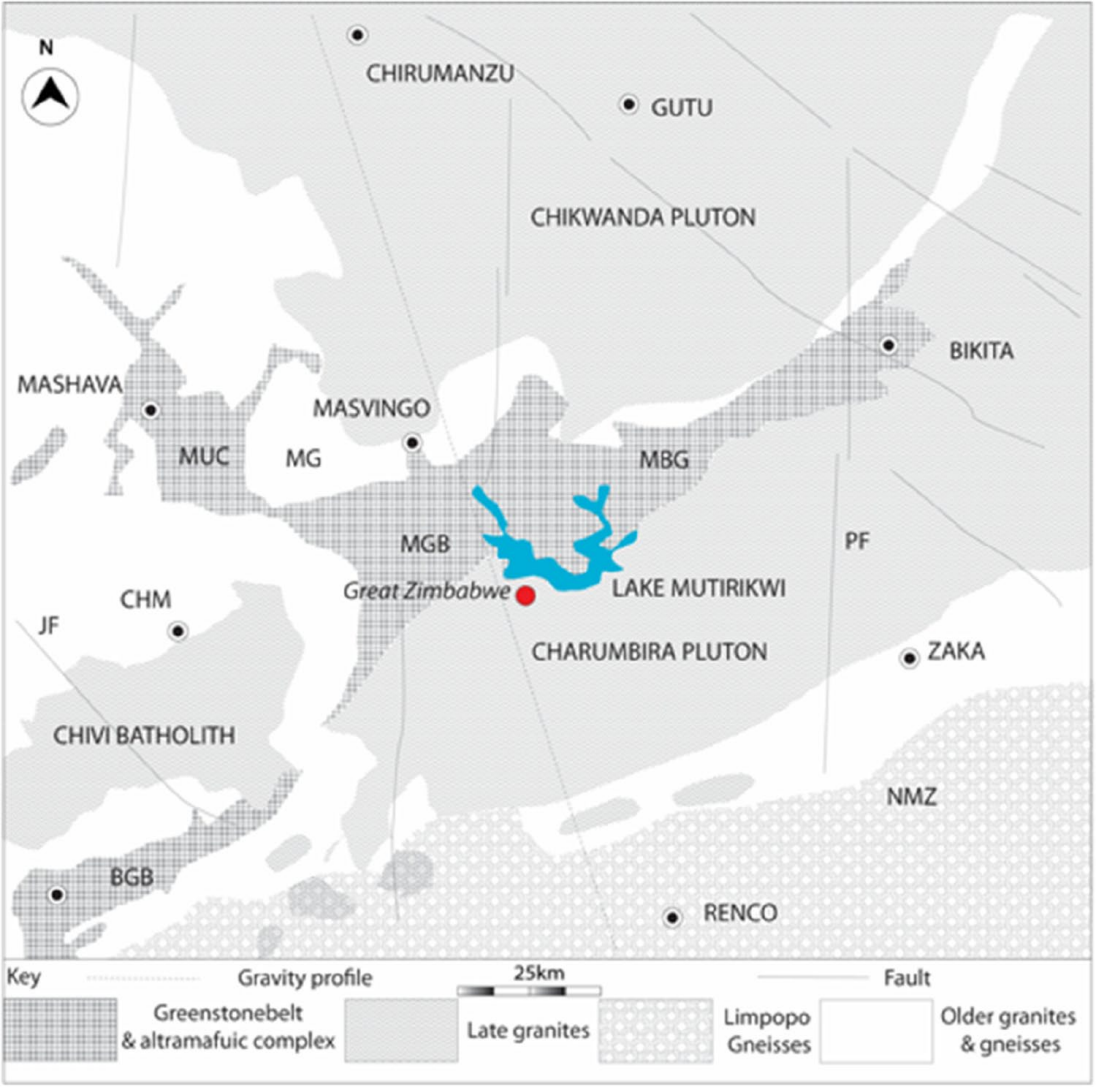
Geological map of southern Zimbabwe showing the area around Great Zimbabwe and the regional geology (modified after Gwavava and Ranganai 2009, p. 279; redrawn by Robert Nyamushosho). BGB is Buhwa Greenstone Belt; MGB is Masvingo Greenstone Belt; MG is Mushandike Granite; MUC is Mashava Ultramafic Complex; NMZ is northern Marginal Zone of the Limpopo Belt; JF is Jenya fault; PF is Popoteke Fault

Naden et al. (1994) studied gold mineralisation in Zimbabwe using bedrock samples from nine mines located in the major gold-producing areas of Mazowe, Kadoma, Kwekwe, Mberengwa, Chegutu, and Chirumanzu (Figs. 1 and 2). Some of these deposits were worked in ancient times, making them possible sources for Great Zimbabwe’s gold (Summers 1969; Phimister 1976). In general, gold mineralisation is classified into strata-bound and non-strata-bound categories. Strata-bound include deposits hosted in iron formations, mineralisation in banded sulphides, and deposits in volcaniclastic and clastic formations (Naden et al. 1994). Mineralisations hosted in veins and shear zones constitute the non-strata-bound type. Vein-type deposits consist of quartz, carbonates, and minor sulphides. The variation in chemistry and inclusion mineralogy of Zimbabwe gold deposits broadly correlate with gold composition and geological environment. For example, high-silver gold (> 10 wt% Ag) and inclusion mineralogy containing tellurides was associated with granite and metasediment-hosted deposits, while low-silver gold (< 10 wt%) plus an inclusion mineralogy containing nickel and cobalt minerals correlated with deposits hosted in basic and ultra-basic rocks (Foster 1985; Naden et al. 1994). The direct correlation between occurrence of gold and different minerals could potentially point to sources of Great Zimbabwe’s gold (Table 1).

**Table 1:** Ore mineralogy, host rocks, and lithology types associated with some gold deposits (based on a modification of Table 2, Naden et al. 1994: 44)

Type of deposit	Mine	Region	Ore mineralogy	Host rocks	Lithology type	Stratigraphy
Vein	Commoner	Kadoma	Tellurides, pyrite, galena, chalcopyrite, sphalerite, tennantite-tetrahedrite	Greywackes, phyllites and andesites	Sedimentary	Calc-alkaline
Vein	Tix	Kadoma	Galena, sphalerite, pyrite	Granitic gneiss	Granitic	Rhodesdale batholith
Vein	Gaika	Kwekwe	Pyrrhotite, stibnite, chalcostibite, gold	Magnesite rock	Ultramafic	Kwekwe-Ultra mafic
Vein	Globe and Phoenix	Kwekwe	Stibnite, arsenopyrite, pyrite, sphalerite, galena	Globe granite Phoenix–Magnesite schist	Granitic	Rhodesdale Batholith Kwekwe ulta-mafic complex
Vein	Indarama	Kwekwe	Pyrite, pyrrhotite, arsenopyrite, stibnite, tetrahedrite, gold, gudmundite, berthierite, aurostibite	Mafic lavas	Mafic	basaltic
Vein	Storis golden shaft	Mazoe	Pyrite, pyrrhotite, chalcopyrite	Epidiorites		
Vein	Oceolla		Pyrrhotite, pyrite, galena	Mafic lavas	Mafic	Basaltic
Shear Zone	Dalny	Chegutu	Arsenopyrite, pyrite, stibnite, galena, chalcopyrite, sphalerite, scheelite, tennantite-tetrahedrite	Andesites	Mafic	Basaltic
Shear Zone	Anthens	Mvuma	Pyrite, pyrrhotite, chalcopyrite, galena, sphalerite, arsenopyrite, bismuthite, gersdofite	Talc-chlorite and tremolite schists	Ultramafic	Basaltic

**Table 2:** Radiocarbon dates from Great Zimbabwe, calibrated at 2σ using rcarbon v.1.4.1 (Crema and Bevan 2021) and rounded to the nearest 5 calendar years

Context number (area)	Sample number (material)	Uncalibrated date	Calibrated range 1 (%)	Calibrated range 2 (%)
GZERRM1 L1 (Eastern Ridge Ruin Midden)	Beta: 447824 (charred material)	590 ± 30 BP	cal AD 1325–1350 (21%)	cal AD 1390–1435 (73%)
GZERRM1 L3 (Eastern Ridge Ruin Midden)	Beta: 447823 (charred material)	670 ± 30 BP	cal AD 1295–1395 (95%)	NA
GZERRM1 4B (Eastern Ridge Ruin Midden)	Beta: 444648 (charred bone organics)	610 ± 30 BP	cal AD 1320–1355 (43%)	cal AD 1385–1430 (52%)
GZERRM1 L2 (Eastern Ridge Ruin Midden)	Beta: 443769 (charred material)	590 ± 30 BP	cal AD 1325–1350 (21%)	cal AD 1390–1435 (73%)
GZERRM1 L5 (Eastern Ridge Ruin Midden)	Beta: 443767 (charred material)	580 ± 30 BP	cal AD 1325–1345 (12%)	cal AD 1390–1440 (83%)
URM1 TBL4 (Upper Ridge)	Beta: 447830 (charred material)	570 ± 30 BP	cal AD 1325–1340 (5%)	cal AD 1390–1445 (95%)
GZUR1 TB2 (Upper Ridge)	Beta: 443770 (charred material)	560 ± 30 BP	cal AD 1330–1335 (1%)	cal AD 1395–1450 (94%)
GZUR1 TB3 (Upper Ridge)	Beta: 443768 (charred material)	600 ± 30 BP	cal AD 1320–1355 (33%)	cal AD 1385–1430 (62%)
Fireguard M1T1L1 (Fireguard Midden 1)	Beta: 447827 (charred material)	460 ± 30 BP	cal AD 1430–1505 (84%)	cal AD 1595–1615 (11%)

Compositional analyses of the gold from the different Zimbabwean mines identified major, minor, and trace elements in the gold from various deposits. Factoring within source variation, it appears that some of the deposits are polymetallic and host gold associated with silver (2–22 wt%) and traces of As, Se, S, Pd, Sb, Te, Fe, Cu, Hg, Bi, and Pb (Naden et al. 1994; Kisters et al. 1998; Mukandi 1999/2000; Blenkinsop et al. 2000; Klemm and Kräutner 2000).

The geology and associated rock-forming minerals weathered to create a wide range of local soils and clays at Great Zimbabwe and elsewhere that vary considerably in their physical, chemical, and mineralogical compositions (Purves 1976). However, around 46% of Zimbabwe’s soils are of granitic origin (Purves 1976; Nyamapfene 1983). Overlaying geological and soils maps of Zimbabwe at the same scale shows a very high degree of correspondence between parent geology and soil types (Nyamapfene 1991). The soils around Great Zimbabwe belong to the kaolinitic Orthoferallitic group (class 7G). According to Nyamapfene (1983), clay fractions in this soil type are very inert and are predominantly made up of kaolinite and related low activity clays mixed with oxidic clay materials such as haematite, gibbsite, and goethite. The clay content is generally moderate to high, although the clay sometimes lacks stickiness. Africans had a comprehensive system of recognising, describing, and classifying soils and edaphological conditions in their environment (Nyamapfene 1991). The classification combined sensory and textual variables including colour, coarseness, smell, and taste. Clays for making ceramics, both for household use and other technical purposes, were generally “infertile” and are known as *chinamwe/rondo/chiombwe*. Often, such clays are light grey in colour and are associated with specific vegetation such as *mukute* (*Syzygium* spp.), *muroro* (*Annona stenophylla*), and *mutsamviringa* (*Ficus ingens*) (Nyamapfene 1983). The genetic relationship between local rocks and soils supports the proposition that if Great Zimbabwe’s crucibles and pottery were made of local clays, then their mineralogical and chemical composition must correspond to local geological and pedological conditions.

## Excavations and chronology: Eastern Ridge Ruins [Mauch Ruins] Midden and adjacent Upper Ridge areas

### Fieldwork protocols

**Fig. 3:**
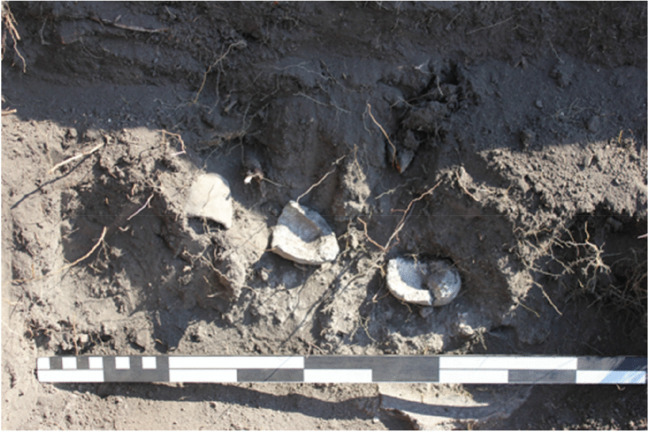
Photograph of Fireguard Midden excavations showing crucibles being exposed during excavations

In 2016, archaeological excavations at Great Zimbabwe were directed at unwalled settlements to shift research focus away from drystone-built areas (Chirikure et al. 2018). Dedicated foot surveys identified an open area between 200 and 300 m due east of the Great Enclosure. The area is on the same contour as the ledge where the Eastern Ridge Ruin settlements are situated. Below that, and going northwards, are ridges with settlement remains. Approximately 150–200 m to the east of the Eastern Ridge Ruins are house floors, scatters of potsherds, and middens. A modern fireguard constructed to manage veld fires within Great Zimbabwe, perpendicularly intersects with the Upper Ridge in this area. Vegetation clearance to construct the firebreak exposed house floors and potsherds. A survey datum was established from the eastern edge of the Eastern Ridge Ruins in the west extending 300 m eastwards along the Upper Ridge and crossing the fireguard. The Upper Ridge excavation units were established in this area. A perpendicular north to south offset (50 m long) was taken from the baseline targeting a midden named the Fireguard Midden 1. Previous studies associated this area with a nineteenth century post-Great Zimbabwe occupation (Sinclair et al. 1993), making it imperative to test the hypothesis using empirical observations from excavations. For control purposes, a decision was made to excavate a midden situated 5 m away from the eastern edge of the Eastern Ridge Ruins. The trenches on the Upper Ridge and the Fireguard Midden 1 were excavated to a depth of 60 cm and yielded typical period IV ceramics, pottery, glass beads, animal bone, and crucible fragments. Fireguard Midden 1 was prolific in terms of crucible remains (Fig. 3) and a punched gold bead was recovered there. The Eastern Ridge Midden Trench 1 turned out to be the deepest, reaching 2 m in parts. It yielded crucibles, pottery reused for metallurgical purposes, iron slag, and a large number of ceramics plus animal bone, glass beads, copper-based alloys, iron objects, and a tiny, hammered gold bar. Although artefact analyses are still ongoing, the excavations collectively generated more than 100 technical ceramics, including both purpose-made crucibles and domestic pottery sherds reused for metallurgy. These different styles raised vital questions regarding whether these reaction vessels were used for the same tasks in the processing of gold. This was tested using scientific studies of samples from each category in the laboratory.

### Chronology

**Fig. 4:**
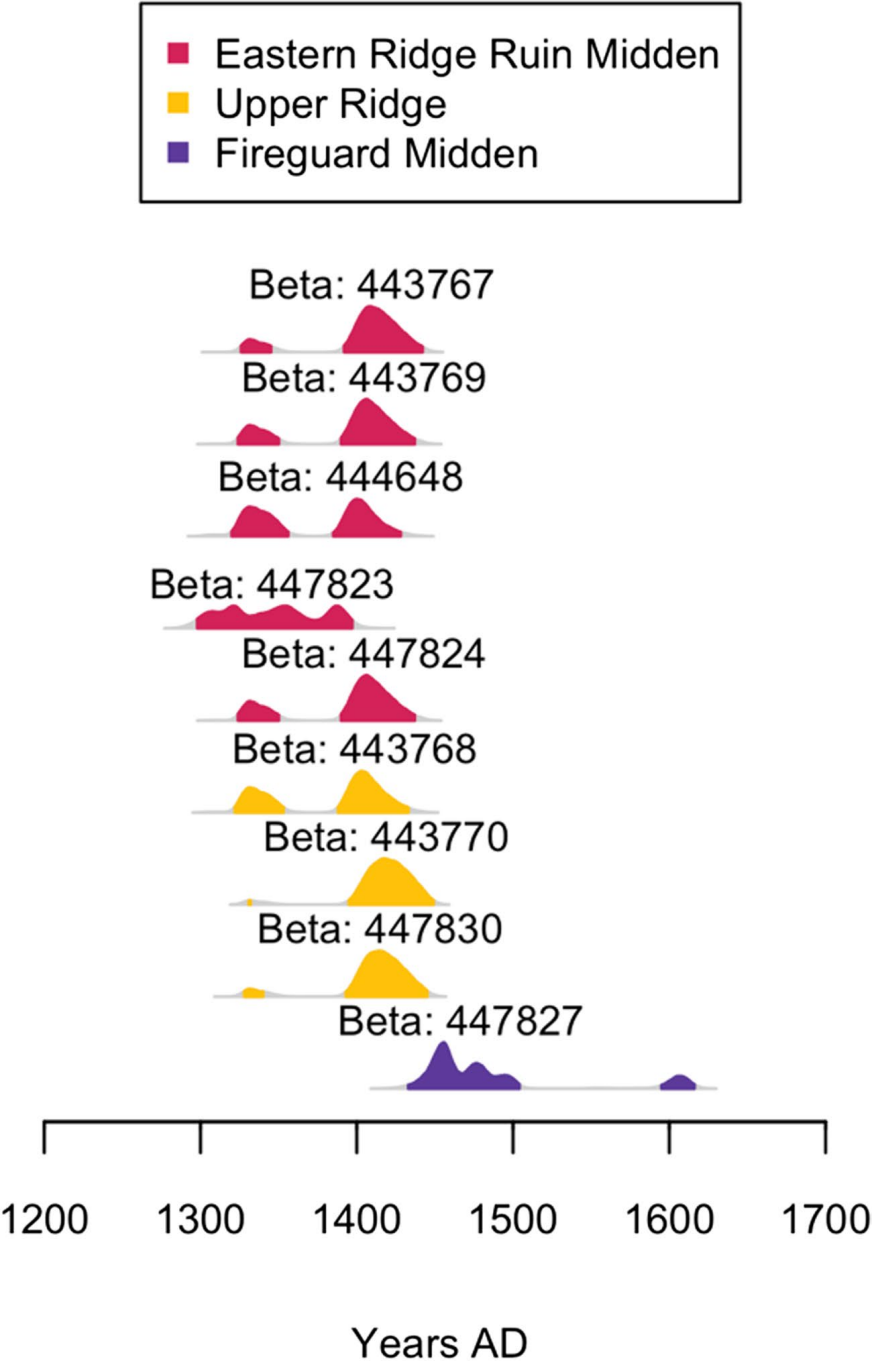
Calibrated radiocarbon dates from Great Zimbabwe (graph created in R v.4.0.2 (R Core Team 2020) using package rcarbon v.1.4.1 (Crema and Bevan 2021))

Nine samples of carbonaceous materials were submitted to Beta Analytic for radiocarbon dating (Table 2, Fig. 4). When calibrated at 2σ with rcarbon version 1.4.1 (Crema and Bevan 2021) using the Southern Hemisphere Curve (Hogg et al. 2020), it became clear that the Upper Ridge area was occupied for a fairly long time. The events represented by the Eastern Ridge Midden 1 were dated from the early fourteenth to the early fifteenth centuries. Only a single date was produced for the Fireguard Midden Trench 1 Layer 1, giving an estimated age range of CE 1430–1505, CE 1595–1615. The Upper Ridge Area and the Fireguard Midden yielded Khami-type glass beads that were not recovered from the Eastern Ridge Ruins. Khami beads date from the fifteenth to seventeenth centuries (Robertshaw et al. 2010). Therefore, the settlements associated with the Eastern Ridge Ruin were occupied earlier, overlapped for a bit with Upper Ridge and adjacent areas, before being abandoned while the later was still the locus of activities.

## Experimental: sample descriptions and analytical protocols

Preliminary sorting of the materials took place in the Great Zimbabwe Conservation Centre. A permit was obtained from the National Museums and Monuments of Zimbabwe to temporarily export 13 samples of reused pottery (5), crucibles (4), furnace wall (1), and domestic pottery (3) (Table 3) for further studies in the Archaeological Materials Laboratory, University of Cape Town, and the Archaeological Sciences Laboratory in Cambridge.

**Table 3:** Macroscopic descriptions of the studied samples

CA laboratory code (sample ID)	Context	Description
Purpose-made		
CA200170 (GZ2)	Eastern Ridge Ruins (120–130 cm)	Crucible with slag with gold prills on the inside; some vitrification on the outside
CA200171 (GZ4)	Upper Ridge Test Pit B4 (40 cm)	Crucible with slag on the mouth and a small prill visible to the naked eye on the inside, with a drip line on the outside
CA200172 (GZ10)	Upper Ridge Test Pit 3	Crucible with friable clay, rock inclusions, and slag on the mouth, inside and outside
CA200173 (GZ12)	Upper Ridge Test Pit 3 (level 4)	Crucible with slag on the mouth, inside and outside
Furnace wall		
CA200178 (GZ7)	Eastern Ridge Ruins	Vitrified furnace wall with frequent coarse quartz inclusions
Domestic repurposed		
CA200174 (GZ1)	Eastern Ridge Ruins (160 cm)	Pottery with vitrification and a layer of slag with gold prills on the inside; also vitrified in broken cross-section
CA200175 (GZ6)	Eastern Ridge Ruins	Pottery, ceramic bloated on the inside, with attached gold prills
CA200176 (GZ8)	Eastern Ridge Ruins, level 15	Pottery with thick black glassy slag with gold prills on the inside; also vitrified in broken cross-section
CA200177 (GZ11)	Eastern Ridge Ruins Midden 1 (level 13)	Pottery with thick black glassy slag with gold prills on the inside
CA200179 (GZ9)	Eastern Ridge Ruins 80–90 cm	Pottery with slag with gold prills on the inside
Domestic pottery		
CA200180 (GZ3)	Eastern Ridge Ruins (120–130 cm)	Pottery, polished on the outside, with visible rock inclusions on the surface
CA200181 (GZ5)	Fireguard Midden	Pottery, polished on the outside
CA200182 (GZ13)	Eastern Ridge Ruins	Pottery, fired to oxidation, burnished on the outside

The original sample IDs assigned to these materials (GZ1–GZ13) overlap with a different set of crucibles from the site, reported in Bandama et al. (2017) and originating from the site’s legacy collections. To avoid confusion, the samples reported here will be referred to by their Cambridge Archaeology (CA) laboratory codes.

**Fig. 5:**
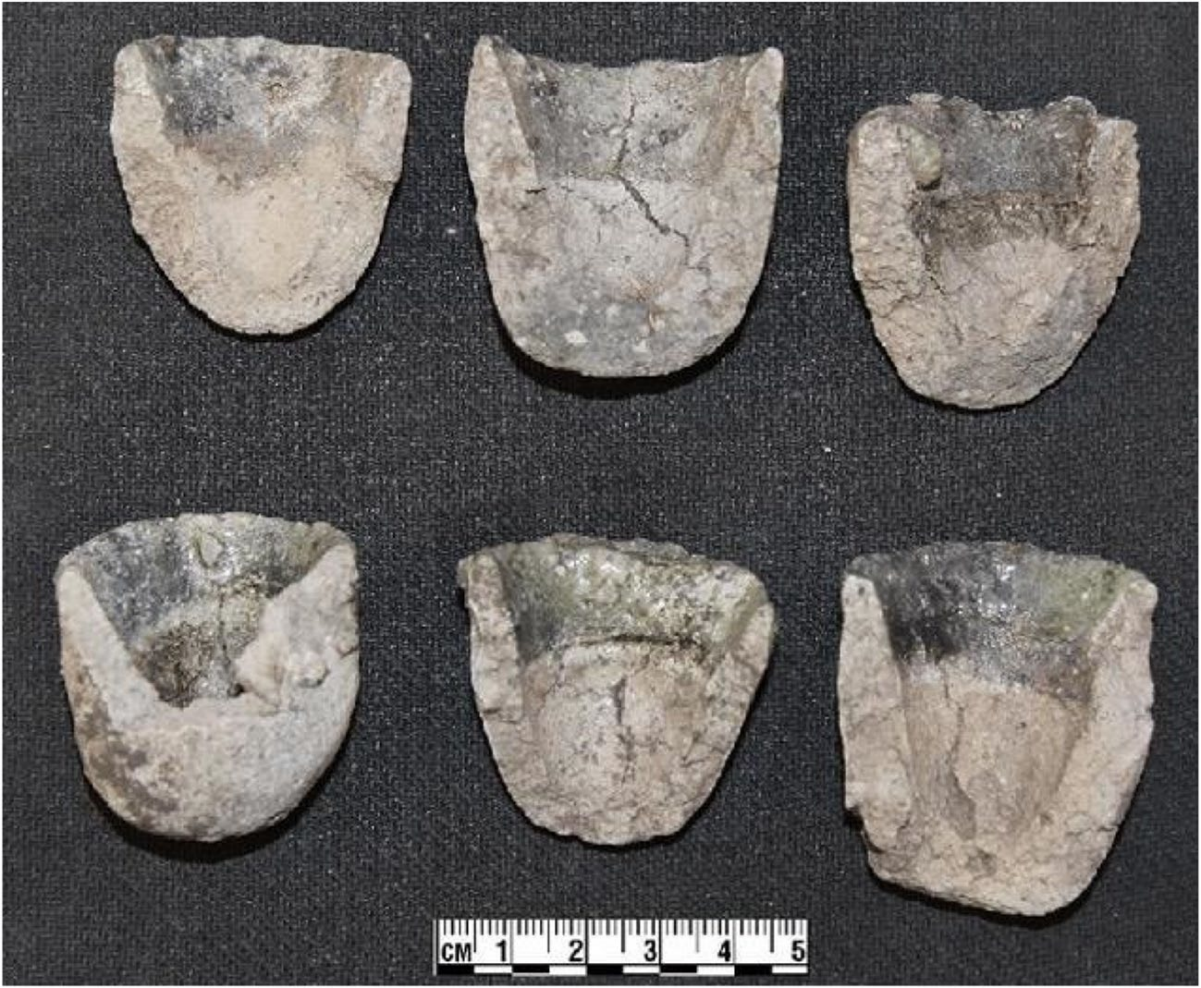
Crucibles from the Fireguard Midden showing slag layers in the top third. Measurements (*x*, *y*, *z* dimensions, see Fig. 18) were taken to estimate the volume and weight of metal below the slag line

Macroscopic observations and exploratory digital microscopy were performed to characterise the paste, inclusions, and metallurgical slagging on unprepared specimens, using a Keyence VHX6000 high-resolution 3D microscope. Variables such as internal and external diameters of semi- and complete vessels, depth, size of crucibles, size of prills, and degrees of vitrification were recorded as appropriate (Fig. 5).

The use of repurposed pottery and purpose-made crucibles to melt gold raised questions about whether the fabrics used to make them had identical properties and why the two groups of vessels were used for what macroscopically appeared to be the same purposes. Furthermore, we sought to compare both types of technical ceramics to domestic pottery from the site. Small samples of repurposed pottery, domestic pottery (for control purposes), and purpose-made crucibles were cut using a diamond saw, mounted as cross-sections in epoxy resin as thick blocks and polished to 1-µm diamond paste finish. These samples were analysed using a Zeiss Gemini SEM 300 scanning electron microscope with a field emission gun and an Oxford Instruments EDS detector, operating at an accelerating voltage of 20 kV, working distance of 8.5 mm, process time 5, acquisition normalised to 700 k counts per spectrum, and 60-µm aperture, with spectra processed using Aztec software. Calibration was undertaken with a cobalt standard, and a certified standard reference material was analysed to test for precision and accuracy (Supplementary Material S1). Single analyses are reported for the inclusions and prills within the ceramic matrices/slags in Supplementary Material S2. Where possible, three separate areas were analysed for the ceramic bulk (large area), matrix (avoiding large inclusions), and slag composition of each sample, with the individual analyses reported in Supplementary Material S3, and with the average of these analyses reported here. All results are reported as weight percentages (wt%) normalised to 100 wt%, with oxygen added by stoichiometry when oxides are reported. Original analytical totals are also presented. Only elements present above 3*σ* of the instrument detection limit are reported here. Where three areas were analysed, but a specific oxide was only detected in two out of three areas, the remaining area was treated as zero in the calculations for the mean, the standard deviation (SD), and the coefficient of variation (CV) reported in Supplementary Material S3. The compositions reported in Tables 4, 5, 6, and S3.1.1–S3.13.1 are reported as ≤ the mean returned by these calculations.

**Table 4:** Composition of the bulk ceramic for all samples (all values reported as normalised to 100 wt%)

Purpose-made	Na_2_O	MgO	Al_2_O_3_	SiO_2_	P_2_O_5_	K_2_O	CaO	TiO_2_	FeO	SnO_2_	BaO	PbO	Analytical total
CA200170	n.d	≤ 0.1	22.5	70.6	n.d	4.0	≤ 0.4	0.6	1.7	n.d	n.d	n.d	74.3
CA200171	n.d	n.d	25.1	67.9	n.d	4.0	≤ 0.3	0.7	1.4	≤ 0.7	n.d	n.d	80.0
CA200172	n.d	n.d	26.9	66.1	n.d	4.1	n.d	0.6	2.2	n.d	n.d	n.d	79.8
CA200173	n.d	n.d	23.2	70.6	n.d	3.9	≤ 0.3	0.7	1.3	n.d	n.d	n.d	74.6
Furnace wall													
CA200178	≤ 0.2	≤ 0.2	26.8	62.4	n.d	3.4	≤ 0.3	0.7	6.0	n.d	n.d	n.d	76.8
Domestic repurposed													
CA200174	1.3	≤ 0.2	20.8	65.4	n.d	4.8	0.9	1.0	5.4	n.d	≤ 0.3	n.d	67.9
CA200175	2.2	1.3	21.7	62.5	n.d	2.9	2.7	0.6	6.1	n.d	n.d	n.d	75.3
CA200176	2.0	1.1	26.8	55.0	n.d	2.2	0.5	1.1	11.3	n.d	n.d	n.d	58.5
CA200177	1.1	0.2	20.4	64.3	n.d	6.1	0.9	0.9	5.3	n.d	n.d	0.7	82.3
CA200179	1.5	0.9	24.4	62.8	n.d	1.9	0.9	0.7	6.9	n.d	n.d	n.d	73.0
Domestic pottery													
CA200180	0.9	0.7	24.7	62.2	0.4	2.1	1.7	0.7	6.5	n.d	n.d	n.d	75.9
CA200181	0.7	0.5	23.9	60.3	0.9	2.6	2.0	0.9	8.3	n.d	n.d	n.d	71.0
CA200182	1.6	0.8	20.2	65.7	0.4	4.2	1.9	0.6	4.5	n.d	n.d	n.d	75.5

**Table 5:** Composition of the ceramic matrix for all samples (all values reported as normalised to 100 wt%)

Purpose-made	Na_2_O	MgO	Al_2_O_3_	SiO_2_	P_2_O_5_	K_2_O	CaO	TiO_2_	MnO	FeO	SnO_2_	PbO	Analytical total
CA200170	n.d	≤ 0.2	36.5	56.1	n.d	4.3	n.d	0.9	n.d	2.0	n.d	n.d	97.3
CA200171	n.d	≤ 0.5	33.7	57.6	≤ 0.5	4.3	≤ 0.6	0.7	n.d	2.0	n.d	n.d	95.1
CA200172	n.d	n.d	33.6	58.7	n.d	4.7	n.d	0.7	n.d	1.9	0.4	n.d	92.0
CA200173	n.d	n.d	41.2	53.5	n.d	3.0	n.d	0.5	n.d	1.9	n.d	n.d	101.3
Furnace wall													
CA200178	n.d	n.d	36.0	53.2	n.d	2.3	n.d	0.7	n.d	7.9	n.d	n.d	83.8
Domestic repurposed													
CA200174	1.3	0.2	30.0	55.5	n.d	4.9	0.7	1.7	n.d	5.8	n.d	n.d	83.3
CA200175	1.3	1.9	29.3	49.7	0.4	3.9	3.0	0.9	≤ 0.2	9.4	n.d	n.d	69.4
CA200176	0.9	1.3	31.6	51.4	n.d	1.9	0.5	0.9	n.d	11.4	n.d	n.d	66.6
CA200177	1.4	0.6	31.0	49.2	0.5	5.0	1.2	1.9	n.d	8.5	n.d	0.8	86.1
CA200179	1.3	1.1	33.1	52.9	n.d	1.7	0.6	0.8	n.d	8.6	n.d	n.d	80.8
Domestic pottery													
CA200180	n.d	1.0	31.7	53.8	0.8	1.6	2.2	0.6	n.d	8.2	n.d	n.d	84.9
CA200181	≤ 0.6	0.7	29.6	55.2	1.1	1.6	2.2	0.9	n.d	8.4	n.d	≤ 0.2	84.2
CA200182	0.7	1.6	27.9	56.2	0.9	1.4	2.8	1.1	n.d	7.4	n.d	n.d	92.5

**Table 6:** Composition of slags (all values reported as normalised to 100 wt%). No slag was found on CA200175

Purpose-made	Na_2_O	MgO	Al_2_O_3_	SiO_2_	P_2_O_5_	K_2_O	CaO	TiO_2_	MnO	FeO	PbO	Analytical total
CA200170	≤ 0.2	0.2	25.0	63.7	n.d	5.9	3.5	0.4	n.d	1.1	n.d	95.6
CA200171	0.5	5.6	16.1	49.1	1.8	4.4	20.7	0.5	n.d	1.5	n.d	103.9
CA200172	0.3	1.7	23.3	55.0	n.d	5.5	12.7	0.5	n.d	1.1	n.d	102.0
CA200173	0.1	8.0	14.3	56.5	0.6	4.9	13.9	0.6	0.2	0.9	n.d	90.5
Furnace wall (vitrification)												
CA200178	0.6	3.2	25.8	53.1	0.9	6.6	4.5	0.7	≤ 0.2	4.4	n.d	96.7
Domestic repurposed												
CA200174	1.2	7.8	16.6	52.5	0.9	5.1	11.8	0.7	n.d	3.4	n.d	100.1
CA200175												
CA200176	1.0	4.1	21.3	44.7	3.4	3.4	14.2	0.8	n.d	7.0	n.d	105.7
CA200177	0.7	1.5	11.4	39.6	1.3	2.5	9.5	0.7	n.d	2.9	29.9	97.4
CA200179	1.5	9.4	12.2	45.3	3.6	2.6	16.1	0.6	0.3	8.6	n.d	96.8

## Results

To allow for easy comparison of the inclusion mineralogy, compositional details, and technical attributes within and between groups (reused sherds, crucibles, and pottery), the results are presented by typology before being combined in the discussion to consider their wider implications for raw material selection, gold processing technology, and organisation of production.

### Purpose-made crucibles

**Fig. 6:**
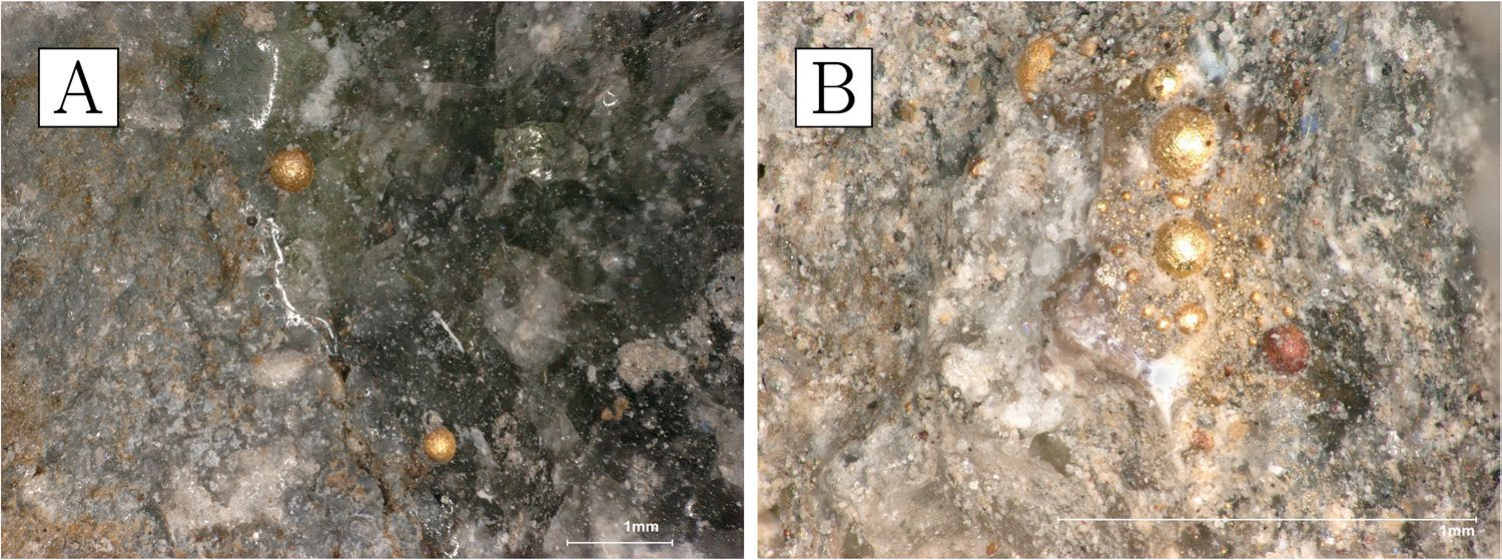
**a** Prills on the inner surface of CA200170 (purpose-made crucible). **b** Prills on the inner surface of CA200173 (purpose-made crucible) (Keyence VHX6000)

The crucibles that were studied are mostly semi-ellipsoid (Fig. 5), with rounded or pointed bases and profiles that taper outwards while remaining relatively closed. Maximum crucible height is around 5 cm, and maximum external rim diameter around 4 cm. They were made of fine-grained, white-firing fabrics, and walls are relatively thick, reaching around 1 cm at the base. They display thin (≤ 200 µm) glassy vitrification layers, ranging in colour from grey to green. In most cases, this vitrification is clearly constrained to the top third of the interior of the crucible (with some occasional spills on the outside)—a clear indication that this oxidised dross formed and floated on the surface of a denser metal bath (Fig. 5). Gold prills, some macroscopic (Fig. 6), are trapped within the slags of all crucibles other than CA200172.

**Fig. 7:**
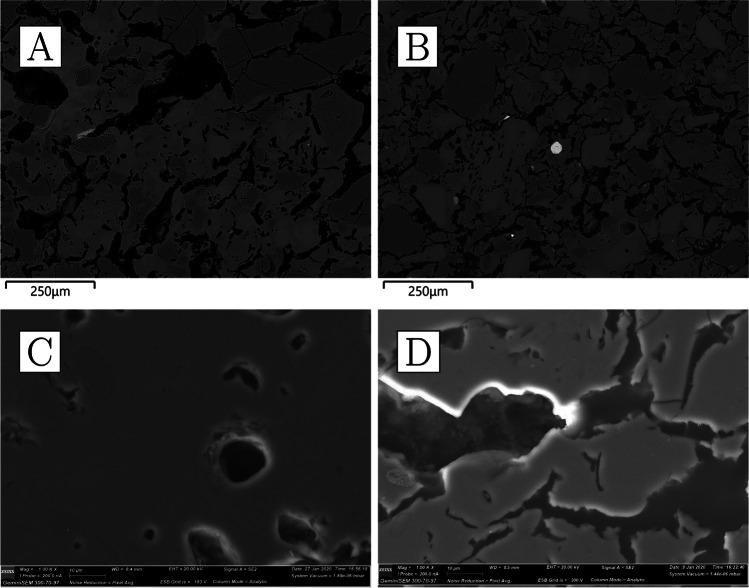
**a** Ceramic fabric of CA200170 (purpose-made crucible); note alignment of porosity alongside direction of forming, darker grey quartz, lighter grey feldspars that are disintegrating to the matrix, and a bright, small, elongated rutile inclusion (× 100, BSE). **b** Ceramic fabric of CA200171; note alignment of porosity, darker grey quartz, lighter grey feldspars that are disintegrating to the matrix, and a bright, angular zircon inclusion (× 100, BSE). **c** Ceramic matrix of CA200170 (purpose-made crucible); note how the matrix appears more vitrified than for CA200171 (× 1000, SE). **d** Ceramic matrix of CA200171 (purpose-made crucible) (× 1000, SE) (Zeiss Gemini SEM 300)

The bulk composition of the ceramics is characterised by very high levels of alumina (22–27%), some potash (~ 4%), and notably low iron oxide (≤ 2%) (Table 4). All other oxides occur in concentrations lower than 1%. Ceramic matrix analysis yielded even higher alumina values (34–41%) (Table 5), as it avoids the dilution effect of quartz grains contributing to the bulk silica content. Mineral inclusions are abundant but rather small, rarely exceeding 300 µm in diameter. Most common are quartz and potassium feldspars (the latter sometimes with BaO above detection limits), with rarer but consistent presence of Mn-bearing ilmenite and rutile, as well as zircon (the latter, bearing Y, Nb, Hf, and/or Th) and, in CA200172, chromite and U-rich thorite. In addition, very small, potentially diagnostic, monazite grains of various compositions were detected in three of the samples, with xenotime detected in the fourth (Table S2.2). The ceramic matrix is only moderately vitrified and some of the porosity is angular and retains alignment along the direction of forming, as expected from the high refractoriness of the alumina-rich clays employed; however, larger mineral inclusions, particularly feldspars, are often amorphous and appear disintegrating/melting into the ceramic matrix, as a result of high-temperature impact. There is no obvious gradient in the heat impact across the ceramic, making it plausible that the crucibles were heated from the outside. This proposition is also consistent with the relatively closed shape of the vessels and the presence of vitrification patches and other clear traces of heat impact on the outside. CA200170 and CA200172 appear more vitrified than CA200171 and CA200173 (Fig. 7), and these differences could not be explained by consistent compositional differences.

**Fig. 8:**
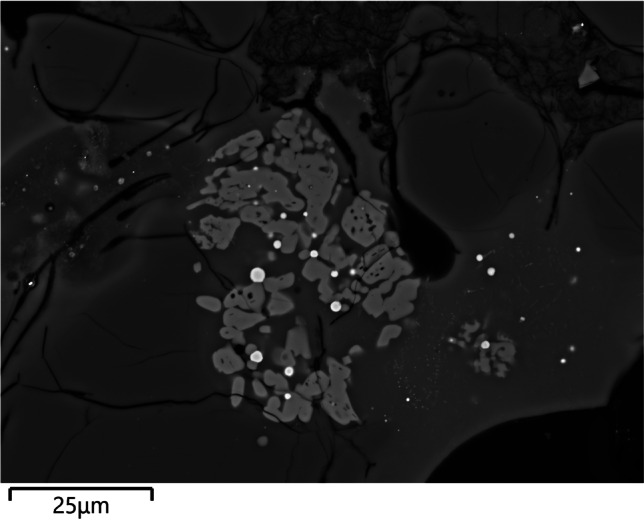
Metallic iron reducing from ilmenite in CA200171 (purpose-made crucible) (BSE) (Zeiss Gemini SEM 300)

**Fig. 9:**
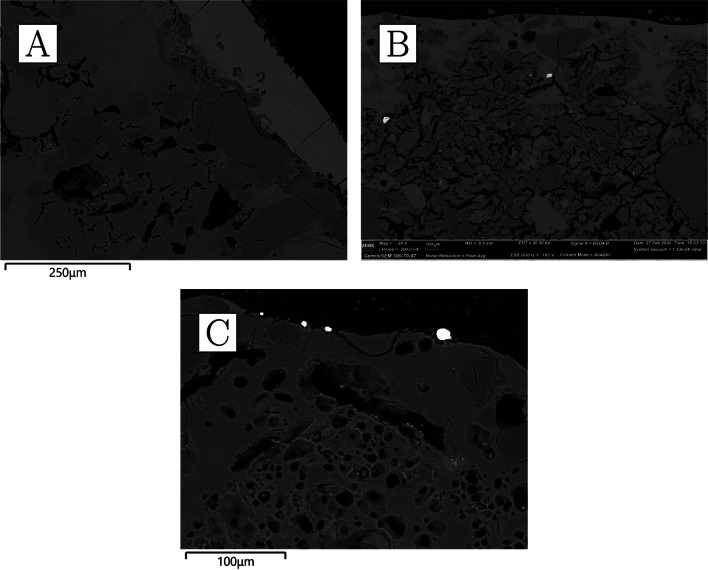
**a** Slagging on CA200171 (purpose-made crucible); note the clear boundary between the slag and the ceramic body (BSE). **b** Slagging on CA200173 (purpose-made crucible); note the gradual boundary between the ceramic body and slagging, as is typical of this group of samples (BSE). **c** Metal prills on the surface of CA200170 (purpose-made crucible) (BSE) (Zeiss Gemini SEM 300)

Clusters of very small droplets of metallic iron are present in all of the samples in this group, clearly exsolving out of ilmenite inclusions as a result of the high temperature and reducing environment (Fig. 8). With the exception of CA200171, the boundary between the ceramic and the “slag” is gradual, indicating that this layer is largely formed as a result of ceramic surface vitrification (Fig. 9). Compositionally, the glassy layer is similar to the ceramic, but enriched in lime and/or magnesia. This layer often contains mineral inclusions clearly absorbed from the ceramic fabric, in addition to occasional, small P-rich iron droplets, and more numerous metallic prills. The latter were shown to be argentiferous gold, with notable variation within and between samples: silver levels typically range from 2 to 12%, and copper is below detection (estimated at ~ 0.1%), except for one prill in CA200171 with 1% copper (Table S2.5). A few metal globules showed lower silver values, but this is likely a result of local concentration effects or sampling bias, given that these were invariably the smallest ones (≤ 8 µm). Finally, a single copper prill was found within the ceramic fabric of CA200171 (Table S2.5).

We also report in this group sample CA200178, which was harder to classify morphologically as its external (“cold”) surface was more irregular, and it did not contain entrapped gold prills. We interpret this as the vitrified lining of a furnace wall. It is compositionally and mineralogically similar to the crucibles, although with slightly higher iron levels.

### Re-purposed domestic ceramics

**Fig. 10:**
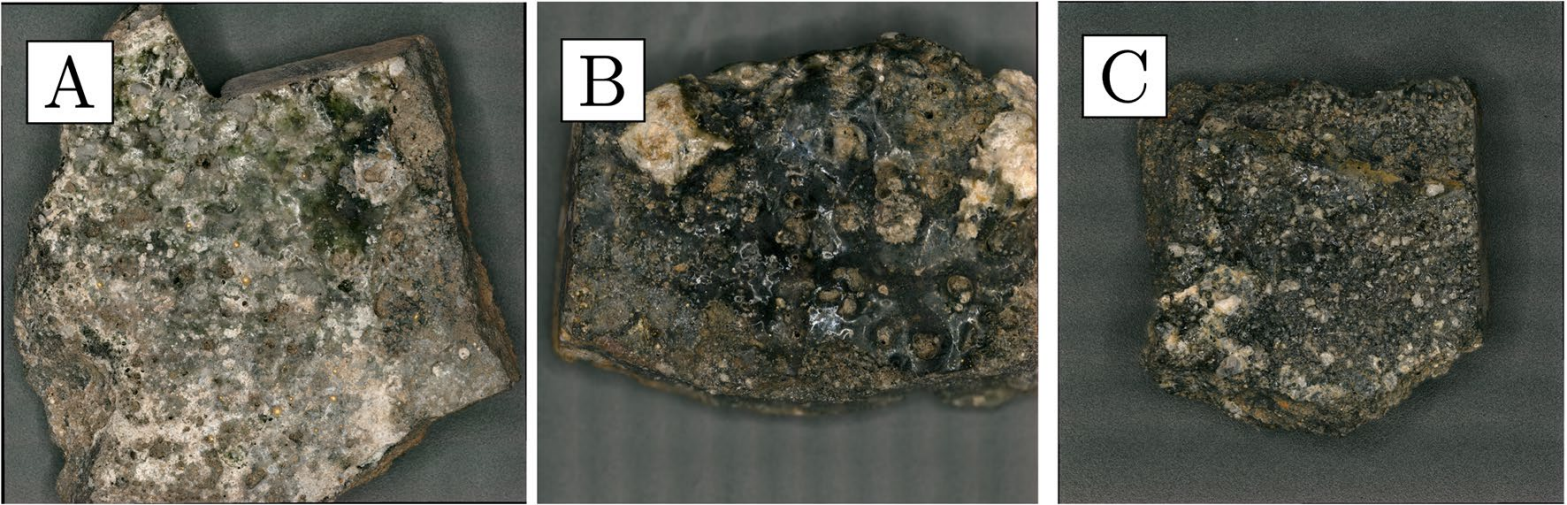
Samples of repurposed domestic ceramics. **a** CA200174; note presence of several macroscopic gold prills and varying degrees of slagging across the inner sample surface, with vitrification extending into broken cross-section on the left. **b** CA200176; note the presence of gold prills and extensive slagging across the whole inner sample surface and extending into broken cross-section at the top. **c** CA200179; note the presence of more localised slagging on the inner sample surface (Keyence VHX6000)

Repurposed domestic sherds are broadly similar to domestic pottery used during different occupation periods at Great Zimbabwe. They have the same surface treatment, fabric texture, and curvature; the only distinguishing feature being the presence of layers of metal processing residues that are absent in domestic pottery. All of the sherds in this group are flat or only slightly curved, indicating that they are likely to come from relatively large vessels. Unfortunately, due to high rates of fragmentation, the size of the vessels could not be estimated. The colour of the fired ceramics varies from brownish to reddish, with many of the samples having darker, reduced areas towards the inner surface (Fig. 10). The ceramics belonging to this group are more vitrified on the inside, indicating they were heated from above/within rather than sitting on a fire. The vitrification is either localised (Fig. 10c) or more extensive and aggressive across the whole sample surface (Fig. 10b).

**Fig. 11:**
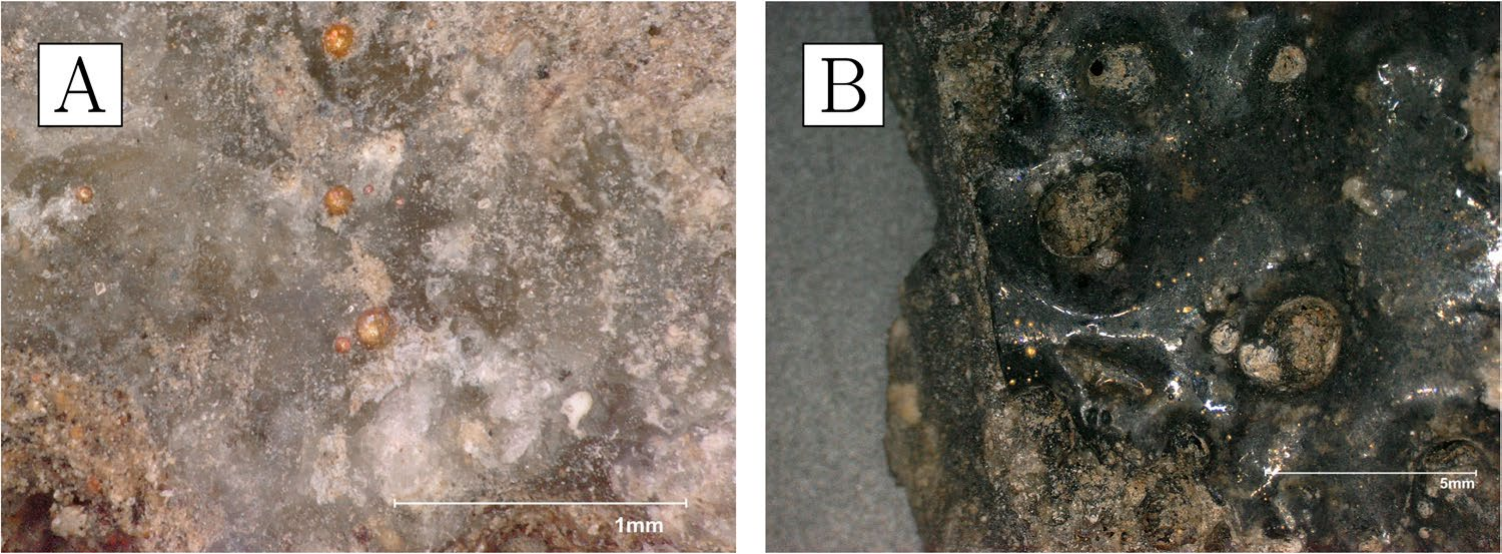
**a** Prills on the inner surface of CA200174 (repurposed ceramic). **b** Prills on the inner surface of CA200176 (repurposed ceramic); note the frequent presence of gold prills on slagging (Keyence VHX6000)

Gold prills can be seen on the surfaces of all the samples, sometimes occurring with relatively large quartz grains. The amount and size of these prills vary, with a higher frequency in samples with more slagging (Fig. 11).

The texture of these ceramics is generally coarser than that of the crucibles, and they have higher iron oxide (bulk 5–12%) and sometimes marginally lower alumina (20–27%), with alkali oxides and lime above detection limits in all cases (Table 4). Samples CA200174 and CA200177 are finer grained, and their matrix composition is slightly higher in titania (Table 5). Typical inclusions include quartz, alkali feldspars, and other alumina silicates bearing alkali and iron oxides, ilmenite, rutile, and other iron and titanium-rich minerals, and zircon. The potassium feldspars tend to be larger in size than the other feldspars. Very small grains of thorite were detected in CA200176, and chromite in CA200179 (Table S2.1).

**Fig. 12:**
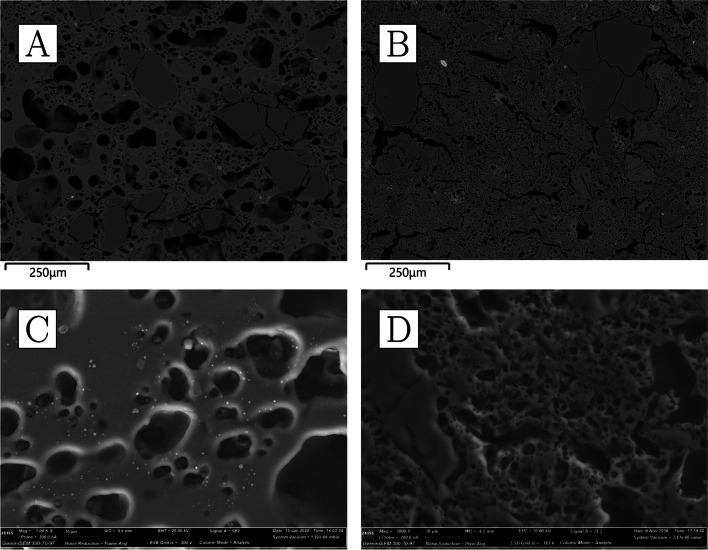
**a** Ceramic fabric of CA200174 (repurposed ceramic); note the presence of thermally cracked quartz inclusions and heavily bloated ceramic body that contains disintegrated feldspars (× 100, BSE). **b** Ceramic fabric of CA200176 (repurposed ceramic); note the thermally cracked quartz, lighter grey feldspars that have retained their shape, as well as bloated ceramic body (× 100, BSE). **c** Ceramic matrix of CA200174 (repurposed ceramic); note roundedness and size of porosity (× 1000, SE). **d** Ceramic matrix of CA200176; note roundedness of porosity, but reduced size in comparison to CA200174 (× 1000, SE) (Zeiss Gemini SEM 300)

**Fig. 13:**
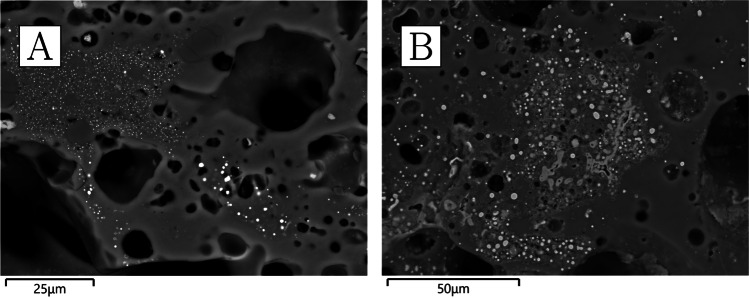
Clusters of metallic iron prills in the fabric of the two most heavily bloated repurposed domestic ceramics, resulting from the reduction of iron-rich minerals. **a** CA200174 (BSE); **b** CA200179 (BSE) (Zeiss Gemini SEM 300)

Reused sherds display vitrified/bloated ceramic matrices and thermally cracked quartz inclusions (Fig. 12). Feldspars seem to have mostly retained their shape, with the exception of the two samples with the greatest degrees of ceramic bloating, CA200174 and CA200179, where these minerals are more thermally altered. These two samples are also the only ones consistently showing reduced metallic iron in iron-rich minerals (Fig. 13).

**Fig. 14:**
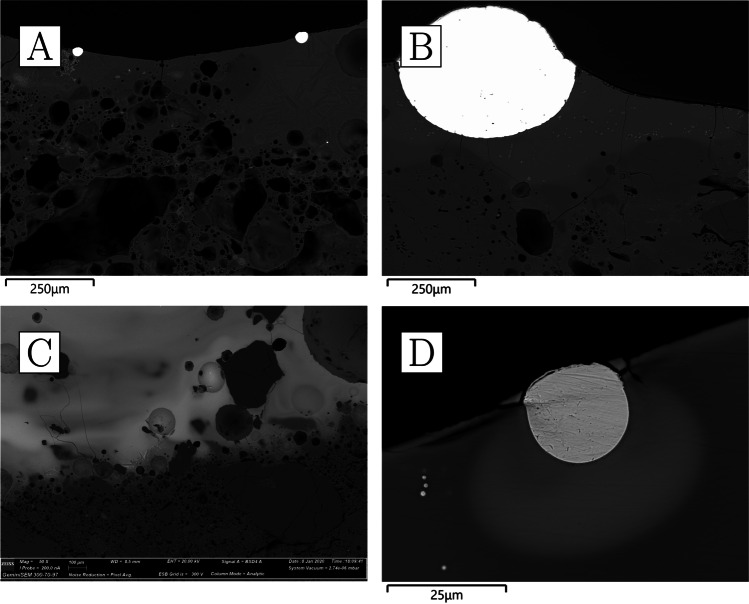
**a** Slagging on CA200176 (repurposed ceramic); note gradual boundary between the slag and the ceramic body, and the metal prills on the surface (BSE). **b** Prill on the surface of CA200179 (repurposed ceramic) (BSE). **c** Interphase between the slag and ceramic body in CA200177 (repurposed ceramic); note the distinct boundary between the two, which is unusual for this group. **d** Prill with halo in lead glass on CA200177 (repurposed ceramic) (BSE) (Zeiss Gemini SEM 300)

With the exception of CA200177, the interface between the ceramic body and the slag tends to be gradual (Fig. 14). Compositionally, the vitrified layers have increased lime, magnesia, and, to a lesser extent, phosphate (Table 6). Although quartz inclusions are often present, it remains difficult to determine whether these represent part of the metallurgical charge or minerals absorbed from the ceramic body. The majority of the metal prills analysed were found to be argentiferous gold (Table S2.5), although in some cases no silver or very low levels of silver were detected, particularly in the smallest ones, with those in CA200174 and CA200176 yielding Ag ≤ 1.7%, and those in CA200179 in between 9 and 12% Ag. A tiny (1 µm) globule of nominally pure silver was found in CA200179.

CA200177 stands out in that it has a thicker and more distinct slagged layer, with ~ 30% PbO. The lead oxide enrichment is particularly noticeable in the halo around one of the larger gold prills, where the slag was also enriched in bismuth oxide (54% PbO, 10% Bi_2_O_3_) (Fig. 14d). Unlike the metal in the other ceramic residues, the globules here showed variable levels of copper as well as arsenic, bismuth, and thorium (Table S3.8.6).

### Domestic pottery

**Fig. 15:**
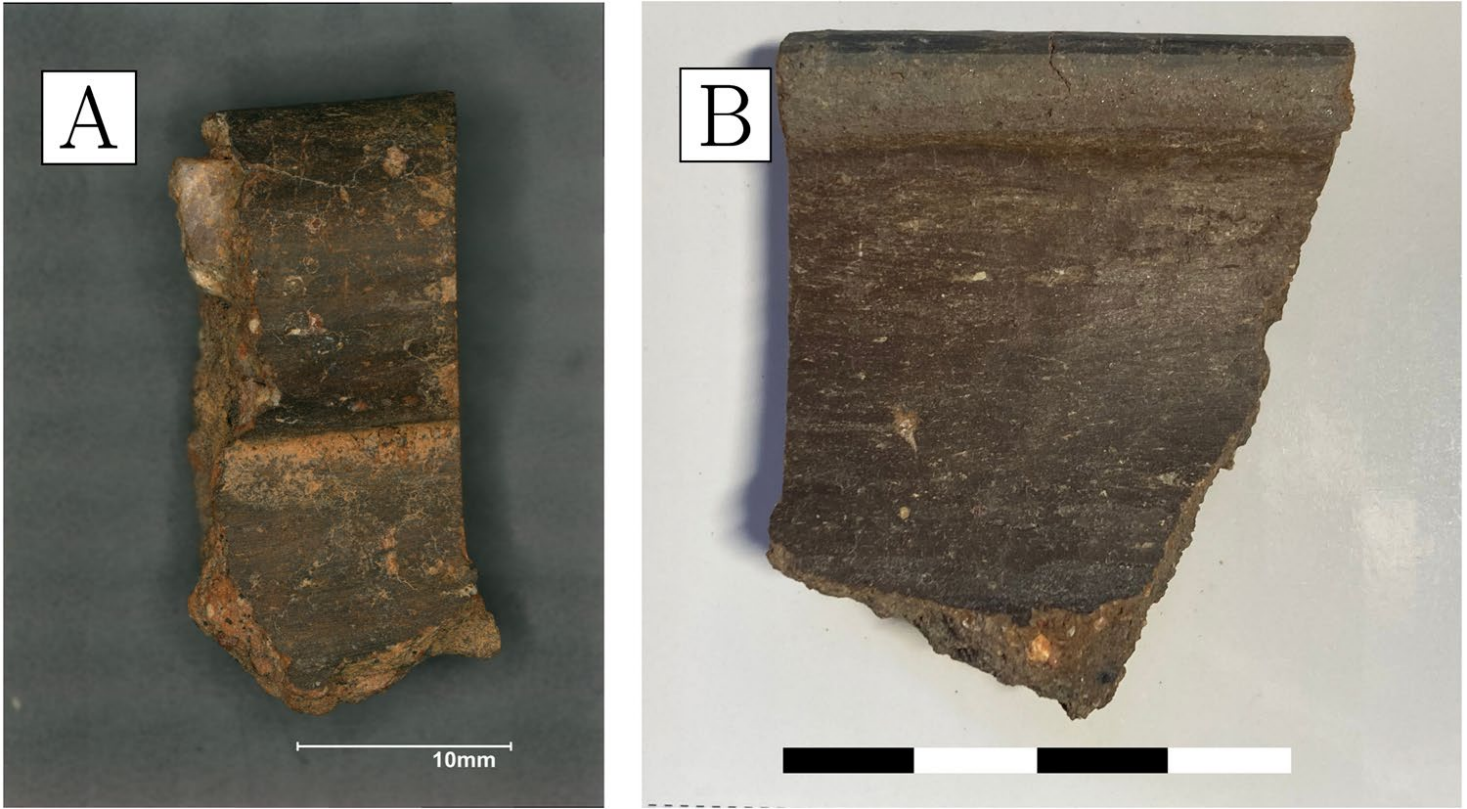
**a** Sample CA200180 (domestic pottery); note reddish colour in cross-section and darker burnishing on the surface (Keyence VHX6000). **b** Sample CA200182; note reddish-brownish colour in cross-section, with darker burnishing on the surface

**Fig. 16:**
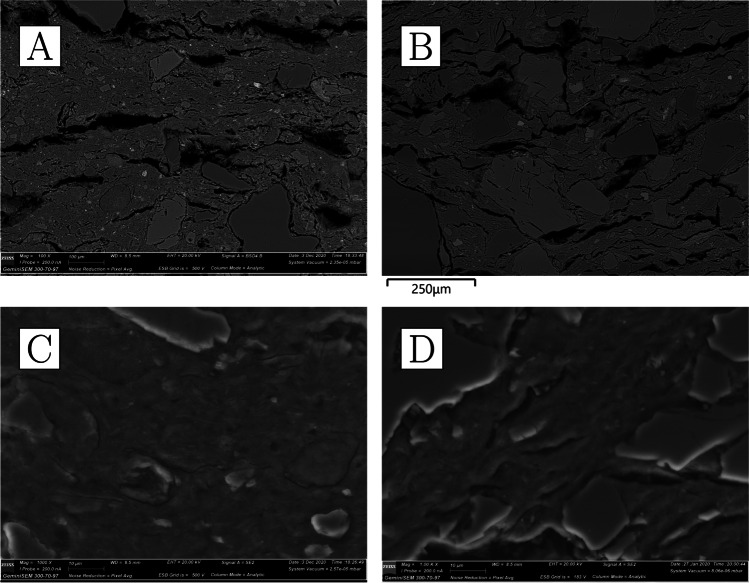
**a** Ceramic fabric of CA200180 (domestic pottery); note presence of darker-grey quartz and lighter-grey feldspars (× 100, BSE). **b** Ceramic fabric of CA200182 (domestic pottery); note frequent presence of large quartz inclusions and lighter-grey feldspars (× 100, BSE). **c** Ceramic matrix of CA200180 (domestic pottery); note presence of individual clay platelets (× 1000, SE). **d** Ceramic matrix of CA200182 (domestic pottery); note presence of individual clay platelets (× 1000, SE) (Zeiss Gemini SEM 300)

We analysed three sherds of domestic pottery to compare them to the metallurgical ceramics. The samples are reddish in colour, with polishing or a darker burnishing on their outer surfaces (Fig. 15). All three samples appear to have moderately coarse fabrics, with larger quartz inclusions more frequent in CA200182 (Fig. 16a, b) and correspondingly slightly higher bulk silica levels (Table 4). In general, their composition is similar to that of the sherds interpreted as repurposed ceramics, albeit with slightly lower soda and lower potash (as well as marginally higher phosphate, but this may be post-depositional in these porous fabrics; cf. Freestone (2001) on post-depositional alteration of ceramics and glass). Typical mineral inclusions comprise quartz, alkali feldspars, iron-rich clay pellets, and minerals, ilmenite, rutile, and zircon (Table S2.4); like in the repurposed ceramic sherds and unlike the purpose-made crucibles, no monazite was found. An examination of their ceramic matrices shows absence of vitrification, with individual clay platelets still visible in all samples, and denoting lower firing temperatures (Fig. 16c, d).

## Discussion: gold processing and use at Great Zimbabwe

### Metallurgical ceramics and processes

**Fig. 17:**
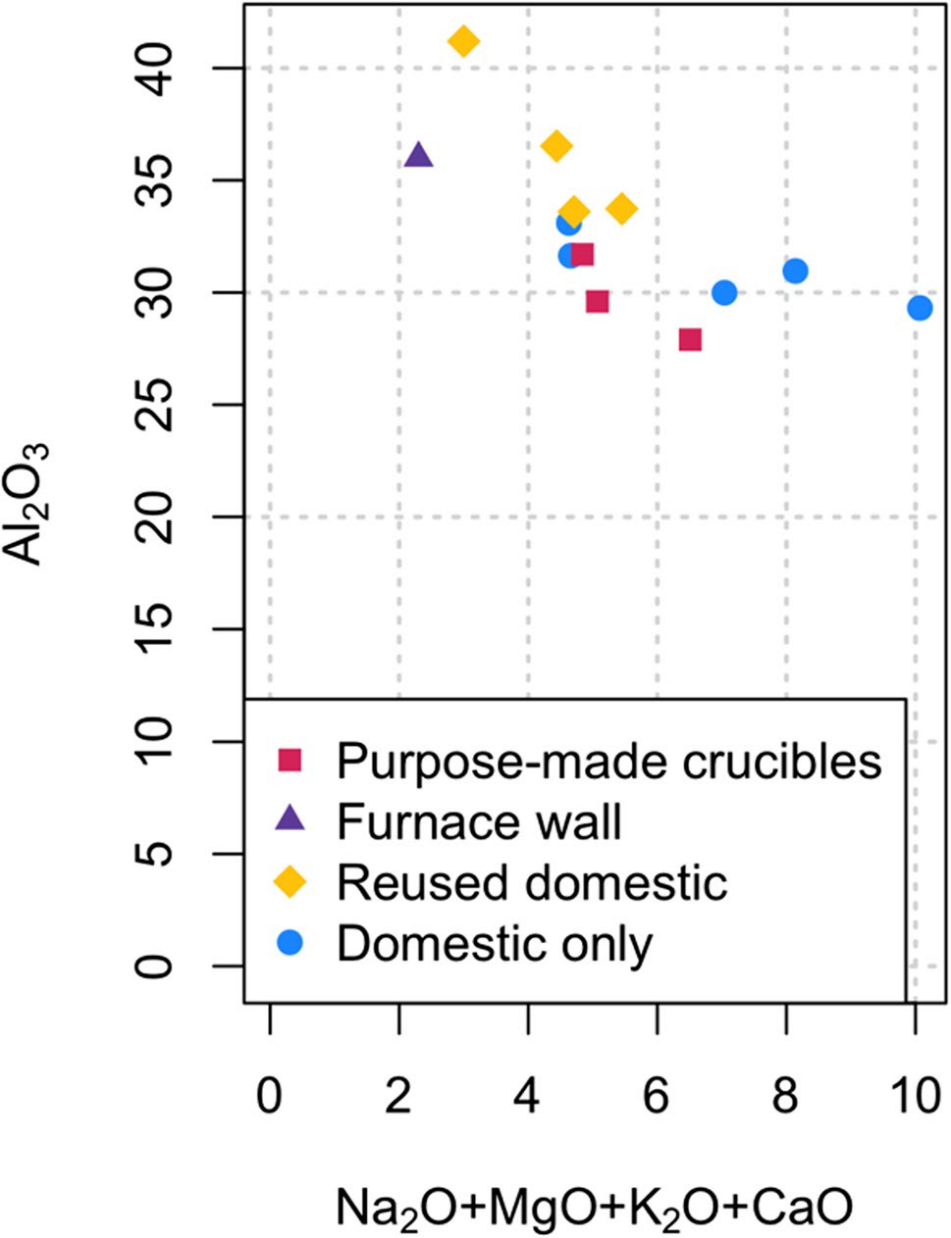
The ratio of alumina to alkalis in the ceramic matrices (graph created in R v.4.0.2 (R Core Team 2020))

The macroscopic attributes, inclusion mineralogy, and chemical characterisation of vessel fabrics and attached slag including metal prills revealed vital clues regarding knowledge of properties and performance of clays, and their use in making containers for processing gold. The local clays around Great Zimbabwe are very rich in kaolin, a pedological characteristic which resulted in them being classified into the orthoferralitic group (Purves 1976; Nyamapfene 1983; see also Ekosse 2010). All the ceramics analysed are notably high in alumina and dominated by felsic minerals and other inclusions that are known to occur in the granitic environment around Great Zimbabwe. Thus, while identifying the specific clay sources employed is beyond of the scope of this paper, it seems likely that both domestic and metallurgical ceramics were produced locally. The similarity between furnace lining CA200178 and other fabrics would lend support to this proposition. At the same time, small variations between fabric groups suggest that more than one source may have been employed. Of particular note is the fact that purpose-made crucibles are consistently higher in alumina and lower in iron and alkali oxides, which would confer them with higher refractoriness (Fig. 17) (Freestone and Tite 1986; Martinón-Torres and Rehren 2009, 2014). As such, it is tempting to propose that whiter clays were recognised and selected for when producing metallurgical vessels. With melting temperatures exceeding 1000 °C, gold and copper would be the most demanding metals in terms of the refractory requirements for the associated ceramics.

Of particular interest, however, is the occurrence of both purpose-made crucibles and repurposed domestic ceramics, co-existing spatially and chronologically, and suggestive of continuity and change from the first millennium CE (Bisson 2000; Bandama et al. 2016). Recycling pottery from cooking and everyday storage into metallurgy attests to improvisation and cross-craft use of vessels in quotidian and technical practices (Chirikure et al. 2015). This practice was very common in copper-based metallurgy in ancient southern and central Africa and goes back to around 400 CE (Swan 1994; Bisson 2000; Miller 2010; Chirikure et al. 2015; Bandama et al. 2016). Crucibles and reused pottery were in use at Great Zimbabwe from around 1000 CE onwards (Robinson 1961; Bandama et al. 2016, 2017) and this continued well into the sixteenth century, as shown by the finds from the Upper Ridge and the Fireguard Midden including Khami-type glass beads. But what exactly were these ceramics used for, and were both purpose-made crucibles and repurposed ceramics used for the same metallurgical process?

The metal prills within the vitrified residues leave little doubt that the main purpose of all of these ceramics was the processing of gold. With moderate silver levels and generally very low or absent copper, the composition of the metal is consistent with that of natural, unalloyed gold. Occasional traces of copper and arsenic would correspond to the odd chalcopyrite and/or arsenopyrite or other minerals mixed as impurities with the natural gold. The ore sources in Zimbabwe’s greenstone belts and granitoid geological backgrounds are polymetallic (Naden et al. 1994; Mugumbate et al. 1999, 2000; Klemm and Kräutner 2000), and hence this should not be surprising. The slagged layers are rather similar in both ceramic types, although with a tendency for repurposed ceramics to show thicker vitrification layers with larger quartz grains. The chemical enrichment in lime and/or magnesia (and in some cases phosphate) might simply derive from charcoal ashes, but magnesia:lime ratios are sometimes too high for wood ashes (or indeed for a glass flux, cf. Rehren and Nixon 2014) and may therefore denote the former presence of Mg-rich gangue. For example, Mg-bearing actinolite occurs with gold ores at Freda Rebecca in Bindura (Klemm and Kräutner 2000), approximately 400 km north of Great Zimbabwe. The evidence would therefore point to the processing of fresh geological gold as opposed to other processes such as alloying or recycling.

Reused sherd CA200177 has a much thicker residue, and this is unusually enriched in PbO, in addition to having prills that are variously enriched in Bi, Ag, Cu, As, in addition to the argentiferous gold. There are mines such as Athens Mine in Mvuma, around 130 km north of Great Zimbabwe, that contain precisely the suites of minerals that would lead to this peculiar assortment (Naden et al. 1994). Furthermore, this mixture of minerals is associated with polymetallic deposits associated with mafic and ultramafic deposits such as the Masvingo Greenstone Belt, Mashava Ultra Mafic Complex, and the Mberengwa Greenstones that would have been exploited by Great Zimbabwe. While it would be tempting to see here the separate addition of lead to extract noble metals by cupellation, at present it seems more parsimonious to regard this as a geological coincidence that attests to the diversity of gold sources processed at the site, which probably included hard-rock hydrothermal sources as well as placers.

On the whole, it is therefore difficult to differentiate both ceramic types based on the chemistry of the associated residues, but their overall morphology may furnish relevant information (Bayley and Rehren 2007; Martinón-Torres and Rehren 2014). While some of the repurposed sherds show fresh fractures that postdate use, the vitrification and slag smear often cover the fractured edges, indicating that the sherds were used for metallurgy after fracture. Their shape would therefore be that of relatively small, open dishes, heated from above (as demonstrated by the vitrification gradient), and conducive to primarily oxidising atmospheres. This would make them similar to the so-called scorifiers used in Europe since the Middle Ages for assaying and refining noble metals (Martinón-Torres and Rehren 2005). Conversely, the crucibles are more closed in profile and heated from the outside; they would be able to hold larger volumes of metal (see below) and probably for longer (as indicated by the typically more distorted minerals within the fabric). Their pointed shape would facilitate the collection of gold under reducing conditions, and its separation from any remaining impurities that would form a dross layer floating on top. With this in mind, we may propose that repurposed ceramics would have been better suited for the initial refining of gold nuggets or dust, while crucibles would have been employed for the consolidation of this refined gold into larger masses that could be cast into ingot moulds or other shapes. This is not to imply that both steps would always and invariably take place in succession: as documented in European treatises, it is likely that metallurgists adjusted their operations to their initial assessment of the raw materials and tools at hand, and there is no need to assume that each reaction was conceived of as a distinct category. In a similar vein, recent work on prehistoric bronze metallurgy in Europe has pointed to very blurred boundaries between operations that today we regard as separate, such as smelting, melting, or alloying (Farci et al. 2017; Montes-Landa et al. 2020). In this light, the occurrence of both crucibles and repurposed ceramics would further highlight the versatility and adaptability of those who processed gold at Great Zimbabwe.

Hall and Neal (1902) mention the recovery of gold cakes, making it possible that some of the gold was consolidated into an ingot for later use. The likely diversity of use for the metallurgical ceramics is also complemented by the recovery of moulds used to produce small bars of gold at Great Zimbabwe (Bandama et al. 2017). While repurposed ceramics show cross-craft overlaps in container use, crucibles would be more fitting for the production of standardised shapes and volumes.

**Fig. 18:**
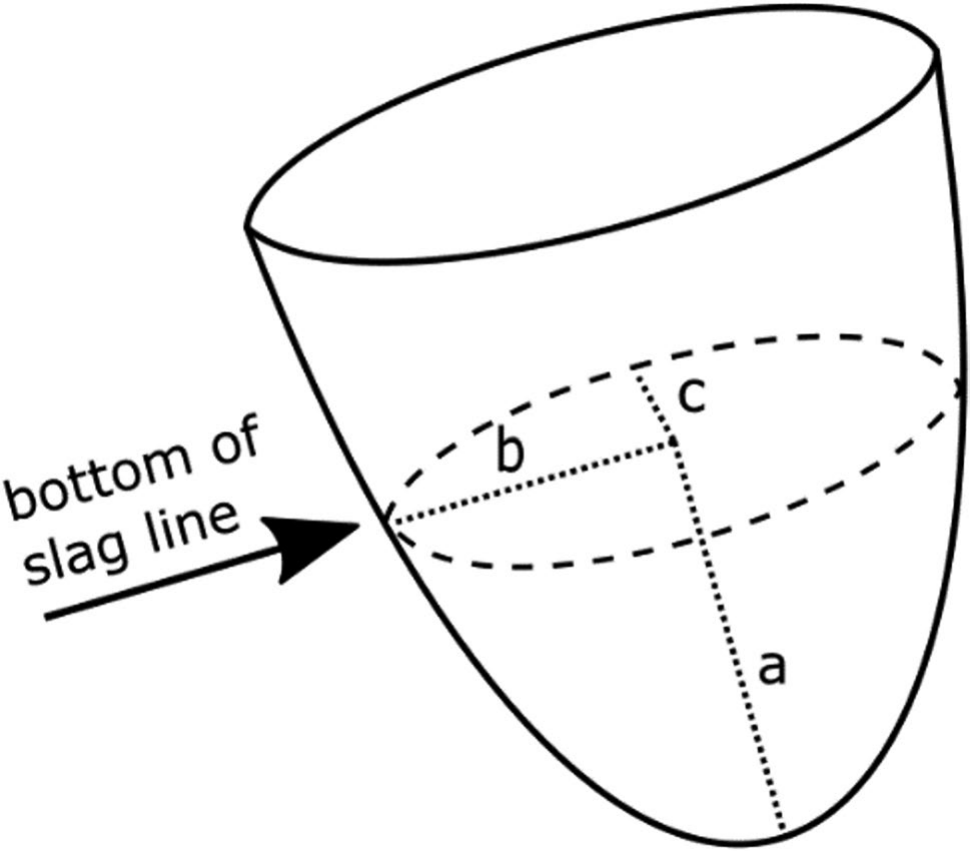
Measurements used for the calculation of semi-ellipsoid volume (graph created in Inkscape (Inkscape Project 2020))

The complete nature of the crucibles from the Upper Ridge area made it possible to estimate the volume of metal that solidified below the slag line. This was based on the assumption that molten metal solidified below the slag layer, creating an ingot that inherited the semi-ellipsoid shape of the crucibles. Measurements were taken of the diameters and height (depth) of the crucibles below the slag line (*n* = 24). This provided an opportunity to estimate the average volume of ingots that were produced in the crucibles using the following formula for estimating the volume (*v*) of a semi-ellipsoid:

$$v= \frac{2}{3}*\pi *a*b*c$$

with *a*, *b*, and *c* referring to the bisected axes as indicated on Fig. 18; the average measurements of which were as follows:

$$a=3 \mathrm{cm}, b=1.8 \mathrm{cm}, c=1.5 \mathrm{cm}$$

The calculation [2/3*3.142*3*1.8*1.5] returned an estimated average volume of 16.96 cm^3^ of gold, translating into 327 g per crucible ingot (density of gold is 19.3 g/cm^3^). A hundred of these crucibles would have produced nearly 33 kg of gold. This shows that the Upper Ridge was a site of intensive gold production, some of which was used on site. Other settlements such as the Camp Ruins produced intensive evidence of gold working, most of which was not adequately recorded by antiquarians (Bandama et al. 2017). Only reused sherds were studied from the Camp Ruins and the Barrier Hut unwalled settlements west of the Car Park (Bandama et al. 2016). An unknown quantity of custom-made crucibles, moulds, and reused sherds was recovered from the Hill summit and the southern slopes as well as from the Great Enclosure (Robinson 1961; Chirikure 2020a). It however appears, based on the evidence from the Upper Ridge, that the custom-made crucibles were used for producing standardised shapes and ingots of uniform weight. Therefore, apart from melting, the crucibles were also used for measurement—to produce ingots of standard shape, weight, and value. If that was the case, then the presence of moulds for producing gold bars and the use of different crucible types in other areas of Great Zimbabwe (e.g., the Hill and Camp Ruins) shows, within limitations, the diversity of the forms in which gold was cast, stored, and circulated regionally and with Indian Ocean networks.

### Great Zimbabwe gold, locally and globally

**Fig. 19:**
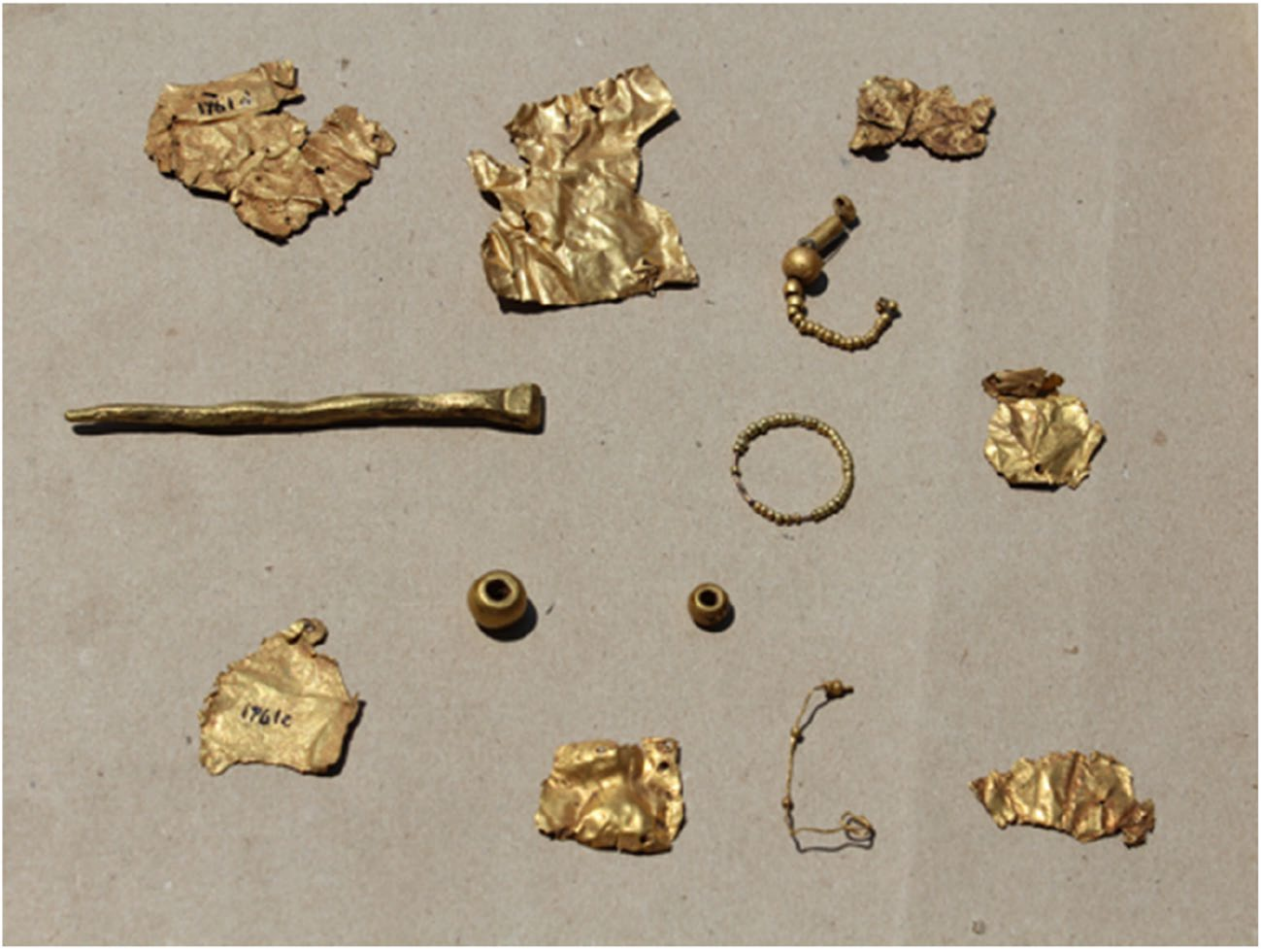
Gold objects on display in the Great Zimbabwe Conservation Centre

Indian Ocean-centric narratives on Great Zimbabwe have traditionally, and incorrectly, emphasised the significance of gold trade in bringing new wealth that promoted the fortunes of elites (Huffman 1972, 2007). The impression is that gold was rarely consumed within Great Zimbabwe, and when it was used, it was for the elites. A view of globalisation that is externally focussed under values  local agency (Pikirayi 2017). What is required is to assess the nature and impact of global interactions from the inside looking out to learn more about local agency in shaping global histories (Chirikure 2020a). Although some of the evidence was destroyed by antiquarians in the late nineteenth and early twentieth centuries (Hall and Neal 1902; Swan 1994), piecing together all the available indicators shows considerable evidence of working and consumption of gold within Great Zimbabwe’s settlements and related places. Available evidence shows that crucibles and gold were recovered from eleventh to twelfth century contexts on the Hill and its southern slopes, from the thirteenth to seventeenth century contexts in the valley and adjacent areas, and also from unwalled settlements on the western side towards Great Zimbabwe Hotel. Crucibles, reused sherds, and finished objects were recovered in the Great Enclosure (Summers 1961), the Valley Enclosures (Caton-Thompson 1931; Collett et al. 1992), the Camp Ruins (Willoughby 1893; Bandama et al. 2017), the Upper Ridge (this study), the hill slopes (Chirikure 2020a), and on the hill summit (Robinson 1961; Bandama et al. 2017). This suggests many hands were involved, with distributed production taking place in various settlements making up the site. While most of the finished gold objects were sold by prospectors associated with the Ancient Ruins Company and private fortune seekers (Hall and Neal 1902), some objects are on display in the Great Zimbabwe Site Museum (Fig. 19).

The gold was worked hot and cold to produce ornaments such as beads and bangles. It was also hammered into thin sheets that were attached to posts inside houses, producing bands of designs as suggested by rivet-perforated thin strips of gold (see Swan 1994; Chirikure 2020a). This explains why antiquarians recovered so much gold from cleaning floors at Zimbabwe-type settlements such as Mundi, Khami, Danamombe, and other places (Hall and Neal 1902; Swan 1994; Miller 2002). Clearly, Great Zimbabwe’s settlements consumed gold, like they did iron, copper, and other metal alloys (Bandama et al. 2017; Mtetwa 2017). Therefore, Great Zimbabwe was not just a conduit for transferring gold to the Indian Ocean; it was also a consumer of gold. According to Oddy (1984) and Miller (2002), the techniques for working gold are similar to those invested in working copper and bronze. Equipment for wire drawing was recovered in the Great Enclosure (Summers 1961) and other places such as Mtuzu Hill. It seems likely that households within Great Zimbabwe’s multiple settlements were multi-crafting; working gold, copper, and its alloys—including bronze—; and spinning cotton fibre, among other activities. This resonates with observations made in different complex societies elsewhere, where multi-crafting in homesteads was a common way of organising production (see for example Shimada 2007). Furthermore, the widespread occurrence of evidence for metalworking, spinning fibres (as shown by presence of spindle whorls), and other crafts in Great Zimbabwe suggests that production was not based on specialised settlements on the site. Rather, it is possible that the specialisation was community based, and that it was staggered and scheduled according to seasons, but practised within the same multi-crafting homesteads.

Attempts to match the composition of gold prills in the studied samples with ore geochemistry and published gold geochemical data raise, within limitations, some very interesting points regarding the possible source of Great Zimbabwe’s gold. As mentioned earlier, the presence of arsenic, lead, and bismuth, particularly in slag in CA200177, suggests that gold dust was melted on site. Most ore bodies within 20 to 200 km from Great Zimbabwe, especially those in greenstones and granitoid backgrounds, are associated with these accessory elements (Naden et al. 1994). The absence of these elements in some of the gold suggests that alluvial gold would have been exploited, although we cannot rule out the exploitation of primary deposits. While detailed provenance studies are required, existing compositional studies revealed that Great Zimbabwe’s gold is argentiferous (Grigorova et al. 1998; Bandama et al. 2017). However, there is a great deal of variation in the levels of silver, in both prills found in crucible slags and finished objects, suggesting the use of multiple ore sources (Netshitungulwana 2011). Great Zimbabwe is surrounded by greenstone and granitoid gold hosting deposits, some of which were exploited in the past (Mennell 1934; Summers 1969). Rivers such as Tokwe, about 20 km to the southwest, are known sources of alluvial gold (Phimister 1976). Nevertheless, most gold sources that were worked are within 100 km of the site, a distance easily accessible even on foot (Chirikure 2020b). The gold was part of a diverse localised and regional circulation network involving other commodities such as grain, cattle, copper, ivory, and other resources. This raises the potential for mixing metal from multiple sources, including the nearby and the distant.

**Fig. 20:**
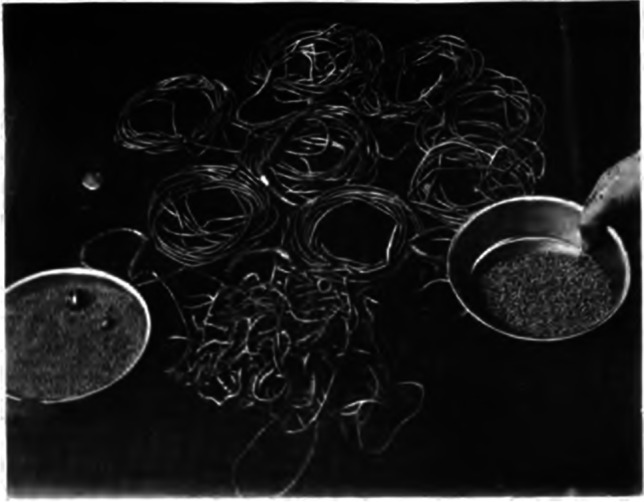
Photograph of some of the gold recovered at Mundie, a relatively small *dzimbahwe* located in Mberengwa by Hall and Neal (1902: 93). Copyright: Hall and Neal (1902)

Gold objects similar to those recovered from Great Zimbabwe have been found at hundreds of related places (*madzimbahwe*) scattered over the territory falling between the Zambezi and Limpopo rivers. Hall and Neal (1902), who harvested gold from some of the sites, provide some estimates of gold objects and ingots recovered by prospectors who excavated house floors and processed the debris for gold during the late nineteenth and early twentieth centuries. They report that Neal and Johnson looted gold objects weighing 500 oz (14.2 kg) from archaeological sites, Burnham got 600 oz (17 kg), while Johnson, Neal, and Leech found 208 oz (5.9 kg), with other prospectors finding at least 60 oz (1.7 kg) (Hall and Neal 1902, pp. 91–93). Overall, they estimated that large quantities of solid gold ornaments and gold cake weighing more than 2000 oz (57 kg) were recovered from the drystone walled sites located in southwestern Zimbabwe alone (Hall and Neal 1902). Some of the burials from Zimbabwe-type settlements such as Danamombe and Mundie had numerous gold objects (Fig. 20). One burial reported by Hall and Neal (1902) was associated with 762 oz (21 kg) of gold, which makes it the highest known quantity found in association with individual skeletons in southern Africa. Clearly, these significant amounts are yet to be factored into explorations of the role of gold within southern African communities, beyond the Indian Ocean export-oriented narrative.

Outside the boundaries of what is now Zimbabwe, gold objects and infrastructure for gold processing such as repurposed sherds (used as crucibles to melt gold) were recovered at Thulamela in the northern Kruger National Park, South Africa, while gold objects were recovered at Bosutswe (Botswana) and Mapungubwe (South Africa) (Grigorova et al. 1998; Miller et al. 2001). The burials on top of Mapungubwe Hill yielded a significant amount of gold, estimated to weigh 2.3 kg (75 oz) in total. One female burial was associated with approximately 2 kg of gold made up of over 100 gold coiled-wire bangles around her legs, pieces of gold plating possibly from a head rest, and 12,000 gold beads around her neck, as well as a very large number of glass beads (Miller et al. 2001). However, Mapungubwe is yet to yield evidence of gold processing in the form of crucibles or reused sherds (Miller 2002). On a regional scale, it is clear that some individuals were buried with huge amounts of gold, copper (e.g. see Chirikure 2015 for Danamombe), and other metals. The quantities reported by Hall and Neal (1902) were not exaggerated. Swan (1994) and Miller (2002, p. 1083) also provide corroborative evidence from other sources of the time indicating that large amounts of gold fell into the hands of antiquarians. This precludes us from a fuller understanding of the place of gold in local consumption tastes. Regardless of the caveat, geochemical studies performed on over one hundred gold objects from Thulamela (South Africa), Mapungubwe (South Africa), Bosutswe (Botswana), and Great Zimbabwe revealed different geological sources (Miller et al. 2001). While Thulamela and Mapungubwe gold likely derived from a related source, Bosutswe and Great Zimbabwe had different sources that were distinct from each other (Netshitungulwana 2011). This suggests the presence of diverse regional economies with different capillary networks that were somehow connected at multiple levels (Chirikure 2020a).

Arguably, the recovery of a significant number of gold processing vessels from Great Zimbabwe and the scientific analyses performed, when considered using a regional approach, raise significant insights into early globalisation and entanglements within southern Africa and with the Indian Ocean (Killick 2009; Pikirayi 2017; Seetah 2018; Moffett and Hall 2020). In general, it is believed that the impetus to work gold in southern Africa came from the Indian Ocean towards the end of the first millennium CE (Swan 1994; Killick 2009). The appearance of custom-made crucibles is also associated with exchanges with the Indian Ocean (Miller 2010). What should also be emphasised, however, is that custom-made crucibles at Great Zimbabwe were, within variation, made of the same locally derived clays typical of the local granitic background. That is also the clay that was used to make pottery and, despite a small sample size, furnace walls. These crucibles co-existed in complementary ways with the local and versatile use of domestic ceramics repurposed for metallurgy. Although the idea to work gold might have resulted from exchanges with other regions, it was creatively localised, building it into local knowledge to establish a viable, large-scale gold-working industry that exploited pre-existing technology and infrastructure. As such, globalisation did not take away local agency; it encouraged it. It promoted experimentation and improvisation with materials, skills, and knowledge, creating products that were useful in multiple contexts. The evidence points to a localisation of ideas from elsewhere, ensuring that they fitted within local cultural understandings. This is also matched by a sizeable production, which was paired with local consumption.

Overall, the sizeable crucible assemblage recovered from a tiny section of Great Zimbabwe and the intensive nature of the production, when considered in light of gold reported by antiquarians, suggests a heavy use of gold locally and regionally, which would complement the well-documented intensive use of copper and bronze (Miller 2002; Bandama et al. 2016). Therefore, there is no need to assume that all gold worked in ancient mines ended up in the Indian Ocean exchange system. Rather, the integration of archaeological science with the other threads of information available reveals that gold was not only exploited and processed in multiple ways but also used locally and exported just like the other commodities. While issues of value require more elaboration (Chirikure 2014; Moffett and Chirikure 2016; Moffett and Hall 2020), Herbert (1984) showed that copper was the “red gold” of Africa, before and after the incorporation of regions such as southern Africa into the Indian Ocean system. However, that assessment was based on a limited understanding of the distribution of gold within multi-settlement sites such as Great Zimbabwe. It is now clear that gold was widely consumed and so was copper, raising vital questions about the choices that informed when to consume certain metals. Different metals offered different colour schema, and aesthetic and sensory values that correlated with ancestors, spirituality, and other domains of life (Chirikure 2014), as has also been noted for other materials such as beads (Bvocho 2005). The burial of an individual with significant amounts of copper-based objects at the *dzimbahwe* of Danamombe contrasts with the equally significant amount of gold recovered from the same site and others reported in the literature (Hall and Neal 1902). It might be possible that the Danamombe individual’s social role required that they were buried with copper, but we do not know what else was in that burial, or if gold was represented in significant quantities. Whatever the case, value was contextually negotiated to the extent that gold might have changed value depending on place, individual context, and social role of the bearers. As such, like all commodities, it likely did not have the same value, everywhere all the time.

Globalisation narratives that enshrine obsessions with gold and ignore the local are inappropriate and produce skewed histories. This has traditionally been the case not only in southern Africa but also in other colonial contexts. For example, analyses of gold processing crucibles from colonial Colombia have recently challenged facile narratives that simply saw gold as fuel for the European colonists. Instead, a creative interplay of traditions was revealed, involving multiple agents, identities, and motivations (Martinón-Torres et al. 2018). Similar arguments have been made recently regarding common archaeological inferences about the value and affective power of exotica (Moffett and Hall 2020; Moffett et al. 2021). As Moffett and Hall (2020, p. 314) have argued, “the widespread use of these value ascriptions has often resulted in the *assumption*, rather than demonstration, of the high value of long-distance trade objects in local contexts. As a result, less attention has been paid to exploring the various contributing factors to their valuation, along with the concepts of origin that may be variously activated” (emphasis added). As these authors note (Moffett and Hall 2020, p. 320; Moffett et al. 2021, p. 3), with particular reference to the circulation of Indian Ocean cowries within southern Africa, drawing on the work of Helms (1988), the geographical distance between the source of a manufactured commodity or raw material and the location of its use and eventual deposition may indeed be a source of value, but such value is rarely ascribed in a constant and consistent manner, even within the same cultural milieu. Given the accumulating evidence for the importance of local agency in determining the direction of flow, scale of exchange, and patterns of acquisition and circulation of glass and other beads across space and time during the southern African Iron Age (Wilmsen 2009; Robertshaw et al. 2010; Denbow et al. 2015), and the well-documented, and contextually variable, symbolism associated with things and materials of different colours among Great Zimbabwe’s likely descendant communities and other regional ethnicities (Bvocho 2005; Chirikure 2014; Motsamayi 2020), it seems inconceivable that the value of gold was not similarly contextually and historically contingent, even at the height of its regional and trans-oceanic circulation.

## Conclusion

Research into the previously neglected area of Great Zimbabwe, east of the Eastern Ridge Ruins, yielded a significant number of gold processing vessels, comprising a mix of reused domestic sherds and crucibles. Fabric analyses showed that both technical and domestic ceramics were made using local clays, while analyses of slagged layers showed that gold dust was melted and refined on site and consolidated into relatively standardised ingots. The chemical composition of metal prills set in the slag suggests that both hydrothermal and placer gold were worked at Great Zimbabwe. Most likely, the gold was sourced from different areas, pointing to multidirectional circulation networks within southern Africa. As far as the spatial concentration of production is concerned, the individual settlements making up Great Zimbabwe processed, worked, and used gold inside and outside the walls. Indeed, when added to the picture presented by Euro-American explorers of the late nineteenth and early twentieth centuries, it becomes clear that there was massive local consumption of gold on a scale much larger than traditionally allowed in some archaeological narratives. The scientific analyses have shown that in the process of working and manipulating gold, Great Zimbabwe interacted with local and regional communities as well as with Indian Ocean networks. Consequently, globalisation must be understood in local terms, and how the local shaped the organisation of production, raw material selection, consumption, and exporting the surplus regionally into southern Africa and the wider Indian Ocean area. Ultimately, gold working was pivoted on established knowledge practices and was punctuated by situational improvisation and innovation which allowed localised and regional exchanges that prompted different outcomes to parties involved in the interchange of materials and ideas. Contrary to traditional approaches that consigned the local to passivity, the story of globalisation involving gold at Great Zimbabwe actively engaged the local, the individual, and the collective within Africa.

## Supplementary Information

Below is the link to the electronic supplementary material.

Supplementary file1 (PDF 12.6 MB)

## Data Availability

All of the compositional data, including the results of individual area analyses for calculating the average bulk, matrix, and slag compositions, as well as the compositions of inclusions and metallic prills contained within the ceramics, are available either in the main body of text or in the supplementary material. Additional artefact photographs and photomicrographs are also provided in the supplementary material.
