# Supplementary material for: Archaeological science, globalisation, and local agency: gold in Great Zimbabwe
Source: Archaeol Anthropol Sci. 2023 Aug 3;15(8):127. doi: 10.1007/s12520-023-01811-7 (PMC10400708; doi:10.1007/s12520-023-01811-7)
Supplement: Supplementary file 1 — Supplementary file1 (PDF 12.6 MB) [file 12520_2023_1811_MOESM1_ESM.pdf]

ARCHAEOLOGICAL SCIENCE, GLOBALISATION AND LOCAL AGENCY: GOLD IN GREAT ZIMBABWE

Vieri, Jasmine[a*], Chirikure, Shadreck[b,c], Lane, Paul[a,d,e] and Martinón-Torres, Marcos[a,d]

Supplementary Materials

*jmkv2@cam.ac.uk

a. McDonald Institute for Archaeological Research, University of Cambridge, Downing Street, Cambridge CB2 3ER, United Kingdom

b. Research Laboratory for Archaeology and the History of Art, School of Archaeology, University of Oxford, 1 South Parks Road, Oxford OX1 3TG, United Kingdom

c. Department of Archaeology, University of Cape Town, Private Bag X3, Rondebosch 7701, South Africa

d. Department of Archaeology, University of Cambridge, Downing Street, Cambridge CB2 3DZ, United Kingdom

e. School of Geography, Archaeology & Environmental Studies, University of the Witwatersrand, 1 Jan Smuts Avenue, Braamfontein 2000, Johannesburg, South Africa

Contents

S0:Pfupiso (Shona abstract) **2**

S1:Basalt standard **3**

S2:Composition of inclusions and prills in all samples **4**

S3:Detailed results for individual samples **8**

1 CA200170 (GZ2) . . . 8
2 CA200171 (GZ4) . . . 11
3 CA200172 (GZ10) . . . 14
4 CA200173 (GZ12) . . . 17
5 CA200174 (GZ1) . . . 20
6 CA200175 (GZ6) . . . 23
7 CA200176 (GZ8) . . . 25
8 CA200177 (GZ11) . . . 28
9 CA200178 (GZ7) . . . 31
10 CA200179 (GZ9) . . . 34
11 CA200180 (GZ3) . . . 36
12 CA200181 (GZ5) . . . 38
13 CA200182 (GZ13) . . . 40

S0: Pfupiso (Shona abstract)

Dzimbahwe reMasvingo eZimbabwe rine mukurumbira pasi rose maererano neunyanzvi hwekupfura simbi nekutenga nekutengeserana nevagari vemunzvimbo dziri muAfrica nedzemhiri kwegungwa rekumabvazuva. Basa rekutsvaka zvakasiyiwa nevakuru vekare vaigara mudzimbahwe rakawana midziyo yaishandiswa kunyungudutsa nekugadzirisa mukute (goridhe). Midziyo iyi yaisanganisira zvainga (zvehari) nezvikari zvaigadzirirwa kunyungudutsa mukute. Takatora muenzaniso wezvishandiswa izvi tikaongoora nemuchina kuti zvakagadzirwa seiko uye kuti simbi dzaigadzirwa umu dzaiva dzemhando ipiko. Midziyo iyi yakagadzirwa neivhu rechinamwe rinowanikwa munharaunda yemusha wedzimbahwe reMasvingo eZimbabwe. Chinamwe chacho chaisarudzwa neunyanzvi kuitira kuti midziyo yacho isatsemuka pakushanda basa risati rapera. Tsakurudzo dzakaratidza kuti midziyo iyi yaishandiswa kugadzira mukute, nekuupima kuisa muuwandu hwekutengesa nahwo. Kunyangwe Masvingo eZimbabwe akawirwa netsaona yekucherwacherwa nekubiwa nevapambi pfumi, tsvakurudzo dzedu dzinoratidza kuti vagari vepaMasvingo eZimbabwe vaive mhizha dzaitengesera vanhu vekune dzimwe nzvimbo mubato wemaoko wakagadzirwa neunyanzvi. Basa ratakaita rinoratidza kuti pakutaura nhoroondo tinofanira kusimbisa kubhindauka kwevakuru vekare neumhizha hwavo hwakaita kuti pave nekusangana nekutengeserana nevagari venzvimbo dzakasiyana siyana dzemukati nedzekunze kweAfrica.

S1: Basalt standard

Table S1.1: Basalt standard BCR-2G (all values reported as normalised wt%)

**BCR-2G**	**Na_2O**	**MgO**	**Al_2O_3**	**SiO_2**	**P_2O_5**	**K_2O**	**CaO**	**TiO_2**	**MnO**	**FeO**	**Analytical total**
Analysis 1	3.2	3.7	13.6	54.0	0.4	1.8	7.4	2.4	0.2	13.3	101.4
Analysis 2	3.2	3.6	13.7	54.5	0.3	1.9	7.3	2.4	0.2	12.9	102.6
Analysis 3	3.1	3.6	13.6	55.0	0.3	1.9	7.2	2.5	0.2	12.6	99.5
Standard deviation (SD)	0.1	0.1	0.1	0.5	0.1	0.1	0.1	0.1	0.0	0.4	
Coefficient of variation (CV)	0.0	0.0	0.0	0.0	0.2	0.0	0.0	0.0	0.0	0.0	
Mean	3.2	3.6	13.6	54.6	0.3	1.9	7.3	2.4	0.2	12.9	101.2
Given (CRM)	3.2	3.6	13.7	55.0	0.4	1.8	7.2	2.3	0.2	12.6	
Absolute error (δ)	0.0	0.0	-0.1	-0.4	-0.1	0.1	0.1	0.1	0.0	0.3	
Relative error (δ, %)	0.0	0.0	0.7	0.7	25.0	5.6	1.4	4.3	0.0	2.4	

S2: Composition of inclusions and prills in all samples

Table S2.1: Inclusions in domestic repurposed ceramics (all values reported as normalised wt%)

**DOMESTIC REPURPOSED**	**Na_2O**	**MgO**	**Al_2O_3**	**SiO_2**	**P_2O_5**	**K_2O**	**CaO**	**TiO**	**V_2O_5**	**Cr_2O_3**	**MnO**	**FeO**	**Y_2O_3**	**ZrO_2**	**Nb_2O_5**	**Ag_2O**	**In_2O_3**	**BaO**	**HfO_2**	**Ce_2O_3**	**PbO**	**UO_3**	**Analytical total**
CA200174 Zircon	n.d.	n.d.	n.d.	30.7	n.d.	n.d.	n.d.	n.d.	n.d.	n.d.	n.d.	n.d.	n.d.	66.1	1.9	n.d.	n.d.	n.d.	1.3	n.d.	n.d.	n.d.	108.8
CA200174 Zircon	n.d.	n.d.	2.7	32.4	n.d.	n.d.	0.5	n.d.	n.d.	n.d.	n.d.	3.8	n.d.	60.0	n.d.	n.d.	n.d.	n.d.	n.d.	0.7	n.d.	n.d.	125.5
CA200174 Potassium feldspar	3.1	n.d.	19.2	65.5	n.d.	9.1	0.9	n.d.	n.d.	n.d.	n.d.	1.4	n.d.	n.d.	n.d.	n.d.	n.d.	0.9	n.d.	n.d.	n.d.	n.d.	105.5
CA200174 Alumina silicate	0.8	n.d.	28.4	43.6	n.d.	1.3	13.1	0.8	n.d.	n.d.	n.d.	12.0	n.d.	n.d.	n.d.	n.d.	n.d.	n.d.	n.d.	n.d.	n.d.	n.d.	93.0
CA200174 Ti-rich mineral	n.d.	1.4	11.5	7.3	0.3	0.8	n.d.	61.2	0.7	n.d.	n.d.	16.2	n.d.	n.d.	0.6	n.d.	n.d.	n.d.	n.d.	n.d.	n.d.	n.d.	104.5
CA200175 Iron oxide	n.d.	n.d.	0.3	0.6	0.3	n.d.	0.1	n.d.	0.2	n.d.	n.d.	98.6	n.d.	n.d.	n.d.	n.d.	n.d.	n.d.	n.d.	n.d.	n.d.	n.d.	100.5
CA200175 Potassium feldspar	0.8	n.d.	19.2	62.0	n.d.	14.6	n.d.	n.d.	n.d.	n.d.	n.d.	0.2	n.d.	n.d.	n.d.	n.d.	n.d.	3.2	n.d.	n.d.	n.d.	n.d.	75.8
CA200175 Soda feldspar	8.2	n.d.	24.4	61.5	n.d.	0.1	5.6	n.d.	n.d.	n.d.	n.d.	0.2	n.d.	n.d.	n.d.	n.d.	n.d.	n.d.	n.d.	n.d.	n.d.	n.d.	103.6
CA200175 Alumina silicate	n.d.	n.d.	25.2	37.1	n.d.	0.1	22.3	n.d.	n.d.	n.d.	n.d.	15.3	n.d.	n.d.	n.d.	n.d.	n.d.	n.d.	n.d.	n.d.	n.d.	n.d.	87.3
CA200175 Mg-rich mineral	0.7	17.4	4.5	53.2	n.d.	0.2	12.5	0.2	n.d.	0.1	0.3	10.9	n.d.	n.d.	n.d.	n.d.	n.d.	n.d.	n.d.	n.d.	n.d.	n.d.	88.1
CA200175 Ilmenite	n.d.	n.d.	n.d.	0.2	n.d.	n.d.	0.1	50.1	0.5	n.d.	4.5	44.5	n.d.	n.d.	n.d.	n.d.	n.d.	n.d.	n.d.	n.d.	n.d.	n.d.	78.7
CA200175 Rutile	n.d.	n.d.	0.9	0.9	n.d.	0.1	0.2	95.1	n.d.	n.d.	n.d.	2.4	n.d.	n.d.	0.4	n.d.	n.d.	n.d.	n.d.	n.d.	n.d.	n.d.	102.3
CA200176 Zircon	n.d.	n.d.	1.8	27.9	n.d.	n.d.	0.5	n.d.	n.d.	n.d.	n.d.	2.5	3.2	56.0	n.d.	2.3	n.d.	n.d.	1.6	2.1	n.d.	2.1	71.1
CA200176 Potassium feldspar	1.6	n.d.	18.9	64.8	n.d.	14.6	n.d.	n.d.	n.d.	n.d.	n.d.	0.2	n.d.	n.d.	n.d.	n.d.	n.d.	n.d.	n.d.	n.d.	n.d.	n.d.	105.1
CA200176 Soda feldspar	10.2	n.d.	21.8	66.7	n.d.	0.9	n.d.	n.d.	n.d.	n.d.	n.d.	0.4	n.d.	n.d.	n.d.	n.d.	n.d.	n.d.	n.d.	n.d.	n.d.	n.d.	97.5
CA200176 Ilmenite	n.d.	n.d.	0.7	n.d.	n.d.	n.d.	n.d.	57.5	0.5	n.d.	1.0	40.2	n.d.	n.d.	n.d.	n.d.	n.d.	n.d.	n.d.	n.d.	n.d.	n.d.	93.9
CA200176 Fe-rich mineral	n.d.	1.2	21.0	16.2	n.d.	0.5	0.8	0.6	0.3	0.4	n.d.	59.0	n.d.	n.d.	n.d.	n.d.	n.d.	n.d.	n.d.	n.d.	n.d.	n.d.	66.7
CA200177 Potassium feldspar	1.2	n.d.	18.8	64.4	n.d.	15.0	n.d.	n.d.	n.d.	n.d.	n.d.	n.d.	n.d.	n.d.	n.d.	n.d.	n.d.	0.6	n.d.	n.d.	n.d.	n.d.	109.0
CA200177 Soda feldspar	8.4	n.d.	22.5	62.5	n.d.	1.7	2.2	n.d.	n.d.	n.d.	n.d.	2.8	n.d.	n.d.	n.d.	n.d.	n.d.	n.d.	n.d.	n.d.	n.d.	n.d.	78.6
CA200177 Ti-rich mineral	n.d.	12.9	14.0	1.0	1.2	0.5	n.d.	57.8	0.8	n.d.	n.d.	11.2	n.d.	n.d.	n.d.	n.d.	n.d.	n.d.	n.d.	n.d.	0.5	n.d.	70.3
CA200177 Rutile	n.d.	n.d.	3.7	0.7	n.d.	n.d.	n.d.	94.7	n.d.	n.d.	n.d.	0.5	n.d.	n.d.	0.3	n.d.	0.2	n.d.	n.d.	n.d.	n.d.	n.d.	111.3
CA200177 Fe-rich mineral	0.9	1.0	18.6	29.2	1.2	2.9	0.7	0.4	0.2	n.d.	n.d.	44.5	n.d.	n.d.	n.d.	n.d.	n.d.	n.d.	n.d.	n.d.	0.5	n.d.	100.4
CA200179 Zircon	n.d.	n.d.	31.3	n.d.	n.d.	n.d.	n.d.	n.d.	n.d.	n.d.	n.d.	n.d.	n.d.	67.4	n.d.	n.d.	n.d.	n.d.	1.3	n.d.	n.d.	n.d.	81.3
CA200179 Potassium feldspar	1.0	n.d.	19.8	62.2	n.d.	14.5	n.d.	n.d.	n.d.	n.d.	n.d.	2.6	n.d.	n.d.	n.d.	n.d.	n.d.	n.d.	n.d.	n.d.	n.d.	n.d.	89.4
CA200179 Ilmenite	n.d.	n.d.	2.4	3.1	n.d.	n.d.	n.d.	52.0	n.d.	0.3	1.9	40.3	n.d.	n.d.	n.d.	n.d.	n.d.	n.d.	n.d.	n.d.	n.d.	n.d.	101.6
CA200179 Mineral with Na/Ca	6.5	n.d.	23.1	62.8	n.d.	0.9	5.0	n.d.	n.d.	n.d.	n.d.	1.7	n.d.	n.d.	n.d.	n.d.	n.d.	n.d.	n.d.	n.d.	n.d.	n.d.	99.7
CA200179 Chromite	n.d.	n.d.	7.3	0.3	0.4	n.d.	0.3	n.d.	n.d.	55.4	n.d.	36.3	n.d.	n.d.	n.d.	n.d.	n.d.	n.d.	n.d.	n.d.	n.d.	n.d.	106.6
CA200179 Fe-rich mineral	0.7	1.7	24.9	22.4	0.3	0.7	0.8	0.4	0.2	0.2	n.d.	47.6	n.d.	n.d.	n.d.	n.d.	n.d.	n.d.	n.d.	n.d.	n.d.	n.d.	104.2
**Rare earth minerals**	**Al_2O_3**	**SiO_2**	**P_2O_5**	**CaO**	**V_2O_5**	**FeO**	**Y_2O_3**	**La_2O_3**	**Ce_2O_3**	**Pr_2O_3**	**Nd_2O_3**	**Gd_2O_3**	**ThO_2**										**Analytical total**
CA200176 Thorite with RE elements	4.8	19.2	3.6	n.d.	1.1	3.6	1.6	n.d.	2.0	n.d.	1.2	0.6	62.3										109.9

Table S2.2: Inclusions in purpose-made crucibles (all values reported as normalised wt%)

**PURPOSE-MADE**	Na_2O	MgO	Al_2O_3	SiO_2	P_2O_5	K_2O	CaO	Sc_2O_3	TiO	Cr_2O_3	MnO	FeO	Y_2O_3	ZrO_2	Nb_2O_5	SnO_2	BaO	HfO_2	ThO_2	UO_3	Analytical total
CA200170 Zircon	n.d.	n.d.	2.0	31.9	n.d.	0.2	0.3	n.d.	0.3	n.d.	n.d.	0.7	n.d.	61.4	1.6	n.d.	n.d.	1.5	n.d.	n.d.	111.4
CA200170 Potassium feldspar	0.5	n.d.	18.7	64.9	n.d.	15.3	n.d.	n.d.	n.d.	n.d.	n.d.	0.5	n.d.	n.d.	n.d.	n.d.	n.d.	n.d.	n.d.	n.d.	92.1
CA200170 Rutile	n.d.	n.d.	0.7	1.4	n.d.	0.1	n.d.	n.d.	96.2	n.d.	n.d.	0.7	n.d.	n.d.	0.9	n.d.	n.d.	n.d.	n.d.	n.d.	75.9
CA200171 Zircon	n.d.	n.d.	0.7	31.6	n.d.	0.4	n.d.	n.d.	n.d.	n.d.	n.d.	0.6	n.d.	64.9	n.d.	n.d.	n.d.	1.9	n.d.	n.d.	98.3
CA200171 Potassium feldspar	0.7	n.d.	18.9	64.5	n.d.	14.7	n.d.	n.d.	n.d.	n.d.	n.d.	n.d.	n.d.	n.d.	n.d.	0.8	0.5	n.d.	n.d.	n.d.	115.3
CA200171 Rutile	n.d.	n.d.	0.7	1.4	n.d.	0.1	n.d.	n.d.	96.2	n.d.	n.d.	0.7	n.d.	n.d.	0.9	n.d.	n.d.	n.d.	n.d.	n.d.	75.9
CA200172 Zircon	n.d.	n.d.	n.d.	31.5	n.d.	n.d.	n.d.	n.d.	n.d.	n.d.	n.d.	n.d.	n.d.	67.0	n.d.	n.d.	n.d.	1.5	n.d.	n.d.	90.4
CA200172 Potassium feldspar	0.4	n.d.	19.6	66.1	n.d.	13.0	n.d.	n.d.	n.d.	n.d.	n.d.	0.9	n.d.	n.d.	n.d.	n.d.	n.d.	n.d.	n.d.	n.d.	99.3
CA200172 U-rich thorite	n.d.	n.d.	n.d.	21.1	0.9	n.d.	n.d.	n.d.	n.d.	n.d.	n.d.	n.d.	1.0	n.d.	n.d.	n.d.	n.d.	n.d.	64.9	12.1	87.7
CA200172 Chromite	n.d.	n.d.	31.7	7.5	n.d.	0.6	n.d.	n.d.	1.2	51.6	n.d.	7.4	n.d.	n.d.	n.d.	n.d.	n.d.	n.d.	n.d.	n.d.	102.9
CA200173 Zircon	n.d.	0.7	6.2	34.2	n.d.	1.0	1.0	0.3	0.4	n.d.	n.d.	1.3	n.d.	51.1	n.d.	n.d.	n.d.	0.9	2.9	n.d.	66.6
CA200173 Potassium feldspar	0.6	n.d.	19.7	64.6	n.d.	14.5	n.d.	n.d.	n.d.	n.d.	n.d.	0.4	n.d.	n.d.	n.d.	n.d.	0.3	n.d.	n.d.	n.d.	78.4
CA200173 Ilmenite	n.d.	n.d.	2.4	3.8	n.d.	0.5	0.1	n.d.	45.4	n.d.	3.5	43.8	n.d.	n.d.	0.5	n.d.	n.d.	n.d.	n.d.	n.d.	99.7
CA200173 Rutile	n.d.	n.d.	3.2	3.6	n.d.	0.4	n.d.	n.d.	92.9	n.d.	n.d.	n.d.	n.d.	n.d.	n.d.	n.d.	n.d.	n.d.	n.d.	n.d.	101.6

**Rare earth minerals**	Al_2O_3	SiO_2	P_2O_5	K_2O	CaO	FeO	As_2O_3	Y_2O_3	La_2O_3	Ce_2O_3	Pr_2O_3	Nd_2O_3	Sm_2O_3	Gd_2O_3	Dy_2O_3	Ho_2O_3	Er_2O_3	ThO_2	UO_3		Analytical total
CA200170 Monazite	0.4	1.1	28.9	0.2	n.d.	n.d.	n.d.	n.d.	17.3	33.3	3.1	10.4	1.5	0.9	n.d.	n.d.	n.d.	2.9	n.d.		89.8
CA200171 Xenotime	10.7	11.9	27.7	1.0	n.d.	0.5	n.d.	38.2	n.d.	n.d.	n.d.	n.d.	n.d.	1.5	4.9	0.9	2.7	n.d.	n.d.		105.3
CA200172 Monazite	1.0	3.1	30.4	0.5	1.0	n.d.	n.d.	n.d.	14.0	27.5	2.2	11.6	1.9	1.7	n.d.	n.d.	n.d.	4.2	1.0		64.2
CA200173 Monazite	1.8	3.3	28.1	0.3	n.d.	0.4	3.4	n.d.	21.6	31.3	2.2	7.6	n.d.	n.d.	n.d.	n.d.	n.d.	n.d.	n.d.		72.1

Table S2.3: Inclusions in furnace wall CA200178 (all values reported as normalised wt%)

**FURNACE WALL**	Na_2O	Al_2O_3	SiO_2	P_2O_5	K_2O	TiO	V_2O_5	Cr_2O_3	MnO	FeO	ZrO_2	Ag_2O	HfO_2	UO_3	Analytical total
CA200178 Zircon	n.d.	3.2	33.7	n.d.	0.2	n.d.	n.d.	n.d.	n.d.	4.0	55.2	0.8	1.5	1.3	94.9
CA200178 Potassium feldspar	0.4	18.8	65.1	n.d.	14.2	n.d.	n.d.	n.d.	n.d.	1.6	n.d.	n.d.	n.d.	n.d.	91.5
CA200178 Ti-rich mineral	n.d.	2.5	1.1	0.3	n.d.	84.0	0.8	0.6	0.8	9.9	n.d.	n.d.	n.d.	n.d.	105.3
CA200178 Ilmenite	n.d	1.0	n.d.	n.d.	n.d.	54.3	n.d.	n.d.	2.9	41.8	n.d.	n.d.	n.d.	n.d.	103.8

**Rare earth minerals**	Al_2O_3	SiO_2	P_2O_5	CaO	V_2O_5	FeO	Y_2O_3	La_2O_3	Ce_2O_3	Pr_2O_3	Nd_2O_3	Gd_2O_3	ThO_2		Analytical total
CA200178 Monazite	n.d.	2.0	28.4	0.5	n.d.	n.d.	n.d.	17.2	32.0	2.6	8.5	n.d.	8.8		93.4

Table S2.4: Inclusions in domestic pottery (all values reported as normalised wt%)

**DOMESTIC POTTERY**	**Na_2O**	**MgO**	**Al_2O_3**	**SiO_2**	**P_2O_5**	**K_2O**	**CaO**	**TiO**	**V_2O_5**	**Cr_2O_3**	**MnO**	**FeO**	**ZrO_2**	**Nb_2O_5**	**Ag_2O**	**SnO_2**	**BaO**	**HfO_2**	**Analytical total**
CA200180 Zircon	n.d.	n.d.	1.4	30.1	n.d.	n.d.	1.0	n.d.	n.d.	n.d.	n.d.	2.7	61.9	n.d.	1.2	n.d.	n.d.	1.8	75.5
CA200180 Potassium feldspar	0.4	n.d.	18.8	64.7	n.d.	16.0	n.d.	n.d.	n.d.	n.d.	n.d.	n.d.	n.d.	n.d.	n.d.	n.d.	n.d.	n.d.	98.6
CA200180 Soda feldspar	9.2	0.2	21.9	64.4	0.4	0.4	1.1	0.3	n.d.	n.d.	n.d.	2.1	n.d.	n.d.	n.d.	n.d.	n.d.	n.d.	63.0
CA200180 Ilmenite	n.d.	n.d.	0.8	0.6	n.d.	n.d.	n.d.	56.1	n.d.	n.d.	1.0	41.6	n.d.	n.d.	n.d.	n.d.	n.d.	n.d.	84.0
CA200180 Rutile	n.d.	n.d.	0.9	1.1	n.d.	n.d.	0.3	91.4	1.3	n.d.	n.d.	5.0	n.d.	n.d.	n.d.	n.d.	n.d.	n.d.	74.1
CA200181 Potassium feldspar	0.4	n.d.	18.8	63.4	n.d.	16.6	n.d.	n.d.	n.d.	n.d.	n.d.	n.d.	n.d.	n.d.	n.d.	0.9	n.d.	n.d.	72.5
CA200181 Potassium feldspar	0.5	n.d.	18.7	62.0	n.d.	14.8	n.d.	n.d.	n.d.	n.d.	n.d.	4.0	n.d.	n.d.	n.d.	n.d.	n.d.	n.d.	99.5
CA200181 Ilmenite	n.d.	n.d.	n.d.	n.d.	n.d.	n.d.	n.d.	55.9	n.d.	n.d.	1.4	42.7	n.d.	n.d.	n.d.	n.d.	n.d.	n.d.	100.0
CA200181 Fe-rich mineral	n.d.	n.d.	10.8	13.8	0.4	n.d.	1.0	n.d.	n.d.	0.3	n.d.	73.7	n.d.	n.d.	n.d.	n.d.	n.d.	n.d.	105.3
CA200182 Zircon	n.d.	n.d.	0.7	30.7	n.d.	n.d.	0.6	n.d.	n.d.	n.d.	n.d.	2.5	64.6	n.d.	n.d.	n.d.	n.d.	1.0	95.3
CA200182 Potassium feldspar	0.5	n.d.	18.8	64.9	n.d.	15.5	n.d.	n.d.	n.d.	n.d.	n.d.	n.d.	n.d.	n.d.	n.d.	n.d.	0.3	n.d.	95.9
CA200182 Alumina silicate	n.d.	n.d.	24.4	40.4	n.d.	n.d.	22.3	n.d.	n.d.	n.d.	n.d.	12.9	n.d.	n.d.	n.d.	n.d.	n.d.	n.d.	95.7
CA200182 Rutile	n.d.	n.d.	2.5	4.1	1.6	n.d.	1.0	83.5	1.0	n.d.	n.d.	4.3	n.d.	1.6	0.3	n.d.	n.d.	n.d.	77.4

Table S2.5: Composition of prills (all values reported as normalised wt%)

**DOMESTIC REPURPOSED**	**Cu**	**As**	**Ag**	**Au**	**Diameter**	**Location**
CA200174	n.d.	n.d.	n.d.	100.0	3µm	Slag
CA200174	n.d.	n.d.	0.7	99.3	1µm	Ceramic
CA200176	n.d.	n.d.	1.3	98.7	5µm	Slag
CA200176	n.d.	n.d.	1.4	98.6	3µm	Slag
CA200176	n.d.	n.d.	1.5	98.5	15µm	Slag
CA200176	n.d.	n.d.	1.7	98.3	30µm	Slag
CA200177	1.5	n.d.	8.6	89.8	20µm	Slag
CA200177	13.5	n.d.	19.3	67.3	7µm	Slag
CA200179	n.d.	n.d.	10.1	89.9	4500µm	Slag
CA200179	n.d.	n.d.	9.0	91.0	25µm	Slag
CA200179	n.d.	n.d.	12.5.	87.5	20µm	Slag
CA200179	n.d.	n.d.	100.0	n.d.	1µm	Ceramic close to slag
**PURPOSE-MADE**						
CA200170	n.d.	n.d.	4.5	95.5	12µm	Slag
CA200170	n.d.	n.d.	4.9	95.1	6µm	Slag
CA200170	n.d.	n.d.	4.8	95.2	7µm	Slag
CA200170	n.d.	n.d.	4.1	95.9	4µm	Slag
CA200170	n.d.	n.d.	3.9	96.1	9µm	Slag
CA200170	n.d.	n.d.	2.5	97.5	14µm	Slag
CA200170	n.d.	n.d.	0.5	99.5	5µm	Slag
CA200170	n.d.	n.d.	2.6	97.4	7µm	Slag
CA200171	1.4	n.d.	7.0	91.6	32µm	Slag
CA200171	n.d.	n.d.	bdl	100.0	1µm	Slag
CA200171	98.4	1.6	n.d.	n.d.	8µm	Ceramic
CA200173	n.d.	n.d.	10.4	89.6	30µm	Slag
CA200173	n.d.	n.d.	12.0	88.0	9µm	Slag
CA200173	n.d.	n.d.	11.5	88.5	5µm	Slag
CA200173	n.d.	n.d.	12.3	87.7	10µm	Slag
CA200173	n.d.	n.d.	10.7	89.3	7µm	Slag
CA200173	n.d.	n.d.	9.7	90.3	8µm	Slag

S3: Detailed results for individual samples

1 CA200170 (GZ2)

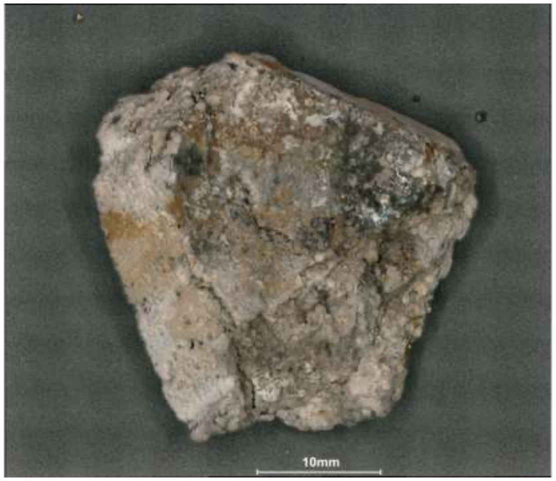

(a) Sample CA200170

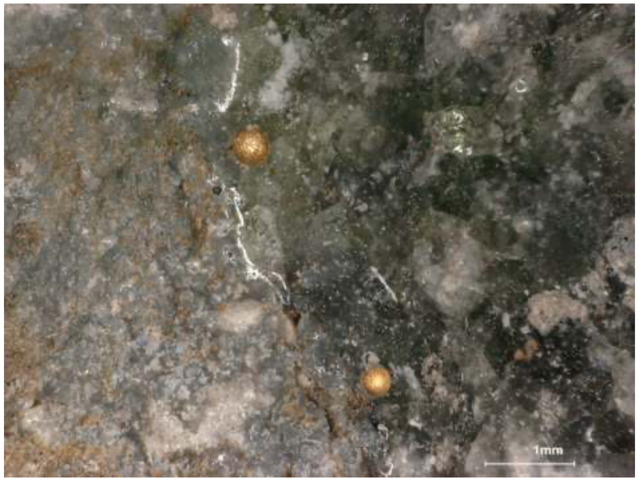

(b) Prills on the surface of CA200170)

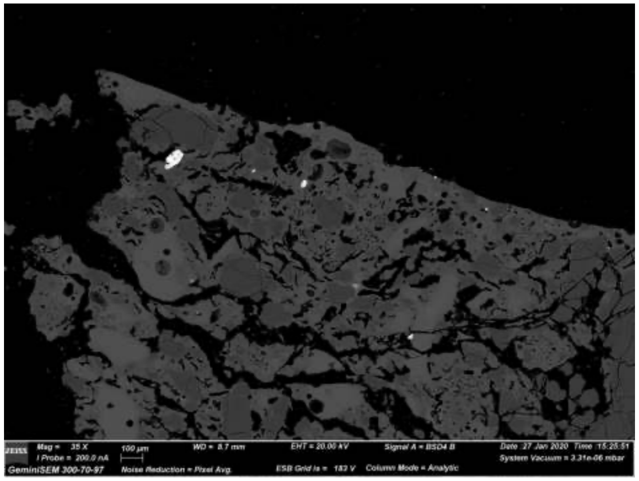

(c) Interphase between ceramic and slag (35x)

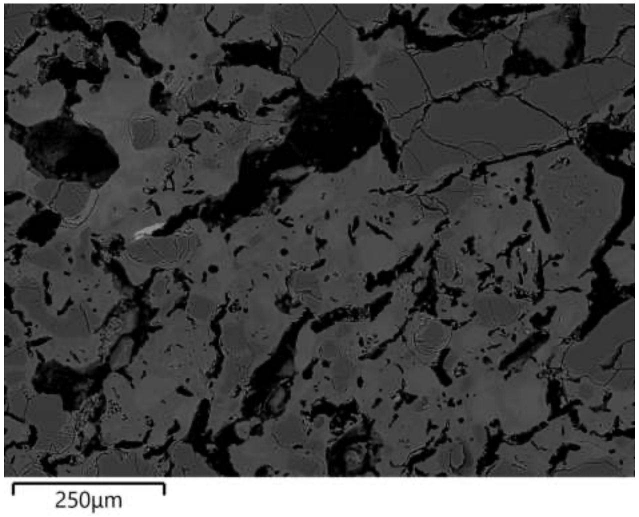

(d) Bulk ceramic (100x)

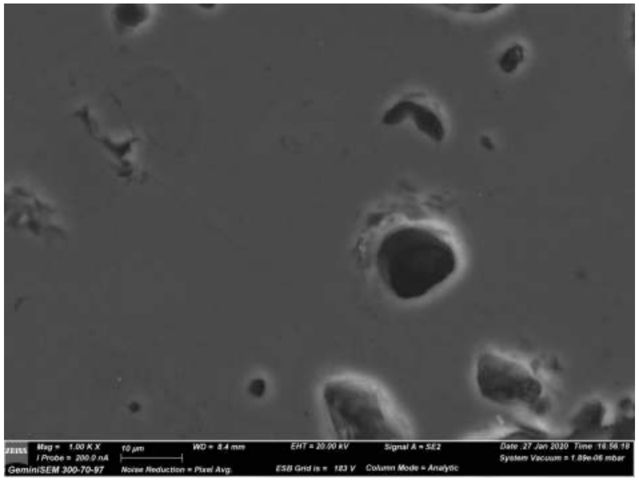

(e) Ceramic matrix (1000x)

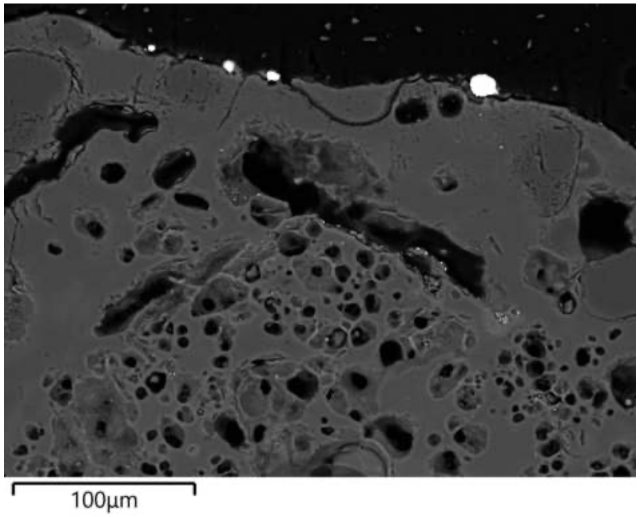

(f) Prills

Figure S3.1: CA200170

Table S3.1.1: CA200170 Bulk ceramic (all values reported as normalised wt%)

**CA200170**	**MgO**	**Al_2O_3**	**SiO_2**	**K_2O**	**CaO**	**TiO_2**	**FeO**	**Analytical total**
Analysis 1	n.d.	23.3	70.4	3.4	0.4	0.7	1.9	68.6
Analysis 2	0.1	22.3	71.5	4.0	n.d.	0.5	1.6	76.8
Analysis 3	n.d.	22.2	70.8	4.6	n.d.	0.7	1.8	76.8
Mean	≤0.1	22.5	70.6	4.0	≤0.4	0.6	1.7	74.3
Standard deviation (SD)	n/a	0.6	0.5	0.6	n/a	0.1	0.1	
Coefficient of variation (CV)	n/a	0.0	0.0	0.1	n/a	0.2	0.1	

Table S3.1.2: CA200170 Ceramic matrix (all values reported as normalised wt%)

**CA200170**	**MgO**	**Al_2O_3**	**SiO_2**	**K_2O**	**TiO**	**FeO**	**Analytical total**
Analysis 1	n.d.	35.2	57.4	4.6	0.8	2.1	97.4
Analysis 2	n.d.	38.0	55.2	3.3	0.9	2.6	100.7
Analysis 3	0.2	36.5	56.0	4.9	0.9	1.5	93.5
Mean	≤0.2	36.5	56.1	4.3	0.9	2.0	97.3
Standard deviation (SD)	n/a	1.4	1.1	0.8	0.1	0.5	
Coefficient of variation (CV)	n/a	0.0	0.0	0.2	0.1	0.3	

Table S3.1.3: CA200170 Inclusions (all values reported as normalised wt%)

**CA200170**	**Na_2O**	**Al_2O_3**	**SiO_2**	**K_2O**	**CaO**	**TiO**	**FeO**	**ZrO_2**	**Nb_2O_5**	**HfO_2**		**Analytical total**
Zircon	n.d.	2.0	31.9	0.2	0.3	0.3	0.7	61.4	1.6	1.5		111.4
Potassium feldspar	0.5	18.7	64.9	15.3	n.d.	n.d.	0.5	n.d.	n.d.	n.d.		92.1
Rutile	n.d.	0.7	1.4	0.1	n.d.	96.2	0.7	n.d.	0.9	n.d.		75.9
**CA200170**	**Al_2O_3**	**SiO_2**	**P_2O_5**	**K_2O**	**La_2O_3**	**Ce_2O_3**	**Pr_2O_3**	**Nd_2O_3**	**Sm_2O_3**	**Gd_2O_3**	**ThO_2**	**Analytical total**
Monazite	0.4	1.1	28.9	0.2	17.3	33.3	3.1	10.4	1.5	0.9	2.9	89.8

Table S3.1.4: CA200170 Slag (all values reported as normalised wt%)

**CA200170**	**Na_2O**	**MgO**	**Al_2O_3**	**SiO_2**	**K_2O**	**CaO**	**TiO_2**	**FeO**	**Analytical total**
Analysis 1	0.2	0.3	23.2	62.4	6.0	7.2	0.4	0.4	98.0
Analysis 2	n.d.	0.3	28.1	61.3	6.4	2.1	0.4	1.5	94.1
Analysis 3	n.d.	n.d.	23.9	67.6	5.3	1.3	0.3	1.5	94.0
Mean	$\leq$0.2	0.2	25.0	63.7	5.9	3.5	0.4	1.1	95.6
Standard deviation (SD)	n/a	0.2	2.7	3.4	0.6	3.2	0.0	0.6	
Coefficient of variation (CV)	n/a	0.9	0.1	0.1	0.1	0.9	0.1	0.6	

Table S3.1.5: CA200170 Prills (all values reported as normalised wt%)

**CA200170**	**O**	**Fe**	**Ag**	**Au**	**Analytical total**	**Size of prill**
Analysis 1	2.2	n.d.	4.4	93.5	97.0	12µm
Analysis 2	2.0	n.d.	4.8	93.1	85.3	6µm
Analysis 3	2.5	n.d.	4.7	92.8	109.0	7µm
Analysis 4	2.7	n.d.	4.0	93.3	104.3	4µm
Analysis 5	8.3	5.8	3.3	82.6	95.4	9µm
Analysis 6	2.3	n.d.	2.5	95.2	84.9	14µm
Analysis 7	2.1	n.d.	0.5	97.4	89.6	5µm
Analysis 8	2.8	0.4	2.5	94.3	87.4	7µm

2 CA200171 (GZ4)

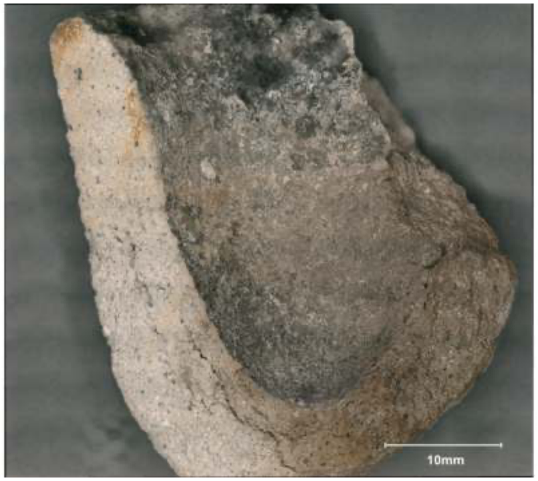

(a) Sample CA200171

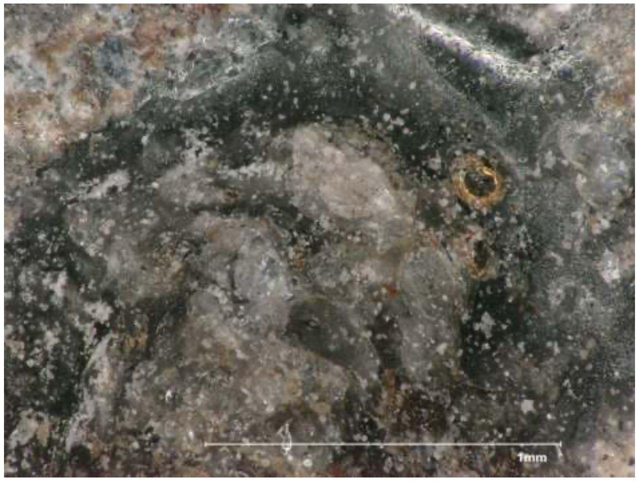

(b) Prill on the surface of CA200171

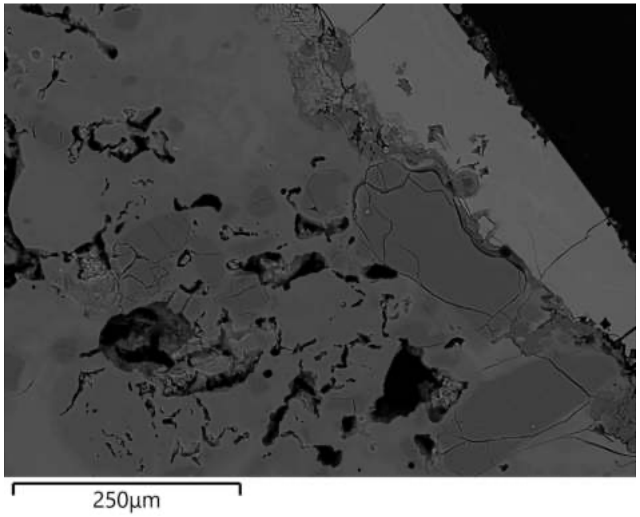

(c) Interphase between ceramic and slag (100x)

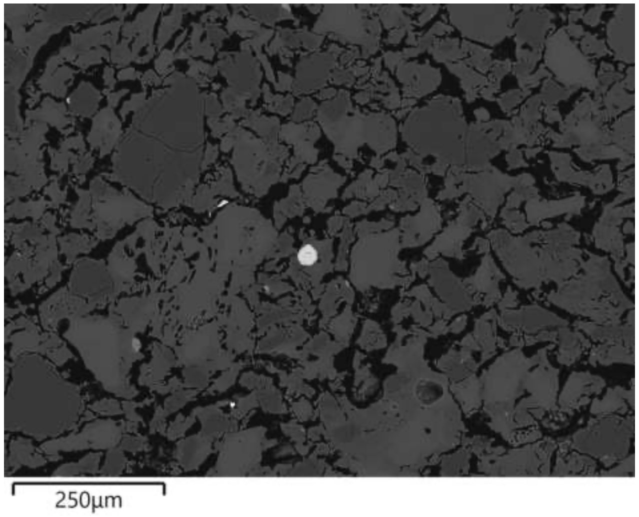

(d) Bulk ceramic (100x)

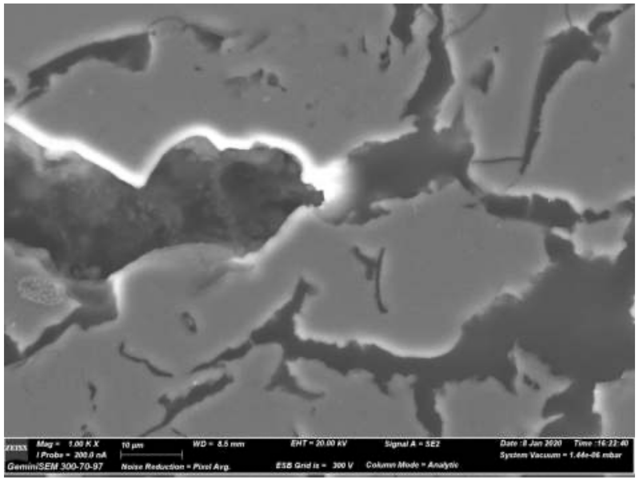

(e) Ceramic matrix (1000x)

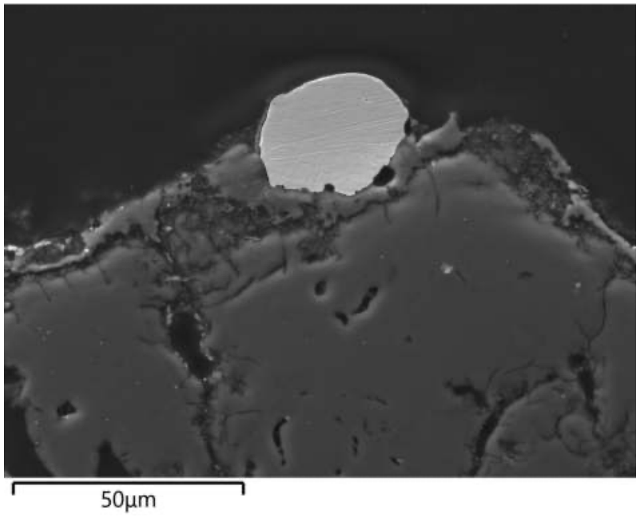

(f) Prill

Figure S3.2: CA200171

Table S3.2.1: CA200171 Bulk ceramic (all values reported as normalised wt%)

**CA200171**	**Al_2O_3**	**SiO_2**	**K_2O**	**CaO**	**TiO_2**	**FeO**	**SnO_2**	**Analytical total**
Analysis 1	25.8	67.7	3.9	0.3	0.7	1.5	n.d.	75.0
Analysis 2	22.7	70.5	4.2	n.d.	0.6	1.2	0.7	82.2
Analysis 3	27.1	66.8	4.0	n.d.	0.6	1.4	n.d.	81.2
Mean	25.1	67.9	4.0	$\leq$0.3	0.7	1.4	$\leq$0.7	80.0
Standard deviation (SD)	2.3	1.9	0.2	n/a	0.1	0.1	n/a	
Coefficient of variation (CV)	0.1	0.0	0.0	n/a	0.1	0.1	n/a	

Table S3.2.2: CA200171 Ceramic matrix (all values reported as normalised wt%)

**CA200171**	**MgO**	**Al_2O_3**	**SiO_2**	**P_2O_5**	**K_2O**	**CaO**	**TiO**	**FeO**	**Analytical total**
Analysis 1	n.d.	34.7	58.4	n.d.	4.2	n.d.	0.7	2.0	93.6
Analysis 2	n.d.	35.4	58.8	n.d.	3.8	n.d.	0.6	1.3	87.1
Analysis 3	0.5	32.2	57.6	0.5	5.1	0.6	0.8	2.6	101.3
Mean	$\leq$0.5	33.7	57.6	$\leq$0.5	4.3	$\leq$0.6	0.7	2.0	95.1
Standard deviation (SD)	n/a	1.7	0.6	n/a	0.6	n/a	0.1	0.6	
Coefficient of variation (CV)	n/a	0.0	0.0	n.a	0.1	n/a	0.1	0.3	

Table S3.2.3: CA200171 Inclusions (all values reported as normalised wt%)

**CA200171**	**Na_2O**	**Al_2O_3**	**SiO_2**	**K_2O**	**TiO**	**FeO**	**ZrO_2**	**Nb_2O_5**	**SnO_2**	**BaO**	**HfO_2**	**Analytical total**
Zircon	n.d.	0.7	31.6	0.4	n.d.	0.6	64.9	n.d.	n.d.	n.d.	1.9	98.3
Potassium feldspar	0.7	18.9	64.5	14.7	n.d.	n.d.	n.d.	n.d.	0.8	0.5	n.d.	115.3
Rutile	n.d.	0.7	1.4	0.1	96.2	0.7	n.d.	0.9	n.d.	n.d.	n.d.	75.9
**CA200171**	**Al_2O_3**	**SiO_2**	**P_2O_5**	**K_2O**	**FeO**	**Y_2O_3**	**Gd_2O_3**	**Dy_2O_3**	**Ho_2O_3**	**Er_2O_3**		**Analytical total**
Xenotime	10.7	11.9	27.7	1.0	0.5	38.2	1.5	4.9	0.9	2.7		105.3

Table S3.2.4: CA200171 Slag (all values reported as normalised wt%)

**CA200171**	**Na_2O**	**MgO**	**Al_2O_3**	**SiO_2**	**P_2O_5**	**K_2O**	**CaO**	**TiO_2**	**FeO**	**Analytical total**
Analysis 1	0.5	5.2	19.4	49.9	1.1	5.8	15.7	0.6	1.7	103.9
Analysis 2	0.3	5.1	12.1	45.7	2.5	2.3	29.9	0.3	1.7	112.0
Analysis 3	0.6	6.4	16.6	51.7	1.7	5.0	16.4	0.5	1.1	106.5
Mean	0.5	5.6	16.1	49.1	1.8	4.4	20.7	0.5	1.5	103.9
Standard deviation (SD)	0.1	0.7	3.7	3.1	0.7	1.8	8.0	0.1	0.4	
Coefficient of variation (CV)	0.2	0.1	0.2	0.1	0.4	0.4	0.4	0.3	0.2	

Table S3.2.5: CA200171 Prills (all values reported as normalised wt%)

**CA200171**	**O**	**Mg**	**Al**	**Si**	**K**	**Ca**	**Ti**	**Fe**	**Cu**	**As**	**Ag**	**Au**	**Analytical total**	**Size of prill**
Prill 1	1.4	n.d.	n.d.	n.d.	n.d.	n.d.	n.d.	3.0	1.3	n.d.	6.7	87.6	110.2	32µm
Prill 2	9.6	0.3	1.0	2.1	0.6	1.3	n.d.	0.4	n.d.	n.d.	n.d.	84.7	120.7	1µm
Prill 3	5.4	n.d.	3.6	3.4	0.4	n.d.	0.3	0.7	84.8	1.4	n.d.	n.d.	95.0	8µm

3 CA200172 (GZ10)

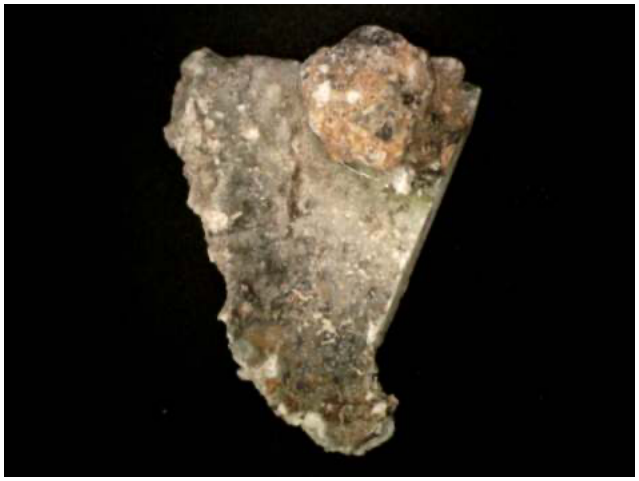

(a) Sample CA200172

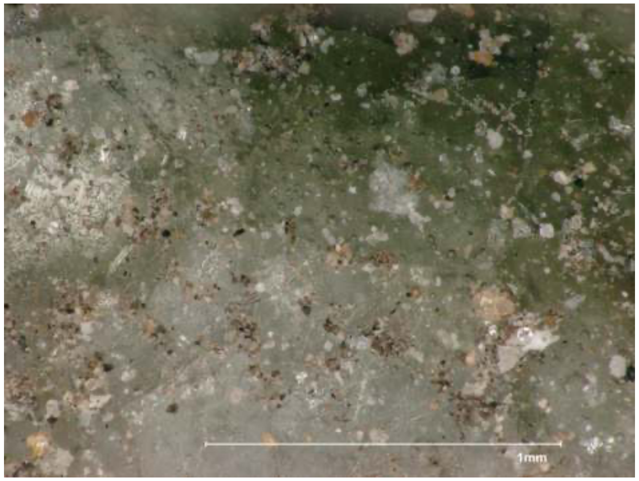

(b) Slag on the surface of CA200172)

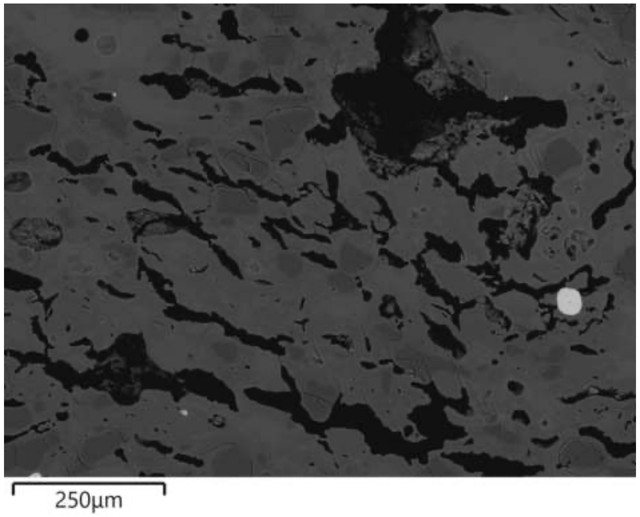

(c) Bulk ceramic (100x)

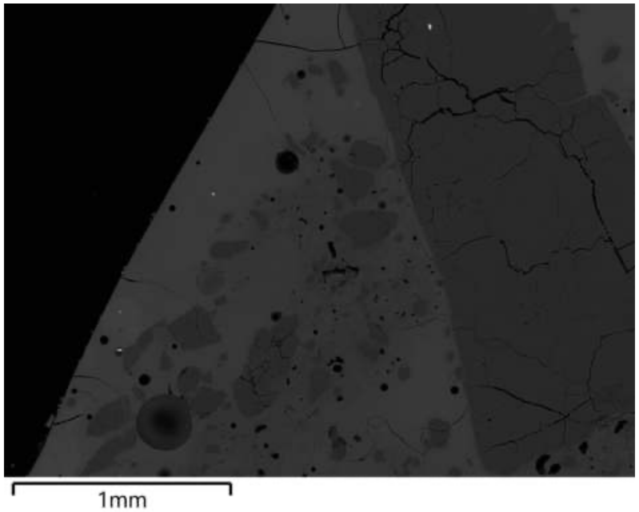

(d) Slag interphase (36x)

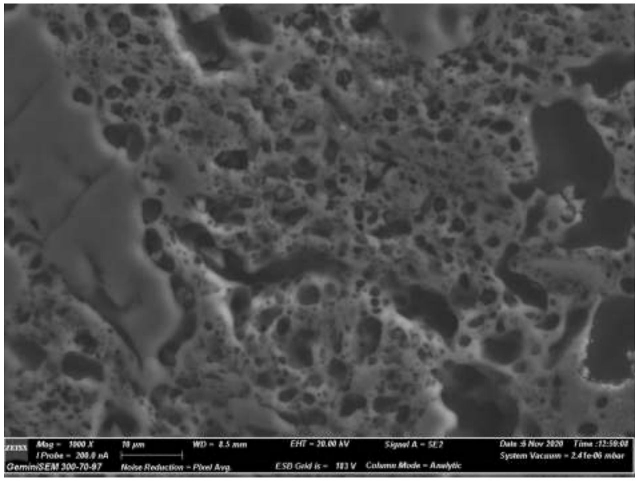

(e) Ceramic matrix (1000x)

Figure S3.3: CA200172

Table S3.3.1: CA200172 Bulk ceramic (all values reported as normalised wt%)

**CA200172**	**Al_2O_3**	**SiO_2**	**K_2O**	**TiO_2**	**FeO**	**Analytical total**
Analysis 1	24.9	68.5	4.4	0.5	1.7	80.2
Analysis 2	28.3	64.8	4.0	0.6	2.3	79.2
Analysis 3	27.7	65.1	4.0	0.6	2.6	80.1
Mean	26.9	66.1	4.1	0.6	2.2	79.8
Standard deviation (SD)	1.8	2.0	0.2	0.0	0.4	
Coefficient of variation (CV)	0.1	0.0	0.1	0.1	0.2	

Table S3.3.2: CA200172 Ceramic matrix (all values reported as normalised wt%)

**CA200172**	**Al_2O_3**	**SiO_2**	**K_2O**	**TiO**	**FeO**	**SnO_2**	**Analytical total**
Analysis 1	33.5	58.7	4.9	0.6	1.8	0.4	94.6
Analysis 2	33.5	58.0	4.9	0.6	2.3	0.8	79.2
Analysis 3	33.9	59.3	4.4	0.8	1.7	n.d.	101.6
Mean	33.6	58.7	4.7	0.7	1.9	0.4	92.0
Standard deviation (SD)	0.2	0.6	0.3	0.1	0.3	0.4	
Coefficient of variation (CV)	0.0	0.0	0.1	0.2	0.2	1.0	

Table S3.3.3: CA200172 Inclusions (all values reported as normalised wt%)

**CA200172**	**Na_2O**	**Al_2O_3**	**SiO_2**	**P_2O_5**	**K_2O**	**TiO**	**Cr_2O_3**	**FeO**	**Y_2O_3**	**ZrO_2**	**HfO_2**	**ThO_2**	**UO_3**	**Analytical total**
Zircon	n.d.	n.d.	31.5	n.d.	n.d.	n.d.	n.d.	n.d.	n.d.	67.0	1.5	n.d.	n.d.	90.4
Potassium feldspar	0.4	19.6	66.1	n.d.	13.0	n.d.	n.d.	0.9	n.d.	n.d.	n.d.	n.d.	n.d.	99.3
U-rich thorite	n.d.	n.d.	21.1	0.9	n.d.	n.d.	n.d.	n.d.	1.0	n.d.	n.d.	64.9	12.1	87.7
Chromite	n.d.	31.7	7.5	n.d.	0.6	1.2	51.6	7.4	n.d.	n.d.	n.d.	n.d.	n.d.	102.9
**CA200172**	**Al_2O_3**	**SiO_2**	**P_2O_5**	**K_2O**	**CaO**	**La_2O_3**	**Ce_2O_3**	**Pr_2O_3**	**Nd_2O_3**	**Sm_2O_3**	**Gd_2O_3**	**ThO_2**	**UO_3**	**Analytical total**
Monazite	1.0	3.1	30.4	0.5	1.0	14.0	27.5	2.2	11.6	1.9	1.7	4.2	1.0	64.2

Table S3.3.4: CA200172 Slag (all values reported as normalised wt%)

**CA200172**	**Na_2O**	**MgO**	**Al_2O_3**	**SiO_2**	**K_2O**	**CaO**	**TiO_2**	**FeO**	**Analytical total**
Analysis 1	0.3	1.6	23.8	55.2	6.1	11.6	0.5	1.1	95.4
Analysis 2	0.2	2.1	22.4	55.3	5.1	13.4	0.4	1.0	104.9
Analysis 3	0.4	1.4	23.6	54.4	5.3	13.0	0.7	1.3	105.8
Mean	0.3	1.7	23.3	55.0	5.5	12.7	0.5	1.1	102.0
Standard deviation (SD)	0.1	0.4	0.8	0.5	0.5	1.0	0.1	0.2	
Coefficient of variation (CV)	0.3	0.2	0.0	0.0	0.1	0.1	0.3	0.1	

4 CA200173 (GZ12)

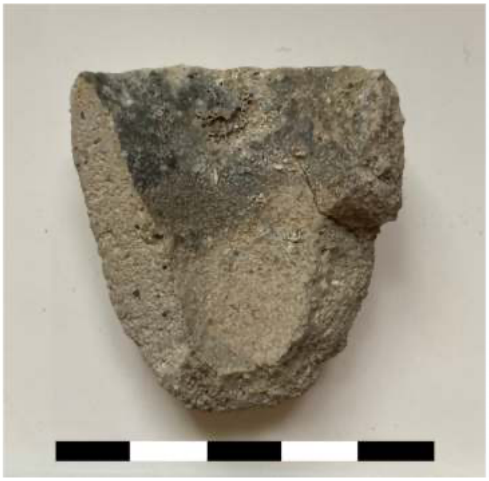

(a) Sample CA200173

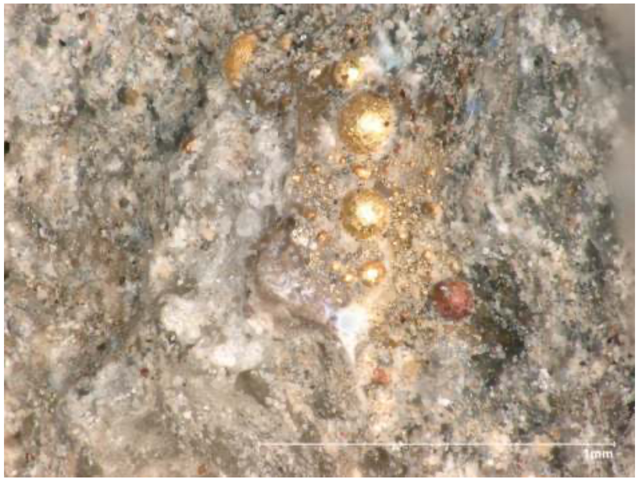

(b) Prills on surface of CA200173

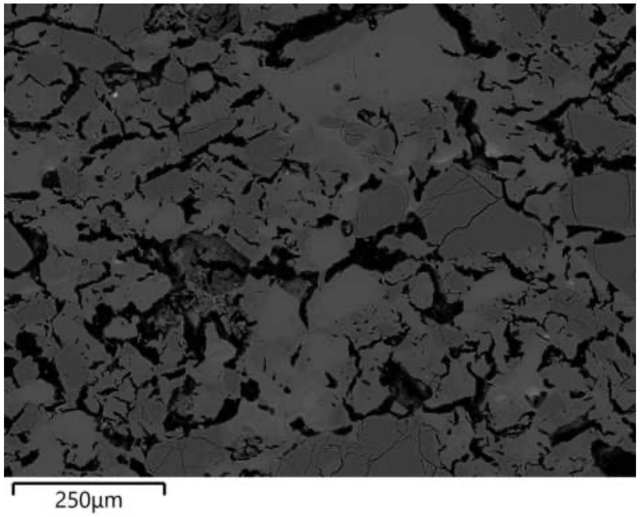

(c) Bulk ceramic (100x)

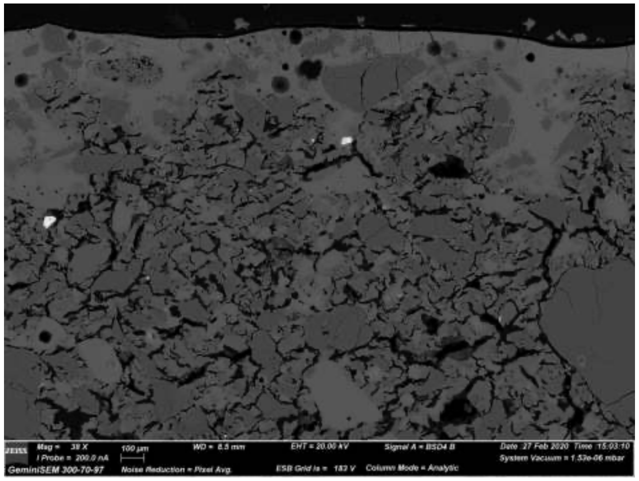

(d) Slag interphase (38x)

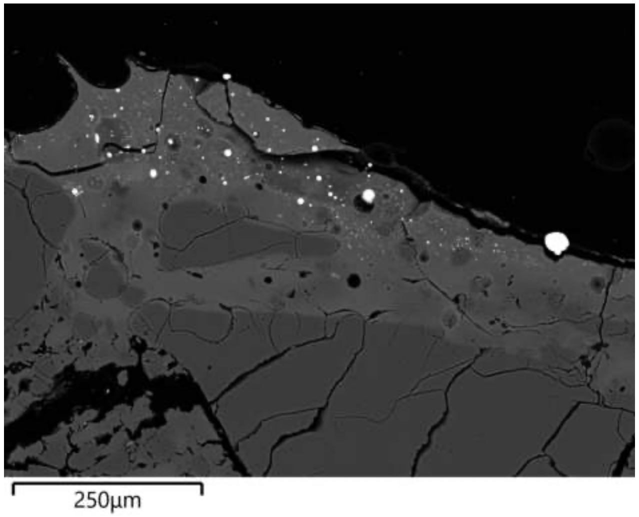

(e) Prills

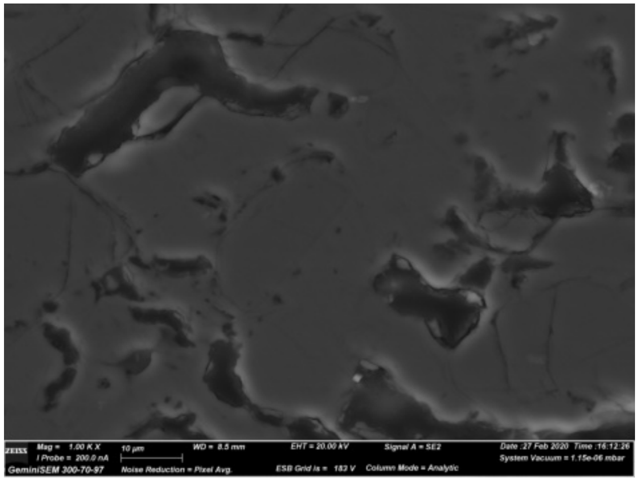

(f) Ceramic matrix (1000x)

Figure S3.4: CA200173.

Table S3.4.1: CA200173 Bulk ceramic (all values reported as normalised wt%)

**CA200173**	**Al_2O_3**	**SiO_2**	**K_2O**	**CaO**	**TiO_2**	**FeO**	**Analytical total**
Analysis 1	23.9	69.3	4.1	0.3	0.8	1.5	75.5
Analysis 2	23.7	70.5	3.8	n.d.	0.6	1.3	67.7
Analysis 3	22.1	72.5	3.7	n.d.	0.6	1.1	80.1
Mean	23.2	70.6	3.9	$\leq$0.3	0.7	1.3	74.6
Standard deviation (SD)	1.0	1.6	0.2	n/a	0.1	0.2	
Coefficient of variation (CV)	0.0	0.0	0.1	n/a	0.2	0.1	

Table S3.4.2: CA200173 Ceramic matrix (all values reported as normalised wt%)

**CA200173**	**Al_2O_3**	**SiO_2**	**K_2O**	**TiO**	**FeO**	**Analytical total**
Analysis 1	42.8	52.2	2.3	0.8	1.9	98.9
Analysis 2	37.7	56.6	3.5	0.8	1.5	101.7
Analysis 3	43.0	51.7	3.2	0.0	2.1	102.4
Mean	41.2	53.5	3.0	0.5	1.9	101.3
Standard deviation (SD)	3.0	2.7	0.6	0.4	0.3	
Coefficient of variation (CV)	0.1	0.1	0.2	0.9	0.2	

Table S3.4.3: CA200173 Inclusions (all values reported as normalised wt%)

**CA200173**	**Na_2O**	**MgO**	**Al_2O_3**	**SiO_2**	**K_2O**	**CaO**	**Sc_2O_3**	**TiO**	**MnO**	**FeO**	**ZrO_2**	**Nb_2O_5**	**BaO**	**HfO_2**	**ThO_2**	**Analytical total**
Zircon	n.d.	0.7	6.2	34.2	1.0	1.0	0.3	0.4	n.d.	1.3	51.1	n.d.	n.d.	0.9	2.9	66.6
Potassium feldspar	0.6	n.d.	19.7	64.6	14.5	n.d.	n.d.	n.d.	n.d.	0.4	n.d.	n.d.	0.3	n.d.	n.d.	78.4
Ilmenite	n.d.	n.d.	2.4	3.8	0.5	0.1	n.d.	45.4	3.5	43.8	n.d.	0.5	n.d.	n.d.	n.d.	99.7
Rutile	n.d.	n.d.	3.2	3.6	0.4	n.d.	n.d.	92.9	n.d.	n.d.	n.d.	n.d.	n.d.	n.d.	n.d.	101.6
**CA200173**	**Al_2O_3**	**SiO_2**	**P_2O_5**	**K_2O**	**FeO**	**As_2O_3**	**La_2O_3**	**Ce_2O_3**	**Pr_2O_3**	**Nd_2O_3**						
Monazite	1.8	3.3	28.1	0.3	0.4	3.4	21.6	31.3	2.2	7.6						72.1

Table S3.4.4: CA200173 Slag (all values reported as normalised wt%)

**CA200173**	**Na_2O**	**MgO**	**Al_2O_3**	**SiO_2**	**P_2O_5**	**K_2O**	**CaO**	**TiO_2**	**MnO**	**FeO**	**Analytical total**
Analysis 1	0.2	6.8	15.7	58.5	n.d.	5.9	11.4	0.5	0.2	0.7	95.7
Analysis 2	0.2	8.3	13.9	55.9	1.2	5.5	12.7	0.8	0.2	1.4	76.7
Analysis 3	n.d.	9.0	13.1	55.1	0.5	3.3	17.5	0.5	0.2	0.7	98.2
Mean	0.1	8.0	14.3	56.5	0.6	4.9	13.9	0.6	0.2	0.9	
Standard deviation (SD)	0.1	1.1	1.3	1.8	0.6	1.4	3.2	0.2	0.0	0.4	
Coefficient of variation (CV)	0.9	0.1	0.1	0.0	1.1	0.3	0.2	0.3	0.2	0.4	

Table S3.4.5: CA200173 Prills (all values reported as normalised wt%)

**CA200173**	**O**	**Si**	**Ca**	**Fe**	**Br**	**Ag**	**Au**	**Analytical total**	**Size of prill**
Analysis 1	2.9	n.d.	n.d.	0.5	n.d.	10.0	86.6	84.5	30µm
Analysis 2	2.4	n.d.	n.d.	1.1	n.d.	11.6	84.9	100.7	9µm
Analysis 3	5.2	1.0	0.7	n.d.	n.d.	10.7	82.4	90.4	5µm
Analysis 4	3.7	0.4	n.d.	n.d.	0.5	11.7	83.7	87.3	10µm
Analysis 5	2.8	n.d.	n.d.	0.4	n.d.	10.4	86.4	101.1	7µm
Analysis 6	3.6	n.d.	n.d.	0.9	n.d.	9.2	86.3	92.1	8µm
Mean	3.4	0.2	0.1	0.5	0.1	10.6	85.0	94.2	
Standard deviation (SD)	1.0	0.4	0.3	0.5	0.2	0.9	1.7		
Coefficient of variation (CV)	0.3	1.7	2.4	1.0	2.4	0.1	0.0		

5 CA200174 (GZ1)

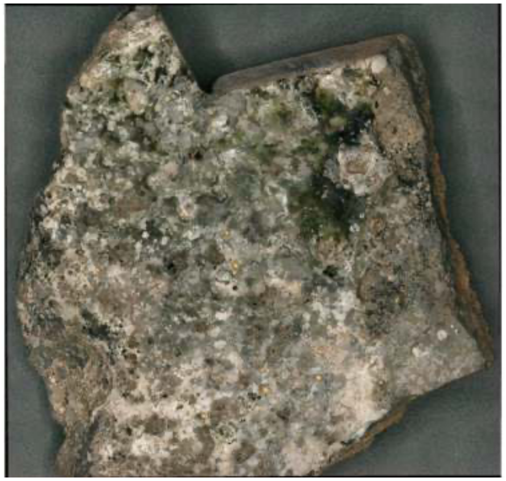

(a) Sample CA200174

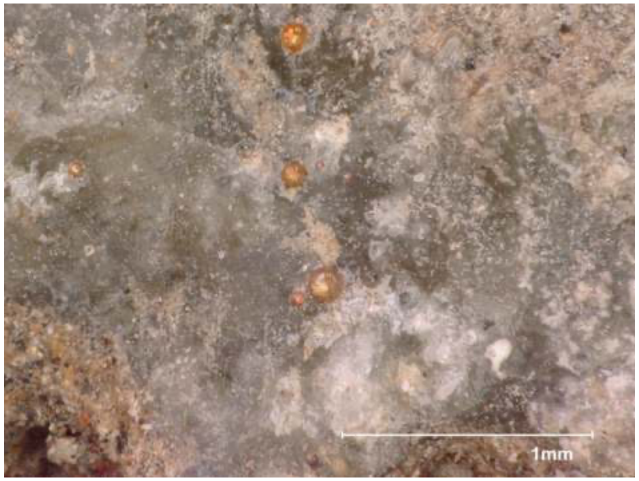

(b) Prills on the surface of CA200174

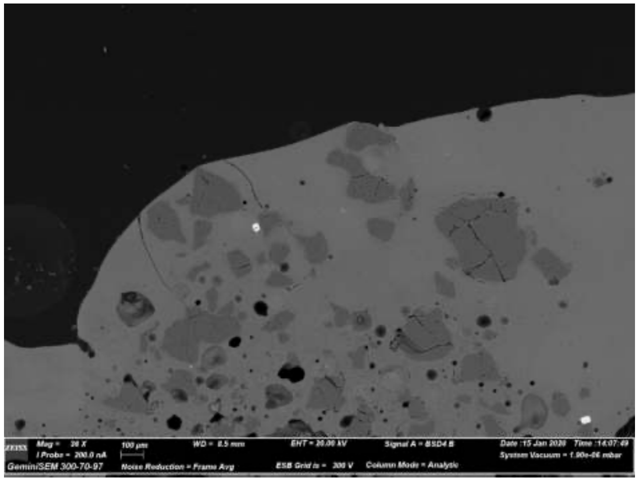

(c) Interphase between ceramic and slag (36x)

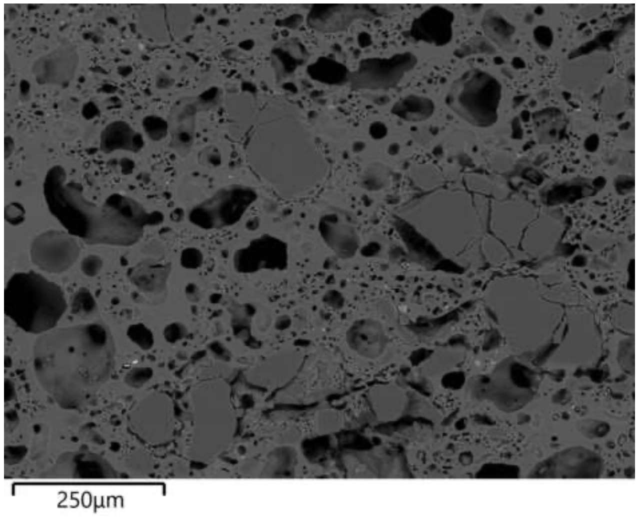

(d) Bulk ceramic (100x)

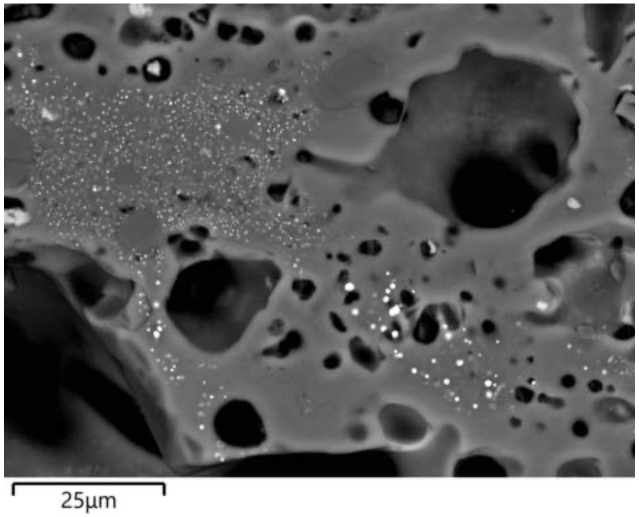

(e) Fe reducing within the body

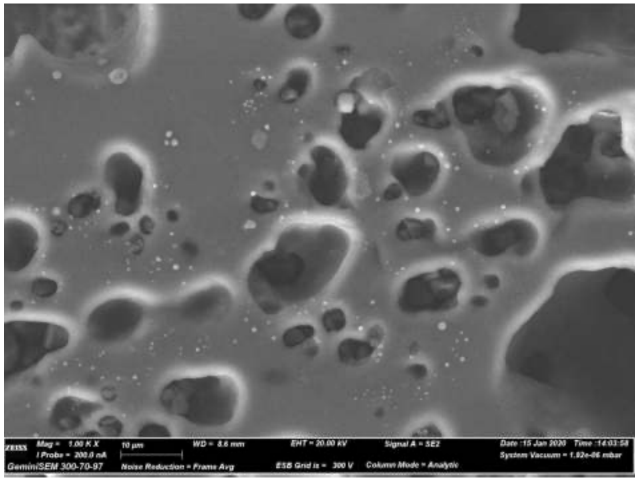

(f) Ceramic matrix (1000x)

Figure S3.5: CA200174

Table S3.5.1: CA200174 Bulk ceramic (all values reported as normalised wt%)

**CA200174**	**Na_2O**	**MgO**	**Al_2O_3**	**SiO_2**	**K_2O**	**CaO**	**TiO_2**	**FeO**	**BaO**	**Analytical total**
Analysis 1	1.2	n.d.	19.8	68.6	4.2	0.8	0.9	4.5	n.d.	67.8
Analysis 2	1.2	0.2	21.2	65.0	5.5	0.9	0.8	4.9	0.3	70.0
Analysis 3	1.5	n.d.	21.6	63.3	4.7	0.9	1.2	6.8	n.d.	65.4
Mean	1.3	$\leq$0.2	20.8	65.4	4.8	0.9	1.0	5.4	$\leq$0.3	67.9
Standard deviation (SD)	0.2	0.1	1.0	2.7	0.7	0.1	0.2	1.2	n/a	
Coefficient of variation (CV)	0.1	0.6	0.0	0.0	0.1	0.1	0.2	0.2	n/a	

Table S3.5.2: CA200174 Ceramic matrix (all values reported as normalised wt%)

**CA200174**	**Na_2O**	**MgO**	**Al_2O_3**	**SiO_2**	**K_2O**	**CaO**	**TiO_2**	**FeO**	**Analytical total**
Analysis 1	1.3	n.d.	29.2	56.6	4.7	0.7	1.7	5.7	87.7
Analysis 2	1.2	0.3	30.1	54.8	5.4	0.9	1.2	6.2	79.0
Analysis 3	1.2	0.2	30.6	55.1	4.6	0.5	2.2	5.5	83.2
Mean	1.3	0.2	30.0	55.5	4.9	0.7	1.7	5.8	83.3
Standard deviation (SD)	0.1	0.1	0.7	0.9	0.4	0.2	0.5	0.3	
Coefficient of variation (CV)	0.1	0.9	0.0	0.0	0.1	0.3	0.3	0.1	

Table S3.5.3: CA200174 Inclusions (all values reported as normalised wt%)

**CA200174**	**Na_2O**	**MgO**	**Al_2O_3**	**SiO_2**	**P_2O_5**	**K_2O**	**CaO**	**TiO**	**V_2O_5**	**FeO**	**ZrO_2**	**Nb_2O_5**	**BaO**	**HfO_2**	**Ce_2O_3**	**Analytical total**
Zircon	n.d.	n.d.	n.d.	30.7	n.d.	n.d.	n.d.	n.d.	n.d.	n.d.	66.1	1.9	n.d.	1.3	n.d.	108.8
Zircon	n.d.	n.d.	2.7	32.4	n.d.	n.d.	0.5	n.d.	n.d.	3.8	60.0	n.d.	n.d.	n.d.	0.7	125.5
Potassium feldspar	3.1	n.d.	19.2	65.5	n.d.	9.1	0.9	n.d.	n.d.	1.4	n.d.	n.d.	0.9	n.d.	n.d.	105.5
Alumina silicate	0.8	n.d.	28.4	43.6	n.d.	1.3	13.1	0.8	n.d.	12.0	n.d.	n.d.	n.d.	n.d.	n.d.	93.0
Ti-rich mineral	n.d.	1.4	11.5	7.3	0.3	0.8	n.d.	61.2	0.7	16.2	n.d.	0.6	n.d.	n.d.	n.d.	104.5

Table S3.5.4: CA200174 Slag (all values reported as normalised wt%)

**CA200174**	**Na_2O**	**MgO**	**Al_2O_3**	**SiO_2**	**P_2O_5**	**K_2O**	**CaO**	**TiO_2**	**FeO**	**Analytical total**
Analysis 1	1.2	7.2	17.4	52.1	1.0	5.5	11.5	0.8	3.3	101.4
Analysis 2	1.1	8.1	16.7	51.4	1.0	5.1	12.4	0.8	3.4	98.8
Analysis 3	1.2	7.9	15.7	54.0	0.7	4.8	11.4	0.7	3.6	93.2
Mean	1.2	7.8	16.6	52.5	0.9	5.1	11.8	0.7	3.4	100.1
Standard deviation (SD)	0.1	0.5	0.9	1.4	0.2	0.3	0.6	0.0	0.1	
Coefficient of variation (CV)	0.0	0.1	0.1	0.0	0.2	0.1	0.1	0.0	0.0	

Table S3.5.5: CA200174 Prills (all values reported as normalised wt%)

**CA200174**	**O**	**Al**	**Si**	**K**	**Ca**	**Fe**	**Ag**	**Au**	**Analytical total**	**Size of prill**
Analysis 1	1.3	0.0	0.0	0.0	0.0	0.6	0.0	98.1	107.9	3µm
Analysis 2	13.4	0.6	2.1	0.6	0.3	0.4	0.6	81.8	132.9	1µm

6 CA200175 (GZ6)

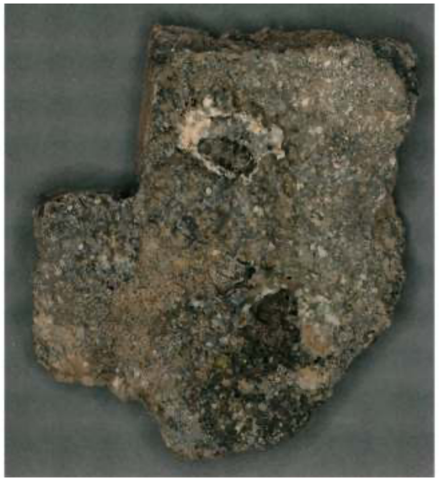

(a) Sample CA200175

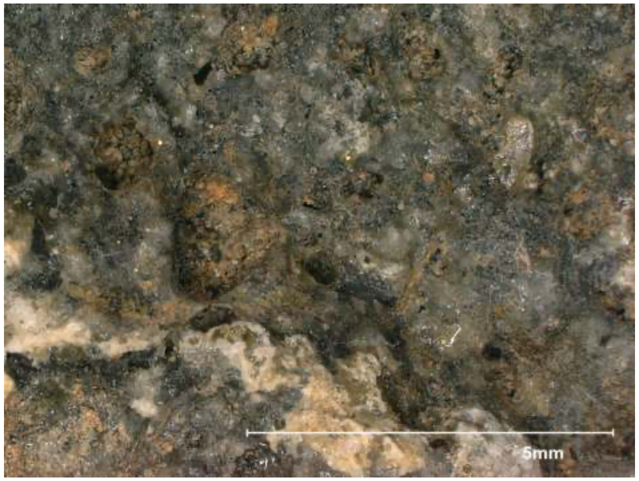

(b) Prills and slagging on the surface of CA200175)

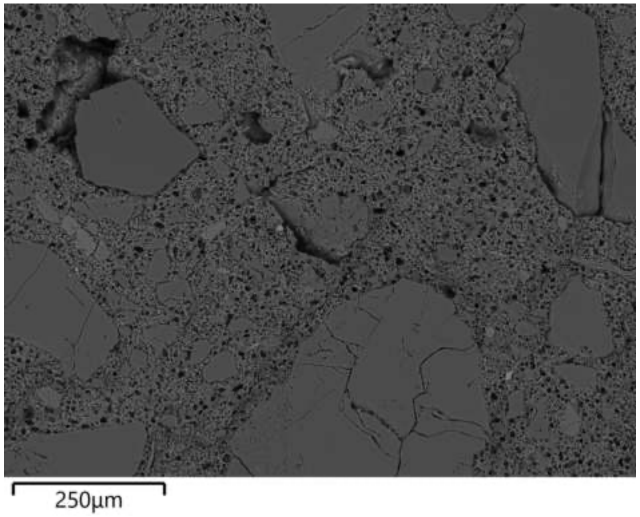

(c) Bulk ceramic (100x)

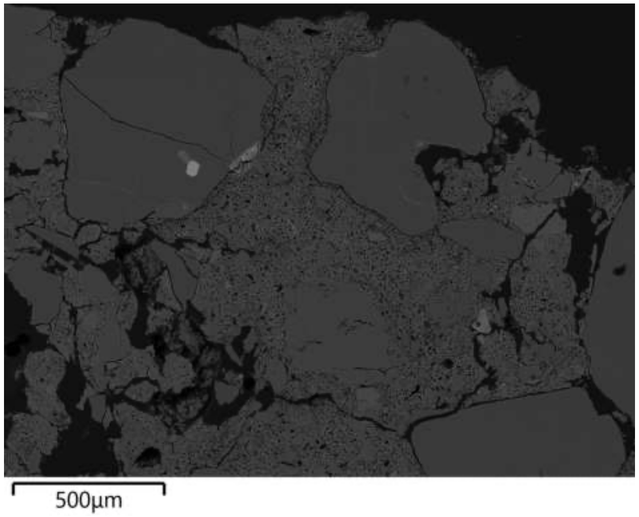

(d) CA200175 Surface without slag

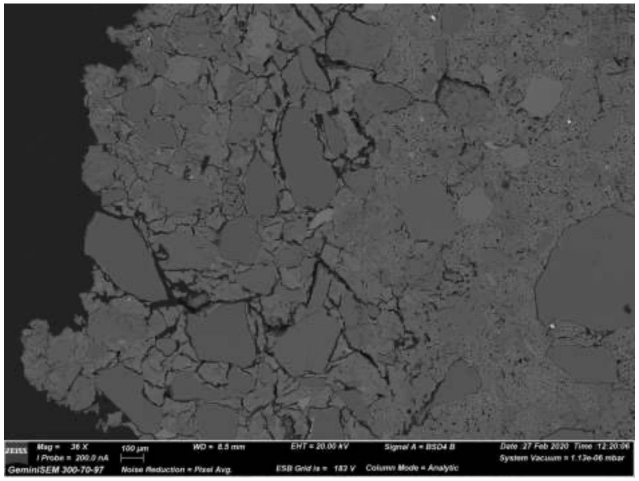

(e) Inclusion-rich area

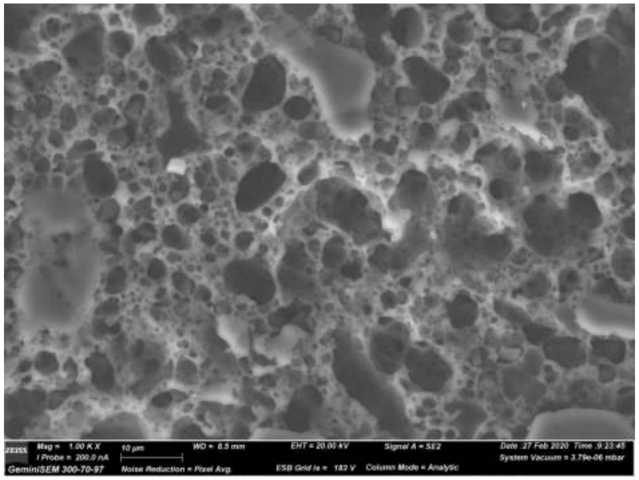

(f) Ceramic matrix (1000x)

Figure S3.6: CA200175

Table S3.6.1: CA200175 Bulk ceramic (all values reported as normalised wt%)

**CA200175**	**Na_2O**	**MgO**	**Al_2O_3**	**SiO_2**	**K_2O**	**CaO**	**TiO_2**	**FeO**	**Analytical total**
Analysis 1	1.9	1.2	21.9	62.4	2.9	2.6	0.7	6.4	78.0
Analysis 2	2.7	1.2	21.6	63.4	2.5	2.8	0.5	5.3	82.7
Analysis 3	2.1	1.5	21.6	61.7	3.1	2.8	0.6	6.6	70.6
Mean	2.2	1.3	21.7	62.5	2.9	2.7	0.6	6.1	75.3
Standard deviation (SD)	0.4	0.2	0.2	0.9	0.3	0.1	0.1	0.7	
Coefficient of variation (CV)	0.2	0.1	0.0	0.0	0.1	0.0	0.2	0.1	

Table S3.6.2: CA200175 Ceramic matrix (all values reported as normalised wt%)

**CA200175**	**Na_2O**	**MgO**	**Al_2O_3**	**SiO_2**	**P_2O_5**	**K_2O**	**CaO**	**TiO**	**MnO**	**FeO**	**Analytical total**
Analysis 1	1.2	2.0	30.3	49.4	0.4	3.7	3.2	0.9	n.d.	8.9	73.4
Analysis 2	1.4	1.8	29.9	48.1	0.4	4.3	2.9	0.9	n.d.	10.3	64.0
Analysis 3	1.3	1.8	27.8	51.9	0.4	3.8	2.9	0.8	0.2	9.1	70.5
Mean	1.3	1.9	29.3	49.7	0.4	3.9	3.0	0.9	$\leq$0.2	9.4	69.4
Standard deviation (SD)	0.1	0.1	1.3	1.9	0.0	0.3	0.2	0.1	n/a	0.8	
Coefficient of variation (CV)	0.1	0.1	0.1	0.0	0.1	0.1	0.1	0.1	n/a	0.1	

Table S3.6.3: CA200175 Inclusions (all values reported as normalised wt%)

**CA200175**	**Na_2O**	**MgO**	**Al_2O_3**	**SiO_2**	**P_2O_5**	**K_2O**	**CaO**	**TiO**	**V_2O_5**	**Cr_2O_3**	**MnO**	**FeO**	**Nb_2O_5**	**BaO**	**Analytical total**
Iron oxide	n.d.	n.d.	0.3	0.6	0.3	n.d.	0.1	n.d.	0.2	n.d.	n.d.	98.6	n.d.	n.d.	100.5
Potassium feldspar	0.8	n.d.	19.2	62.0	n.d.	14.6	n.d.	n.d.	n.d.	n.d.	n.d.	0.2	n.d.	3.2	75.8
Soda feldspar	8.2	n.d.	24.4	61.5	n.d.	0.1	5.6	n.d.	n.d.	n.d.	n.d.	0.2	n.d.	n.d.	103.6
Alumina silicate	n.d.	n.d.	25.2	37.1	n.d.	0.1	22.3	n.d.	n.d.	n.d.	n.d.	15.3	n.d.	n.d.	87.3
Mg-rich mineral	0.7	17.4	4.5	53.2	n.d.	0.2	12.5	0.2	n.d.	0.1	0.3	10.9	n.d.	n.d.	88.1
Ilmenite	n.d.	n.d.	n.d.	0.2	n.d.	n.d.	0.1	50.1	0.5	n.d.	4.5	44.5	n.d.	n.d.	78.7
Rutile	n.d.	n.d.	0.9	0.9	n.d.	0.1	0.2	95.1	n.d.	n.d.	n.d.	2.4	0.4	n.d.	102.3

7 CA200176 (GZ8)

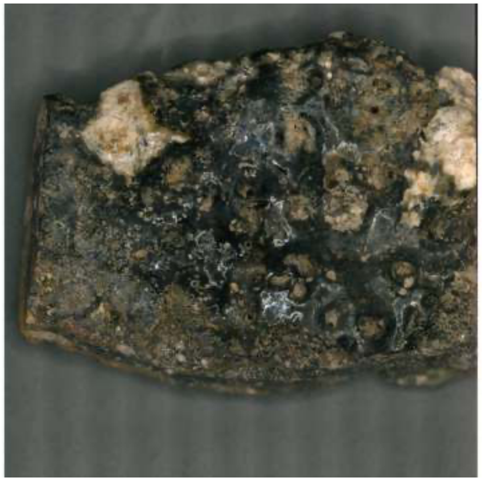

(a) Sample CA200176

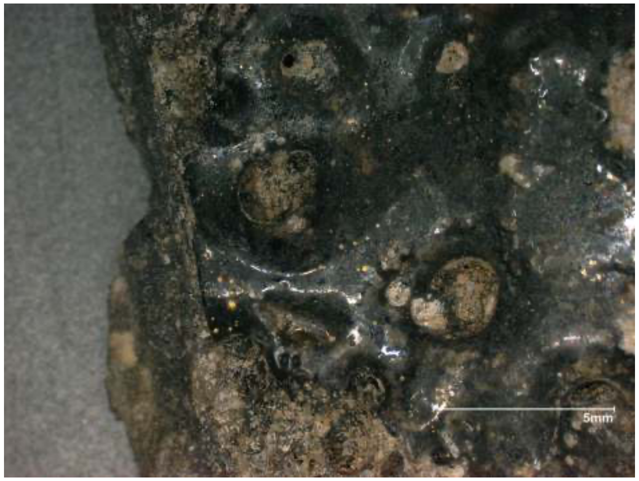

(b) Prills on the surface of CA200176)

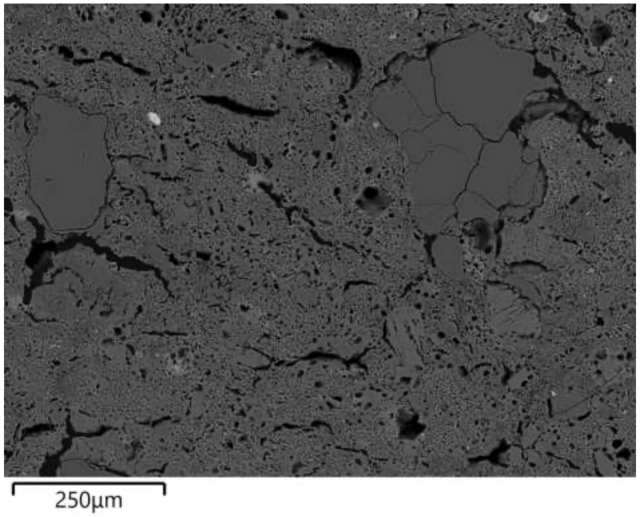

(c) Bulk ceramic (100x)

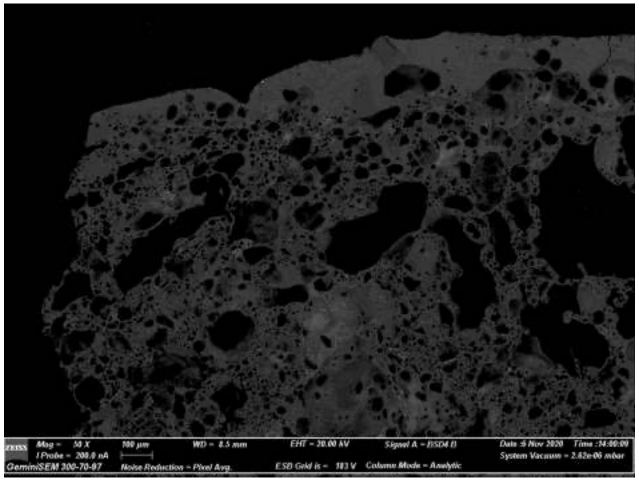

(d) Slag interphase (36x), organic temper?

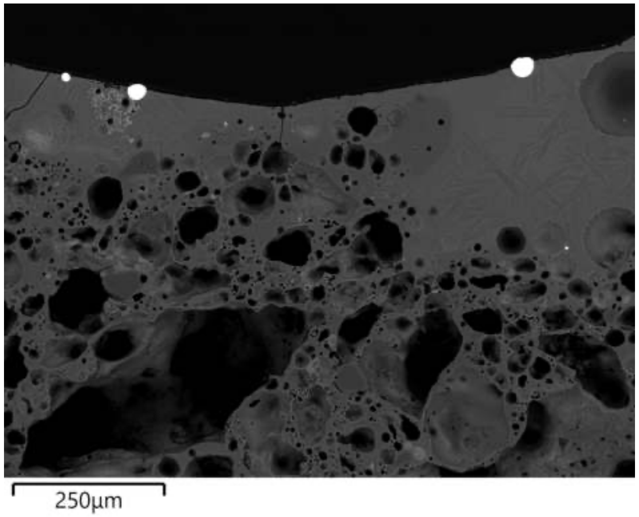

(e) Prills, organic temper?

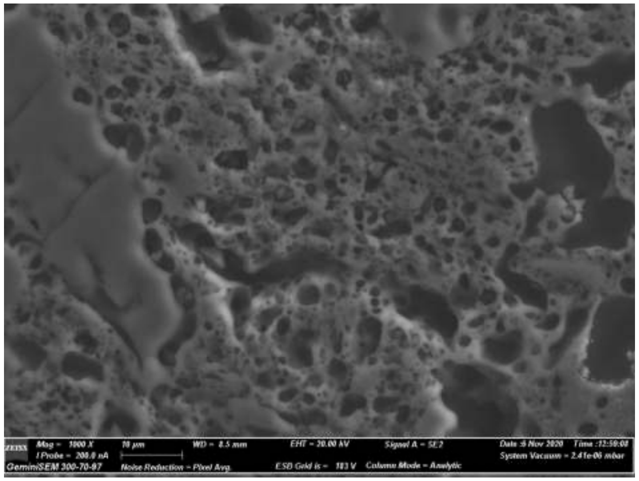

(f) Ceramic matrix (1000x)

Figure S3.7: CA200176

Table S3.7.1: CA200176 Bulk ceramic (all values reported as normalised wt%)

**CA200176**	**Na_2O**	**MgO**	**Al_2O_3**	**SiO_2**	**K_2O**	**CaO**	**TiO_2**	**FeO**	**Analytical total**
Analysis 1	1.4	1.3	27.4	55.2	2.5	0.5	1.2	10.5	62.1
Analysis 2	1.5	0.9	26.8	55.0	2.8	0.4	1.2	11.3	62.1
Analysis 3	2.9	0.9	26.2	54.9	1.3	0.5	1.1	12.2	54.7
Mean	2.0	1.1	26.8	55.0	2.2	0.5	1.1	11.3	58.5
Standard deviation (SD)	0.8	0.2	0.6	0.2	0.8	0.0	0.1	0.9	
Coefficient of variation (CV)	0.4	0.2	0.0	0.0	0.4	0.0	0.1	0.1	

Table S3.7.2: CA200176 Ceramic matrix (all values reported as normalised wt%)

**CA200176**	**Na_2O**	**MgO**	**Al_2O_3**	**SiO_2**	**K_2O**	**CaO**	**TiO**	**FeO**	**Analytical total**
Analysis 1	0.7	1.5	33.2	50.9	1.8	0.6	0.5	10.8	77.0
Analysis 2	1.1	1.2	30.9	52.0	2.2	0.4	1.0	11.3	60.4
Analysis 3	1.1	1.1	30.8	51.3	1.8	0.5	1.3	12.1	62.2
Mean	0.9	1.3	31.6	51.4	1.9	0.5	0.9	11.4	66.6
Standard deviation (SD)	0.3	0.2	1.4	0.6	0.3	0.1	0.4	0.7	
Coefficient of variation (CV)	0.3	0.2	0.0	0.0	0.1	0.2	0.4	0.1	

Table S3.7.3: CA200176 Inclusions (all values reported as normalised wt%)

**CA200176**	**Al_2O_3**	**SiO_2**	**P_2O_5**	**CaO**	**V_2O_5**	**FeO**	**Y_2O_3**	**ZrO_2**	**Ag_2O**	**HfO_2**	**Ce_2O_3**	**UO_3**	**Analytical total**
Zircon	1.8	27.9	n.d.	0.5	n.d.	2.5	3.2	56.0	2.3	1.6	2.1	2.1	71.1
**CA200176**	**Na_2O**	**MgO**	**Al_2O_3**	**SiO_2**	**K_2O**	**CaO**	**TiO**	**V_2O_5**	**Cr_2O_3**	**MnO**	**FeO**		**Analytical total**
Potassium feldspar	1.6	n.d.	18.9	64.8	14.6	n.d.	n.d.	n.d.	n.d.	n.d.	0.2		105.1
Soda feldspar	10.2	n.d.	21.8	66.7	0.9	n.d.	n.d.	n.d.	n.d.	n.d.	0.4		97.5
Ilmenite	n.d.	n.d.	0.7	n.d.	n.d.	n.d.	57.5	0.5	n.d.	1.0	40.2		93.9
Fe-rich mineral	n.d.	1.2	21.0	16.2	0.5	0.8	0.6	0.3	0.4	n.d.	59.0		66.7
**CA200176**	**Al_2O_3**	**SiO_2**	**P_2O_5**	**CaO**	**V_2O_5**	**FeO**	**Y_2O_3**	**Ce_2O_3**	**Nd_2O_3**	**Gd_2O_3**	**ThO_2**		**Analytical total**
Thorite	4.8	19.2	3.6	n.d.	1.1	3.6	1.6	2.0	1.2	0.6	62.3		109.9

Table S3.7.4: CA200176 Slag (all values reported as normalised wt%)

**CA200176**	**Na_2O**	**MgO**	**Al_2O_3**	**SiO_2**	**P_2O_5**	**K_2O**	**CaO**	**TiO_2**	**FeO**	**Analytical total**
Analysis 1	1.0	3.4	21.6	43.8	4.2	2.3	15.5	0.6	7.5	103.8
Analysis 2	1.0	4.2	21.7	45.4	3.8	3.8	12.4	0.8	6.8	111.9
Analysis 3	1.0	4.7	20.7	44.8	2.2	4.2	14.8	1.0	6.6	101.3
Mean	1.0	4.1	21.3	44.7	3.4	3.4	14.2	0.8	7.0	105.7
Standard deviation (SD)	0.0	0.6	0.6	0.8	1.1	1.0	1.6	0.2	0.5	
Coefficient of variation (CV)	0.0	0.2	0.0	0.0	0.3	0.3	0.1	0.3	0.1	

Table S3.7.5: CA200176 Prills (all values reported as normalised wt%)

**CA200176**	**O**	**Fe**	**Ag**	**Au**	**Analytical total**	**Size of prill**
Prill 1	2.7	1.2	1.3	94.9	106.9	5µm
Prill 2	3.7	0.9	1.3	94.1	112.9	3µm
Prill 3	2.1	n.d.	1.4	96.4	100.1	15µm
Prill 4	2.0	n.d.	1.7	96.3	101.9	30µm

8 CA200177 (GZ11)

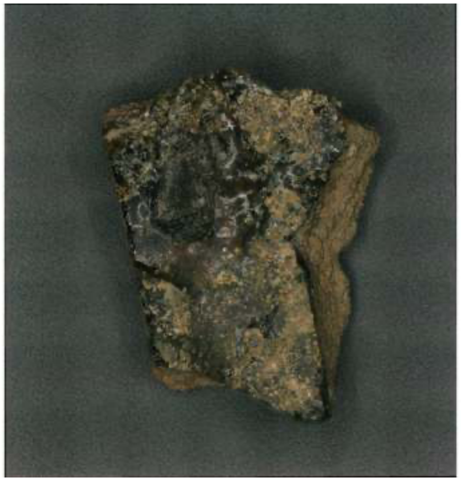

(a) Sample CA200177

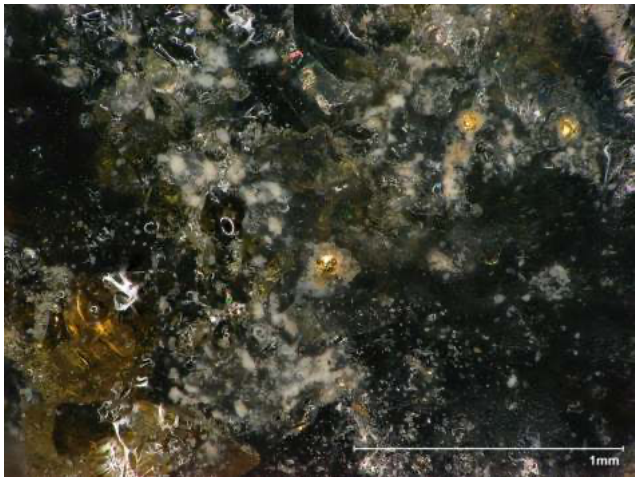

(b) Prills on surface of CA200177

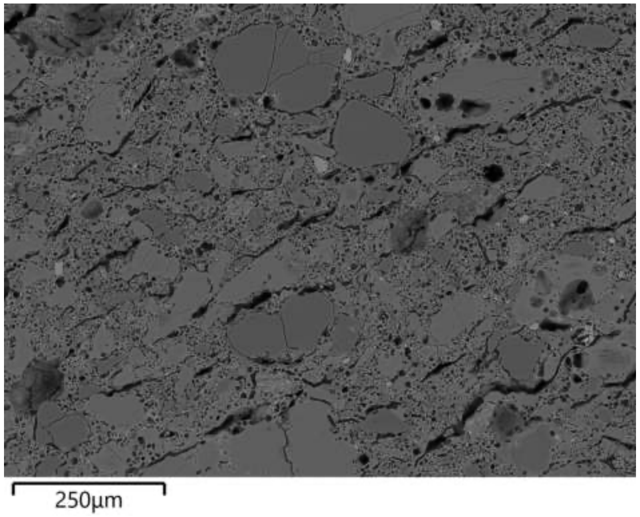

(c) Bulk ceramic (100x)

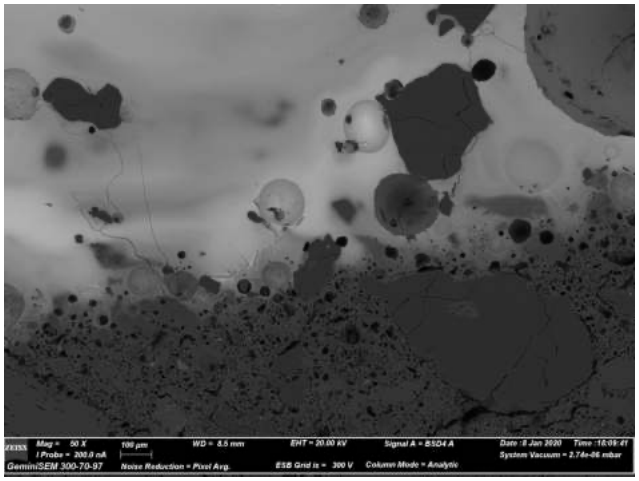

(d) Slag interphase (50x)

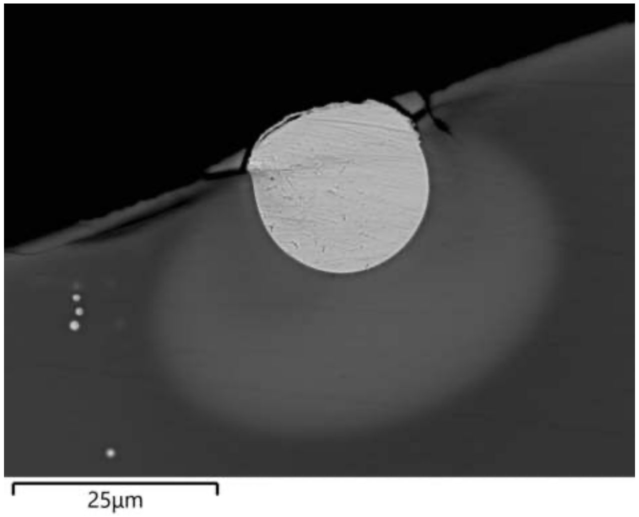

(e) Prill with halo in lead glass

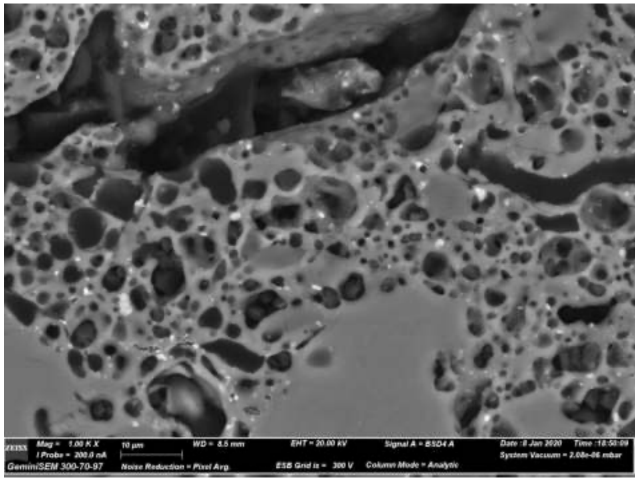

(f) Ceramic matrix (1000x)

Figure S3.8: CA200177

Table S3.8.1: CA200177 Bulk ceramic (all values reported as normalised wt%)

**CA200177**	**Na_2O**	**MgO**	**Al_2O_3**	**SiO_2**	**K_2O**	**CaO**	**TiO_2**	**FeO**	**PbO**	**Analytical total**
Analysis 1	0.9	0.3	19.0	67.3	6.2	0.5	0.7	4.4	0.6	76.7
Analysis 2	1.2	n.d.	20.5	64.7	6.0	1.0	0.9	4.8	0.8	91.0
Analysis 3	1.4	0.3	21.7	60.9	6.2	1.2	1.1	6.5	0.7	79.0
Mean	1.1	0.2	20.4	64.3	6.1	0.9	0.9	5.3	0.7	82.3
Standard deviation (SD)	0.2	0.2	1.3	3.2	0.2	0.4	0.2	1.1	0.1	
Coefficient of variation (CV)	0.2	0.9	0.1	0.1	0.0	0.4	0.2	0.2	0.2	

Table S3.8.2: CA200177 Ceramic matrix (all values reported as normalised wt%)

**CA200177**	**Na_2O**	**MgO**	**Al_2O_3**	**SiO_2**	**P_2O_5**	**K_2O**	**CaO**	**TiO**	**FeO**	**PbO**	**Analytical total**
Analysis 1	1.3	0.7	31.4	48.7	0.4	5.4	0.8	1.8	8.6	0.9	80.9
Analysis 2	1.6	0.6	30.1	49.6	0.5	5.4	0.9	2.0	8.4	0.9	83.4
Analysis 3	1.2	0.5	31.4	49.2	0.6	4.2	1.7	1.8	8.5	0.8	93.9
Mean	1.4	0.6	31.0	49.2	0.5	5.0	1.2	1.9	8.5	0.8	86.1
Standard deviation (SD)	0.2	0.1	0.7	0.5	0.1	0.7	0.5	0.1	0.1	0.1	
Coefficient of variation (CV)	0.1	0.1	0.0	0.0	0.2	0.1	0.4	0.0	0.0	0.1	

Table S3.8.3: CA200177 Inclusions (all values reported as normalised wt%)

**CA200177**	**Na_2O**	**MgO**	**Al_2O_3**	**SiO_2**	**P_2O_5**	**K_2O**	**CaO**	**TiO**	**V_2O_5**	**FeO**	**Nb_2O_5**	**In_2O_3**	**BaO**	**PbO**	**Analytical total**
Potassium feldspar	1.2	n.d.	18.8	64.4	n.d.	15.0	n.d.	n.d.	n.d.	n.d.	n.d.	n.d.	0.6	n.d.	109.0
Soda feldspar	8.4	n.d.	22.5	62.5	n.d.	1.7	2.2	n.d.	n.d.	2.8	n.d.	n.d.	n.d.	n.d.	78.6
Ti-rich mineral	n.d.	12.9	14.0	1.0	1.2	0.5	n.d.	57.8	0.8	11.2	n.d.	n.d.	n.d.	0.5	70.3
Rutile	n.d.	n.d.	3.7	0.7	n.d.	n.d.	n.d.	94.7	n.d.	0.5	0.3	0.2	n.d.	n.d.	111.3
Fe-rich mineral	0.9	1.0	18.6	29.2	1.2	2.9	0.7	0.4	0.2	44.5	n.d.	n.d.	n.d.	0.5	100.4

Table S3.8.4: CA200177 Slag (all values reported as normalised wt%)

**CA200177**	**Na_2O**	**MgO**	**Al_2O_3**	**SiO_2**	**P_2O_5**	**K_2O**	**CaO**	**TiO_2**	**FeO**	**PbO**	**Analytical total**
Analysis 1	0.7	1.2	11.3	38.1	0.8	2.1	9.2	0.6	2.9	33.0	105.7
Analysis 2	0.8	1.8	11.5	41.2	1.8	2.9	9.7	0.7	2.8	26.9	89.2
Mean	0.7	1.5	11.4	39.6	1.3	2.5	9.5	0.7	2.9	29.9	97.4
Standard deviation (SD)	0.1	0.4	0.1	2.2	0.7	0.6	0.3	0.1	0.1	4.3	
Coefficient of variation (CV)	0.1	0.3	0.0	0.1	0.5	0.2	0.0	0.1	0.0	0.1	

Table S3.8.5: CA200177 Prills (all values reported as normalised wt%)

**CA200177**	**O**	**Cu**	**Ag**	**Au**	**Pb**	**Bi**	**Total**	**Analytical total**	**Size of prill**
Prill 1	1.3	1.5	8.5	88.7	n.d.	n.d.	100.0	97.5	20µm
Prill 2	1.9	12.2	17.5	61.0	5.7	1.6	100.0	102.7	7µm

Table S3.8.6: CA200177 Smaller prills next to prill 1 (all values reported as normalised wt%)

**CA200177**	**O**	**Mg**	**Al**	**Si**	**K**	**Ar**	**Ca**	**Ti**	**Fe**	**Cu**	**As**	**Ag**	**Bi**	**Th**	**Analytical total**
Analysis 1	11.2	n.d.	n.d.	0.8	2.3	n.d.	n.d.	n.d.	0.3	0.6	21.9	7.8	55.1	n.d.	115.1
Analysis 2	14.1	0.2	0.2	2.0	6.5	n.d.	0.7	0.8	0.6	0.6	36.7	19.3	18.4	n.d.	111.7
Analysis 3	14.5	0.2	2.1	6.6	0.9	n.d.	0.9	0.8	0.7	20.3	19.6	33.6	n.d.	n.d.	107.3
Analysis 4	18.4	n.d.	n.d.	1.9	5.3	1.1	0.7	0.6	0.5	5.0	n.d.	17.2	41.0	8.3	128.3

Table S3.8.7: CA200177 Halo around prill 1 (all values reported as normalised wt%)

**CA200177**	**Na_2O**	**MgO**	**Al_2O_3**	**SiO_2**	**K_2O**	**CaO**	**TiO_2**	**FeO**	**CuO**	**PbO**	**Bi2O3**	**Analytical total**
Halo	0.4	0.5	6.1	22.2	1.0	2.0	0.5	2.3	0.8	53.8	10.3	101.3
Glass near halo	0.6	0.6	8.9	32.7	2.5	2.5	0.5	2.1	n.d.	49.7	n.d.	98.8

9 CA200178 (GZ7)

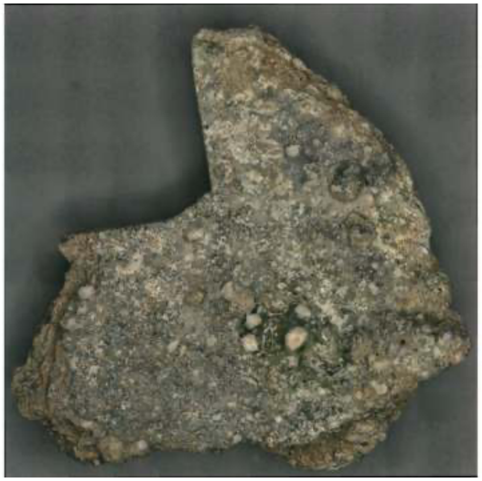

(a) Sample CA200178

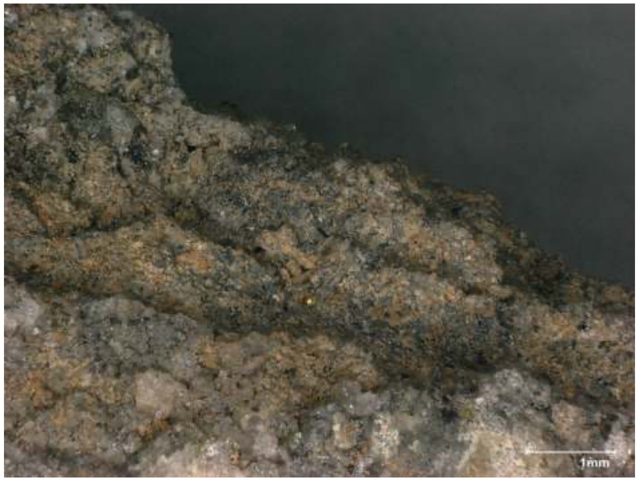

(b) Prill in CA200178)

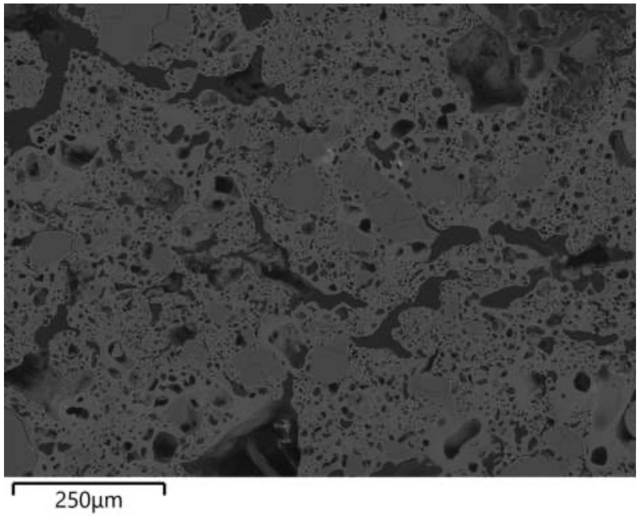

(c) Bulk ceramic (100x)

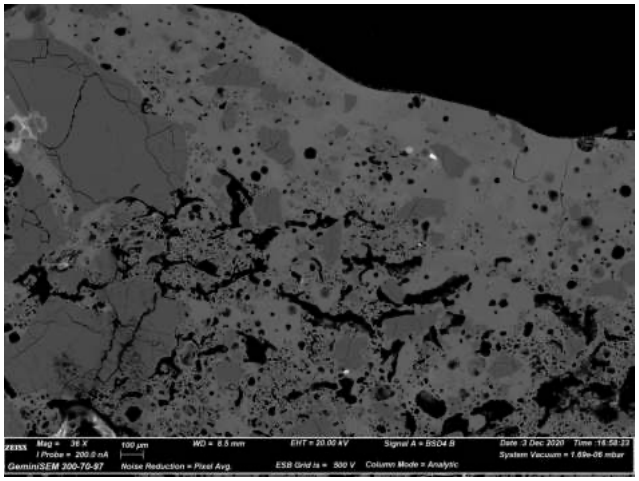

(d) Slag interphase (36x)

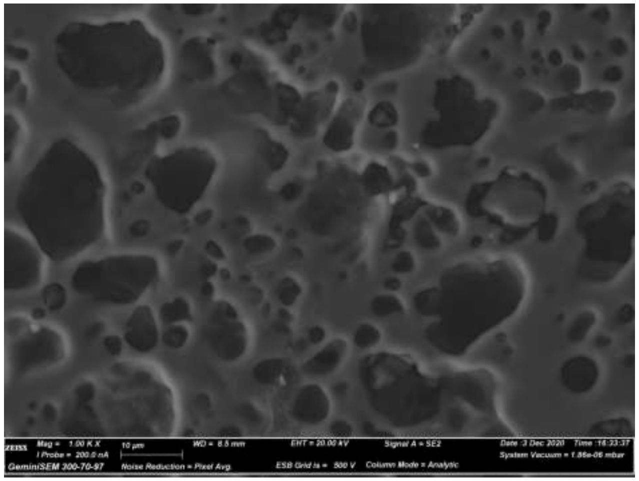

(e) Ceramic matrix (1000x)

Figure S3.9: CA200178

Table S3.9.1: CA200178 Bulk ceramic (all values reported as normalised wt%)

**CA200178**	**Na_2O**	**MgO**	**Al_2O_3**	**SiO_2**	**K_2O**	**CaO**	**TiO_2**	**FeO**	**Analytical total**
Analysis 1	n.d.	n.d.	26.7	63.5	3.6	n.d.	0.6	5.7	77.5
Analysis 2	n.d.	n.d.	27.7	62.4	2.7	0.3	0.7	6.2	70.6
Analysis 3	0.2	0.2	26.3	62.1	4.1	n.d.	0.7	6.3	81.1
Mean	≤0.2	≤0.2	26.8	62.4	3.4	≤0.3	0.7	6.0	76.8
Standard deviation (SD)	0.1	0.1	0.7	0.7	0.7	0.1	0.1	0.3	
Coefficient of variation (CV)	0.6	0.6	0.0	0.0	0.2	0.6	0.1	0.1	

Table S3.9.2: CA200178 Ceramic matrix (all values reported as normalised wt%)

**CA200178**	**Al_2O_3**	**SiO_2**	**K_2O**	**TiO**	**FeO**	**Analytical total**
Analysis 1	36.9	51.9	2.1	0.7	8.3	91.6
Analysis 2	35.3	53.6	2.5	0.7	8.0	71.3
Analysis 3	35.6	54.1	2.2	0.8	7.3	88.6
Mean	36.0	53.2	2.3	0.7	7.9	83.8
Standard deviation (SD)	0.9	1.2	0.2	0.1	0.5	
Coefficient of variation (CV)	0.0	0.0	0.1	0.1	0.1	

Table S3.3: Inclusions in furnace wall CA200178 (all values reported as normalised wt%)

**CA200178**	**Na_2O**	**Al_2O_3**	**SiO_2**	**P_2O_5**	**K_2O**	**TiO**	**V_2O_5**	**Cr_2O_3**	**MnO**	**FeO**	**ZrO_2**	**Ag_2O**	**HfO_2**	**UO_3**	**Analytical total**
Zircon	n.d.	3.2	33.7	n.d.	0.2	n.d.	n.d.	n.d.	n.d.	4.0	55.2	0.8	1.5	1.3	94.9
Potassium feldspar	0.4	18.8	65.1	n.d.	14.2	n.d.	n.d.	n.d.	n.d.	1.6	n.d.	n.d.	n.d.	n.d.	91.5
Ti-rich mineral	n.d.	2.5	1.1	0.3	n.d.	84.0	0.8	0.6	0.8	9.9	n.d.	n.d.	n.d.	n.d.	105.3
Ilmenite	n.d	1.0	n.d.	n.d.	n.d.	54.3	n.d.	n.d.	2.9	41.8	n.d.	n.d.	n.d.	n.d.	103.8
**CA200178**	**Al_2O_3**	**SiO_2**	**P_2O_5**	**CaO**	**V_2O_5**	**FeO**	**Y_2O_3**	**La_2O_3**	**Ce_2O_3**	**Pr_2O_3**	**Nd_2O_3**	**Gd_2O_3**	**ThO_2**		**Analytical total**
Monazite	n.d.	2.0	28.4	0.5	n.d.	n.d.	n.d.	17.2	32.0	2.6	8.5	n.d.	8.8		93.4

Table S3.4: CA200178 Slag (all values reported as normalised wt%)

**CA200178**	**Na_2O**	**MgO**	**Al_2O_3**	**SiO_2**	**P_2O_5**	**K_2O**	**CaO**	**TiO_2**	**MnO**	**FeO**	**Analytical total**
Analysis 1	0.5	4.3	24.2	53.0	1.0	5.8	5.0	0.9	0.2	5.1	101.3
Analysis 2	0.8	2.7	25.6	53.9	0.6	8.1	4.0	0.5	n.d.	3.8	96.3
Analysis 3	0.6	2.6	27.7	52.6	1.0	5.9	4.6	0.7	n.d.	4.4	92.0
Mean	0.6	3.2	25.8	53.1	0.9	6.6	4.5	0.7	$\leq$0.2	4.4	96.6
Standard deviation (SD)	0.1	1.0	1.8	0.7	0.2	1.3	0.5	0.2	n/a	0.6	
Coefficient of variation (CV)	0.2	0.3	0.1	0.0	0.2	0.2	0.1	0.3	n/a	0.1	

Table S3.5: CA200178 Prill that could be contamination (all values reported as normalised wt%)

**CA200178**	**O**	**Al**	**Si**	**Fe**	**Cu**	**Sn**	**Au**	**Analytical total**	**Size of prill**
Prill 1	4.1	0.3	0.3	0.4	79.3	15.6	n.d.	92.0	6µm

10 CA200179 (GZ9)

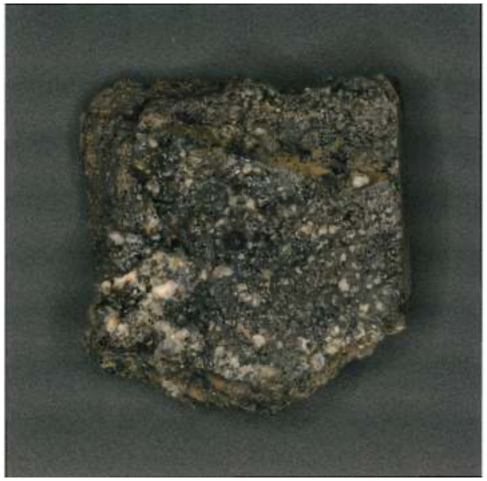

(a) Sample CA200179

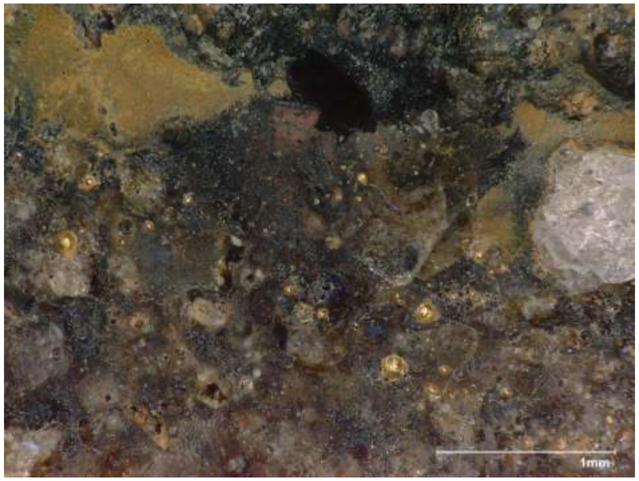

(b) Prills and virtification on the surface of sample CA200179

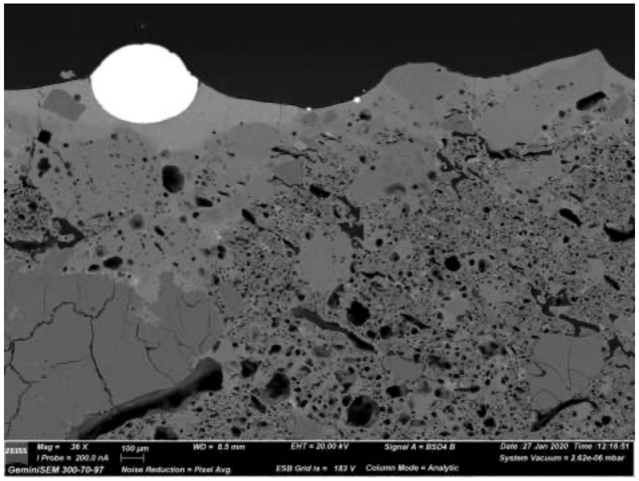

(c) Interphase between ceramic and slag (BSE, 36x)

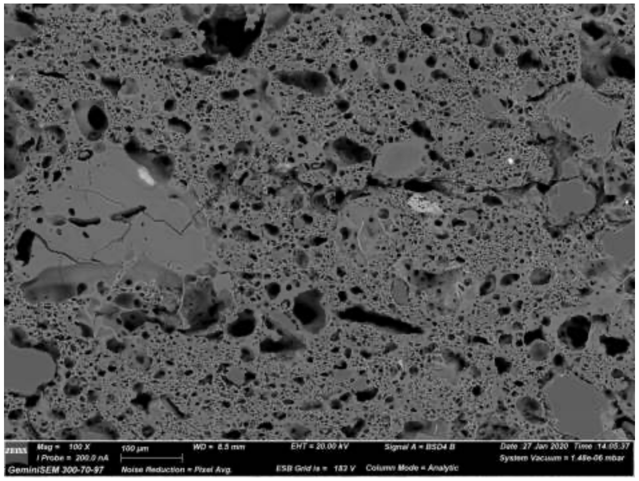

(d) Bulk ceramic (BSE, 100x)

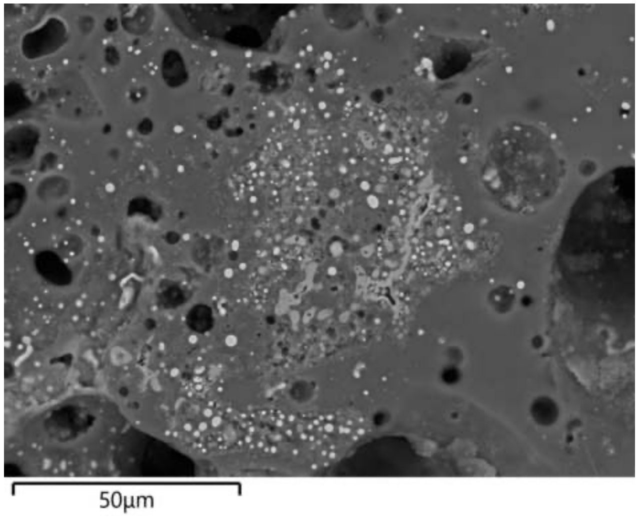

(e) Fe-rich mineral reducing in CA200179 (BSE, 750x)

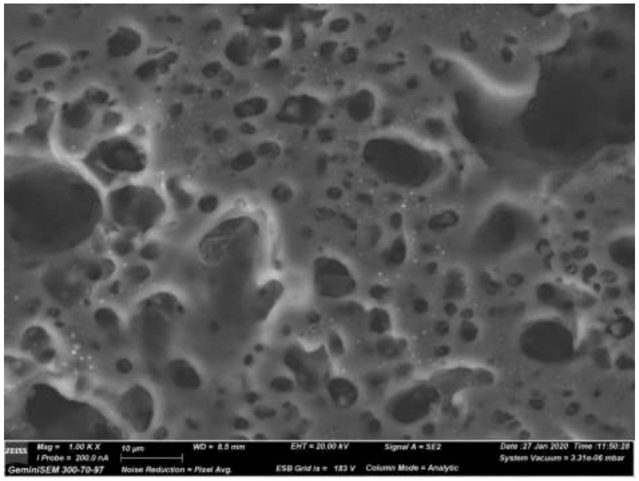

(f) Ceramic matrix (SE, 1000x)

Figure S3.10: CA200179

Table S3.10.1: CA200179 Bulk ceramic (all values reported as normalised wt%)

CA200179	Na_2O	MgO	Al_2O_3	SiO_2	K_2O	CaO	TiO_2	FeO	Analytical total
Analysis 1	1.5	0.9	22.6	65.1	1.8	0.8	0.6	6.6	71.4
Analysis 2	1.5	0.9	24.0	63.0	1.8	0.9	0.7	7.1	77.5
Analysis 3	1.5	0.8	26.6	60.2	2.0	1.0	0.7	7.1	73.6
Mean	1.5	0.9	24.4	62.8	1.9	0.9	0.7	6.9	73.0
Standard deviation (SD)	0.0	0.1	2.0	2.5	0.1	0.1	0.1	0.3	
Coefficient of variation (CV)	0.0	0.1	0.1	0.0	0.1	0.1	0.1	0.0	

Table S3.10.2: CA200179 Ceramic matrix (all values reported as normalised wt%)

CA200179	Na_2O	MgO	Al_2O_3	SiO_2	K_2O	CaO	TiO_2	FeO	Analytical total
Analysis 1	1.7	1.1	33.2	52.3	1.5	0.7	0.8	8.6	86.6
Analysis 2	0.8	1.1	32.5	53.7	1.5	0.5	0.8	9.0	73.3
Analysis 3	1.2	1.0	33.7	52.5	2.0	0.7	0.8	8.1	82.7
Mean	1.3	1.1	33.1	52.9	1.7	0.6	0.8	8.6	80.8
Standard deviation (SD)	0.5	0.1	0.6	0.7	0.3	0.1	0.0	0.4	
Coefficient of variation (CV)	0.4	0.1	0.0	0.0	0.2	0.2	0.0	0.0	

Table S3.10.3: CA200179 Inclusions (all values reported as normalised wt%)

CA200179	Na_2O	MgO	Al_2O_3	SiO_2	P_2O_5	K_2O	CaO	TiO	V_2O_5	Cr_2O_3	MnO	FeO	ZrO_2	HfO_2	Analytical total
Zircon	n.d.	n.d.	31.3	n.d.	n.d.	n.d.	n.d.	n.d.	n.d.	n.d.	n.d.	n.d.	67.4	1.3	81.3
Potassium feldspar	1.0	n.d.	19.8	62.2	n.d.	14.5	n.d.	n.d.	n.d.	n.d.	n.d.	2.6	n.d.	n.d.	89.4
Ilmenite	n.d.	n.d.	2.4	3.1	n.d.	n.d.	n.d.	52.0	n.d.	0.3	1.9	40.3	n.d.	n.d.	101.6
Mineral with Na/Ca	6.5	n.d.	23.1	62.8	n.d.	0.9	5.0	n.d.	n.d.	n.d.	n.d.	1.7	n.d.	n.d.	99.7
Chromite	n.d.	n.d.	7.3	0.3	0.4	n.d.	0.3	n.d.	n.d.	55.4	n.d.	36.3	n.d.	n.d.	106.6
Fe-rich mineral	0.7	1.7	24.9	22.4	0.3	0.7	0.8	0.4	0.2	0.2	n.d.	47.6	n.d.	n.d.	104.2

Table S3.10.4: CA200179 Prills (all values reported as normalised wt%)

CA200179	O	Al	Si	Fe	Ag	Au	Analytical total	Size of prill
Analysis 1	1.8	0.0	0.0	0.0	9.9	88.3	115.0	4500µm
Analysis 2	1.9	0.0	0.0	0.0	8.9	89.3	101.1	25µm
Analysis 3	3.3	0.0	0.0	0.4	12.0	84.3	80.0	20µm
Analysis 4	21.3	2.9	2.8	1.7	71.2	0.0	133.3	1µm

11 CA200180 (GZ3)

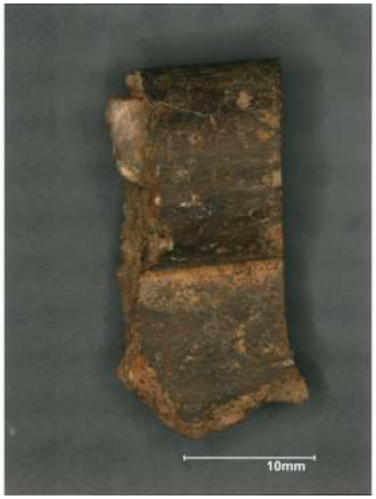

(a) Sample CA200180

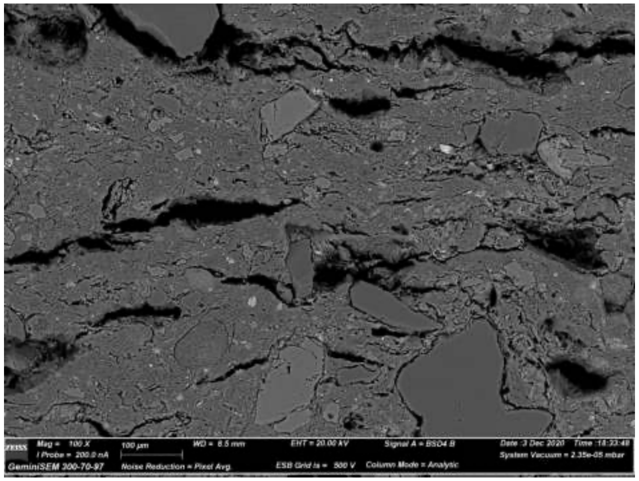

(b) Bulk ceramic (BSE, 100x)

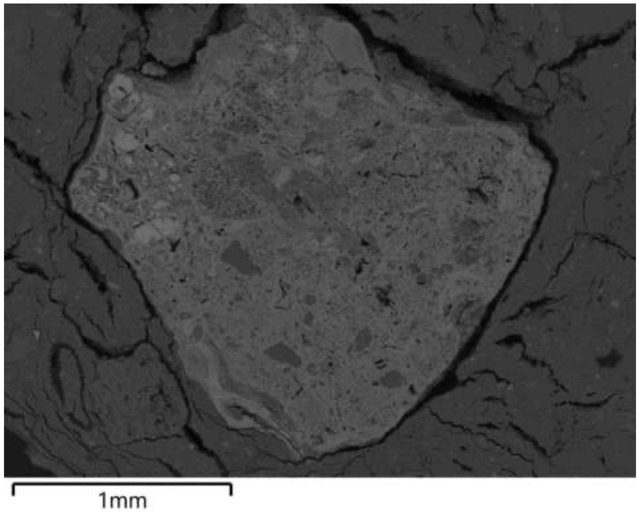

(c) Iron-rich clay pellet (36x)

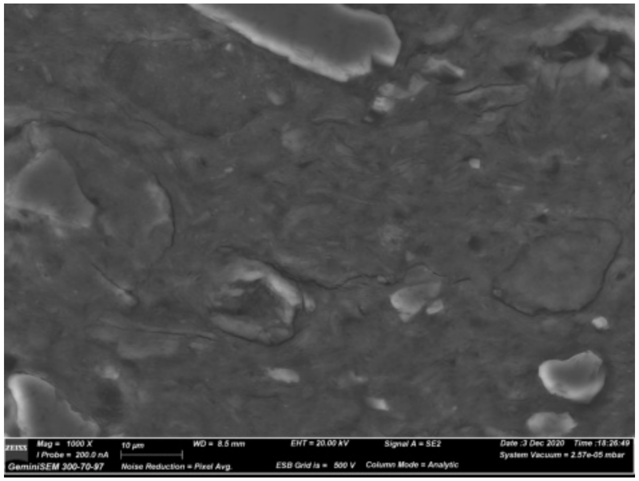

(d) Ceramic matrix (SE, 1000x)

Figure S3.11: CA200180

Table S3.11.1: CA200180 Bulk ceramic (all values reported as normalised wt%)

**CA200180**	**Na_2O**	**MgO**	**Al_2O_3**	**SiO_2**	**P_2O_5**	**K_2O**	**CaO**	**TiO_2**	**FeO**	**Analytical total**
Analysis 1	0.8	0.7	24.2	61.9	0.7	2.2	1.8	1.0	6.6	70.6
Analysis 2	0.7	0.8	24.7	62.8	0.6	2.0	1.7	0.6	6.2	66.8
Analysis 3	1.3	0.8	25.3	61.8	n.d.	1.9	1.7	0.6	6.6	92.0
Mean	0.9	0.7	24.7	62.2	0.4	2.1	1.7	0.7	6.5	75.9
Standard deviation (SD)	0.3	0.0	0.6	0.6	0.4	0.2	0.1	0.3	0.2	
Coefficient of variation (CV)	0.3	0.1	0.0	0.0	0.9	0.1	0.0	0.3	0.0	

Table S3.11.2: CA200180 Ceramic matrix (all values reported as normalised wt%)

**CA200180**	**MgO**	**Al_2O_3**	**SiO_2**	**P_2O_5**	**K_2O**	**CaO**	**TiO**	**FeO**	**Analytical total**
Analysis 1	1.0	32.3	52.7	0.9	1.5	2.2	0.5	9.0	83.2
Analysis 2	1.2	31.6	55.3	0.7	0.7	2.2	0.8	7.4	87.8
Analysis 3	0.8	31.1	53.5	0.9	2.7	2.2	0.6	8.3	83.6
Mean	1.0	31.7	53.8	0.8	1.6	2.2	0.6	8.2	84.9
Standard deviation (SD)	0.2	0.6	1.4	0.1	1.0	0.0	0.1	0.8	
Coefficient of variation (CV)	0.2	0.0	0.0	0.1	0.6	0.0	0.2	0.1	

Table S3.11.3: CA200180 Inclusions (all values reported as normalised wt%)

**CA200180**	**Na_2O**	**MgO**	**Al_2O_3**	**SiO_2**	**P_2O_5**	**K_2O**	**CaO**	**TiO**	**V_2O_5**	**MnO**	**FeO**	**ZrO_2**	**Ag_2O**	**HfO_2**	**Analytical total**
Zircon	n.d.	n.d.	1.4	30.1	n.d.	n.d.	1.0	n.d.	n.d.	n.d.	2.7	61.9	1.2	1.8	75.5
Ilmenite	n.d.	n.d.	0.8	0.6	n.d.	n.d.	n.d.	56.1	n.d.	1.0	41.6	n.d.	n.d.	n.d.	84.0
Ti-rich mineral	n.d.	n.d.	0.9	1.1	n.d.	n.d.	0.3	91.4	1.3	n.d.	5.0	n.d.	n.d.	n.d.	74.1
Potassium feldspar	0.4	n.d.	18.8	64.7	n.d.	16.0	n.d.	n.d.	n.d.	n.d.	n.d.	n.d.	n.d.	n.d.	98.6
Soda feldspar	9.2	0.2	21.9	64.4	0.4	0.4	1.1	0.3	n.d.	n.d.	2.1	n.d.	n.d.	n.d.	63.0

12 CA200181 (GZ5)

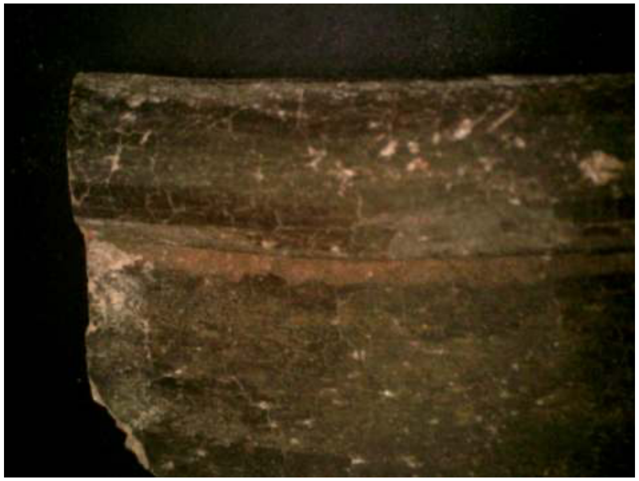

(a) Sample CA200181

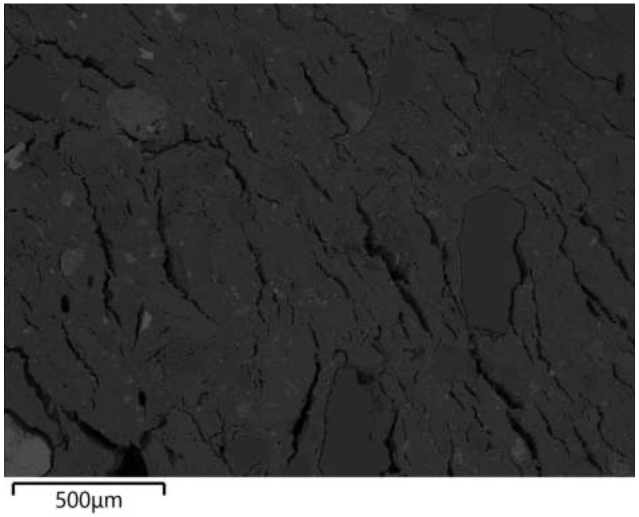

(b) Bulk ceramic (100x)

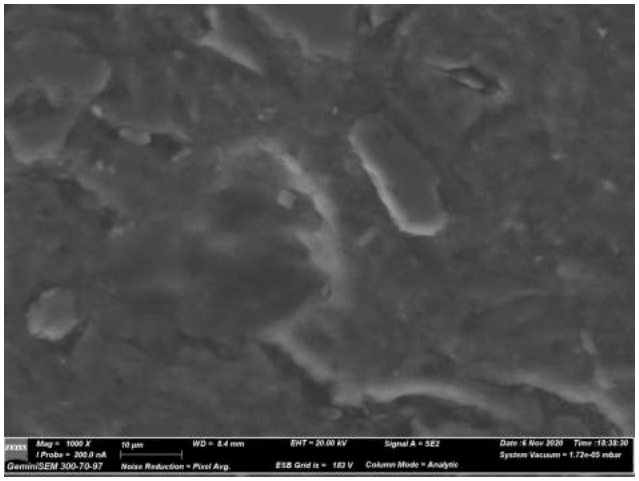

(c) Ceramic matrix (1000x)

Figure S3.12: CA200181

Table S3.12.1: CA200181 Bulk ceramic (all values reported as normalised wt%)

**CA200181**	**Na_2O**	**MgO**	**Al_2O_3**	**SiO_2**	**P_2O_5**	**K_2O**	**CaO**	**TiO_2**	**FeO**	**Analytical total**
Analysis 1	0.6	0.5	23.4	61.2	1.0	2.8	2.0	0.8	7.6	64.2
Analysis 2	0.8	0.5	24.5	59.6	0.9	2.5	1.9	1.1	8.4	76.8
Analysis 3	0.7	0.4	23.7	60.1	0.8	2.5	1.9	0.9	8.9	75.5
Mean	0.7	0.5	23.9	60.3	0.9	2.6	2.0	0.9	8.3	71.0
Standard deviation (SD)	0.1	0.0	0.5	0.8	0.1	0.2	0.0	0.1	0.7	
Coefficient of variation (CV)	0.1	0.1	0.0	0.0	0.1	0.1	0.0	0.1	0.1	

Table S3.12.2: CA200181 Ceramic matrix (all values reported as normalised wt%)

**CA200181 Clean matrix**	**Na_2O**	**MgO**	**Al_2O_3**	**SiO_2**	**P_2O_5**	**K_2O**	**CaO**	**TiO**	**FeO**	**BaO**	**Analytical total**
Analysis 1	n.d.	0.7	30.3	54.8	1.0	1.7	2.2	0.6	8.6	0.2	88.5
Analysis 2	0.6	0.7	29.9	56.1	0.9	1.3	2.1	0.7	7.7	n.d.	89.3
Analysis 3	n.d.	0.7	28.6	54.9	1.3	1.8	2.5	1.2	9.1	n.d.	73.5
Mean	$\leq$0.6	0.7	29.6	55.2	1.1	1.6	2.2	0.9	8.4	$\leq$0.2	84.2
Standard deviation (SD)	n/a	0.0	0.9	0.7	0.2	0.2	0.2	0.3	0.7	n/a	
Coefficient of variation (CV)	n/a	0.1	0.0	0.0	0.2	0.2	0.1	0.4	0.1	n/a	

Table S3.12.3: CA200181 inclusions (all values reported as normalised wt%)

**CA200181**	**Na_2O**	**Al_2O_3**	**SiO_2**	**P_2O_5**	**K_2O**	**CaO**	**TiO**	**Cr_2O_3**	**MnO**	**FeO**	**SnO_2**	**Analytical total**
Ilmenite	n.d.	n.d.	n.d.	n.d.	n.d.	n.d.	55.9	n.d.	1.4	42.7	n.d.	100.0
Fe-rich mineral	n.d.	10.8	13.8	0.4	n.d.	1.0	n.d.	0.3	n.d.	73.7	n.d.	105.3
Potassium feldspar	0.4	18.8	63.4	n.d.	16.6	n.d.	n.d.	n.d.	n.d.	n.d.	0.9	72.5

13 CA200182 (GZ13)

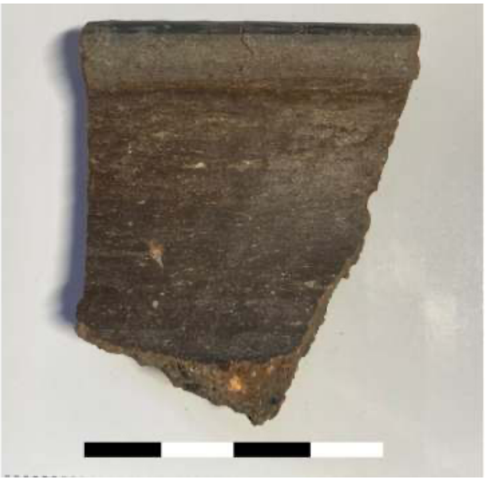

(a) Sample CA200182

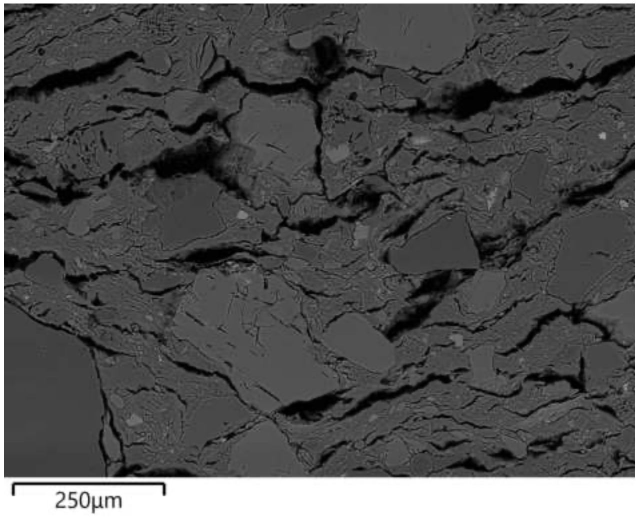

(b) Bulk ceramic (100x)

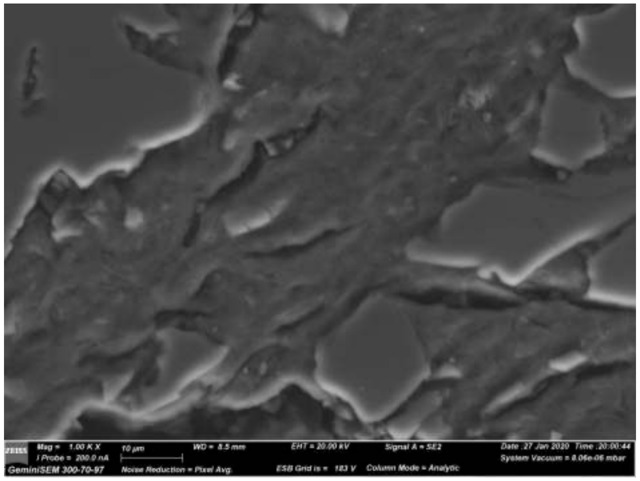

(c) Ceramic matrix (1000x)

Figure S3.13: CA200182

Table S3.13.1: CA200182 Bulk ceramic (all values reported as normalised wt%)

**Column1**	**Na_2O**	**MgO**	**Al_2O_3**	**SiO_2**	**P_2O_5**	**K_2O**	**CaO**	**TiO_2**	**FeO**	**Analytical total**
Analysis 1	1.6	0.7	19.5	67.1	n.d.	4.1	1.9	0.7	4.4	79.7
Analysis 2	1.8	0.7	19.9	65.9	0.5	4.6	1.9	0.6	4.1	73.0
Analysis 3	1.6	0.9	21.2	64.2	0.6	3.9	2.0	0.6	5.0	73.9
Mean	1.6	0.8	20.2	65.7	0.4	4.2	1.9	0.6	4.5	75.5
Standard deviation (SD)	0.1	0.1	0.9	1.4	0.3	0.3	0.0	0.1	0.4	
Coefficient of variation (CV)	0.1	0.1	0.0	0.0	0.9	0.1	0.0	0.1	0.1	

Table S3.13.2: CA200182 Ceramic matrix (all values reported as normalised wt%)

**CA200182**	**Na_2O**	**MgO**	**Al_2O_3**	**SiO_2**	**P_2O_5**	**K_2O**	**CaO**	**TiO_2**	**FeO**	**Analytical total**
Analysis 1	0.5	1.4	27.5	57.5	0.7	1.7	2.6	0.6	7.5	86.1
Analysis 2	0.4	1.6	28.4	54.4	1.0	1.4	3.2	1.9	7.6	87.5
Analysis 3	1.0	1.9	27.8	56.6	0.9	1.2	2.6	0.8	7.2	103.8
Mean	0.7	1.6	27.9	56.2	0.9	1.4	2.8	1.1	7.4	92.5
Standard deviation (SD)	0.3	0.2	0.5	1.6	0.2	0.3	0.3	0.7	0.2	
Coefficient of variation (CV)	0.5	0.1	0.0	0.0	0.2	0.2	0.1	0.6	0.0	

Table S3.13.3: CA200182 inclusions (all values reported as normalised wt%)

**CA200182**	**Na_2O**	**Al_2O_3**	**SiO_2**	**P_2O_5**	**K_2O**	**CaO**	**TiO**	**V_2O_5**	**FeO**	**ZrO_2**	**Nb_2O_5**	**Ag_2O**	**BaO**	**HfO_2**	**Analytical total**
Zircon	n.d.	0.7	30.7	n.d.	n.d.	0.6	n.d.	n.d.	2.5	64.6	n.d.	n.d.	n.d.	1.0	95.3
Ti-rich mineral	n.d.	2.5	4.1	1.6	n.d.	1.0	83.5	1.0	4.3	n.d.	1.6	0.3	n.d.	n.d.	77.4
Potassium feldspar	0.5	18.8	64.9	n.d.	15.5	n.d.	n.d.	n.d.	n.d.	n.d.	n.d.	n.d.	0.3	n.d.	95.9
Alumina silicate	n.d.	24.4	40.4	n.d.	n.d.	22.3	n.d.	n.d.	12.9	n.d.	n.d.	n.d.	n.d.	n.d.	95.7
